# SEI Formation in Sulfide-Based Solid-State Batteries: Influence of Contact Conditions on Impedance-Derived Interphase Growth Kinetics

**DOI:** 10.1021/acsami.6c07844

**Published:** 2026-06-04

**Authors:** Sascha Kremer, Christoph D. Alt, Luca Schuster, Johannes Westphal, Burak Aktekin, Jürgen Janek, Janis K. Eckhardt

**Affiliations:** † Institute of Physical Chemistry, 9175 Justus-Liebig-University Giessen, Heinrich-Buff-Ring 17, Giessen D-35392, Germany; ‡ Center for Materials Research (ZfM), Justus-Liebig-University Giessen, Heinrich-Buff-Ring 16, Giessen D-35392, Germany

**Keywords:** SEI, Wagner diffusion model, lithium metal anode, interphase formation, solid electrolyte interface, reaction kinetics, interface stability

## Abstract

The temporal evolution of the solid-electrolyte interphase (SEI) resistance in sulfide-based solid-state batteries with lithium metal anode has been shown to be well described by diffusion-controlled interphase growth. Yet, recent studies reveal that the extracted SEI rate constant estimates are highly sensitive to experimental conditions, such as stack pressure, and vary significantly depending on the electrochemical characterization method used. In this study, we evaluate SEI growth kinetics derived from symmetric cell-level impedance measurements. Through comprehensive transport simulations and experiments with the argyrodite solid-electrolyte Li_6_PS_5_Cl, we investigate how the characteristic contact conditions encountered in typical impedance studies affect the corresponding SEI rate constant estimates. We find that increasing stack and joining pressure leads to decreasing rate constant estimates, driven by an increase in the *true* contact area. *A* *pparent* saturation of SEI growth may originate from the presence of native surface passivation layers on lithium metal foil. Crucially, we highlight important uncertainties in experimental impedance data analysis and contextualize our findings by comparison with coulometric titration time analysis (CTTA). Overall, our findings contribute to the in-depth understanding of interphase growth kinetics in solid-state batteries and their quantification using impedance spectroscopy.

## Graphical abstract

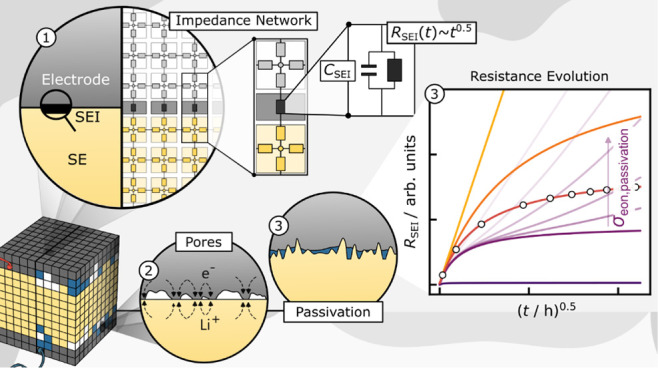

## 1 Introduction

In the technological race against climate change, solid-state batteries (SSBs) promise to be a potential advancement of conventional lithium-ion batteries (LIBs).^1,2^ However, the thermodynamic compatibility of SSB components remains a major challenge for ensuring their safe and reliable long-term operation.^3,4^ Precisely, inorganic solid electrolytes (SEs) in contact with a low-potential lithium metal negative electrode, i.e., *E*_H_[Li^+^/Li]=–3.04V vs SHE and high-potential positive electrode active material (CAM) are prone to degradation.^5–7^ Such reactions deplete active material, form resistive multiphase degradation layers, and continue to pose obstacles in developing practical SSBs.^8,9^

Depending on the partial ionic and electronic conductivities (*σ*_ion_) and (*σ*_eon_) of degradation layers, they are categorized as either *mixed conducting interphases* (MCIs, where *σ*_ion_ ≈ *σ*_eon_) or *solid electrolyte interphases* (SEIs, where *σ*_ion_ ≫ *σ*_eon_). Both, MCIs as well as SEIs, usually possess a much lower *σ*_ion_ compared to their corresponding SEs.^10^ Therefore, their continuous growth significantly increases battery impedance and reduces cycle life, with only very few exceptions like in the case of “LiPON”.^11–13^ Assuming a solid-state reaction limited by coupled ion and electron transport, MCIs are expected to grow continuously eventually causing short circuiting. In contrast, dense SEIs may exhibit self-limiting growth once a passivating layer fully covers the reactive interface. However, in practice, no SEI is perfectly homogeneous and electronically insulating.^14–17^ Therefore, interphase growth is not expected to cease entirely, but rather to decelerate over time.^18–20^

The time-dependent evolution of the SEI has been studied extensively for liquid electrolyte-based batteries, with many authors reporting a square-root-of-time dependence for the resistance growth, consistent with a diffusion-controlled interface reaction.^21–28^ In the field of solid-state batteries, the literature is comparatively scarce, but several authors have reported analogous behavior. For instance, evidence for diffusion-controlled interphase growth has been reported for sulfide and halide SEs in contact with lithium metal and CAM.^29–31^ Wenzel *et al*.^18,19^ identified these characteristics for the SEI resistance of lithium argyrodites (Li_6_PS_5_ *X*, *X* = Cl, Br, I) and Li_7_P_3_S_11_ in contact with lithium metal. A similar trend was observed by Zuo *et al*.^28^ for the interphase formation at the Li_10_GeP_2_S_12_|cathode interface. While these studies mainly rely on electrochemical impedance spectroscopy (EIS), similar results were obtained using other techniques. For instance, Aktekin *et al*.^20^ confirmed a square-root-of-time dependence for SEI growth between Li_6_PS_5_Cl and lithium metal using coulometric titration time analysis (CTTA).

In a recent work by Riegger *et al*.,^32^ a more complicated picture of SEI growth kinetics at the Li|Li_6_PS_5_Cl interface is presented. Through impedance measurements on symmetric Li|Li_6_PS_5_Cl|Li cells, they observed considerable variance in rate constants and an *apparent* saturation of SEI growth, strongly influenced by factors such as applied stack pressure and initial surface passivation of lithium foils.^33^ Sivavec *et al*.^34^ and Jeong *et al.* ^35^ reported similar observations for CTTA experiments, demonstrating a significant influence of the applied stack pressure.

Motivated by these findings, we critically evaluate SEI growth kinetics derived from impedance analysis of solid-state systems. Using a 3D impedance-network model (Figure 1), we systematically assess how the contact conditions, commonly encountered in solid state systems, affect the *apparent* interphase growth kinetics inferred from cell-level impedance measurements. We find that increasing stack and joining pressure leads to decreasing rate constant estimates, driven by an increase in the *true* contact area. *Apparent* saturation of SEI growth can arise from the presence of native surface passivation on alkali metal foils. In addition to our simulation studies, we experimentally assess the impact of contact conditions through stack- and joining-pressure-dependent impedance measurements on Li|Li_6_PS_5_Cl|Li cells. While we observe a similar saturation of resistance growth to that obtained by others^32^ and predicted by our model, we also identify critical uncertainties in the experimental data analysis. The impedance studies are contextualized through comparison with CTTA,^20^ highlighting fundamental differences between both methods.

**Figure 1:**
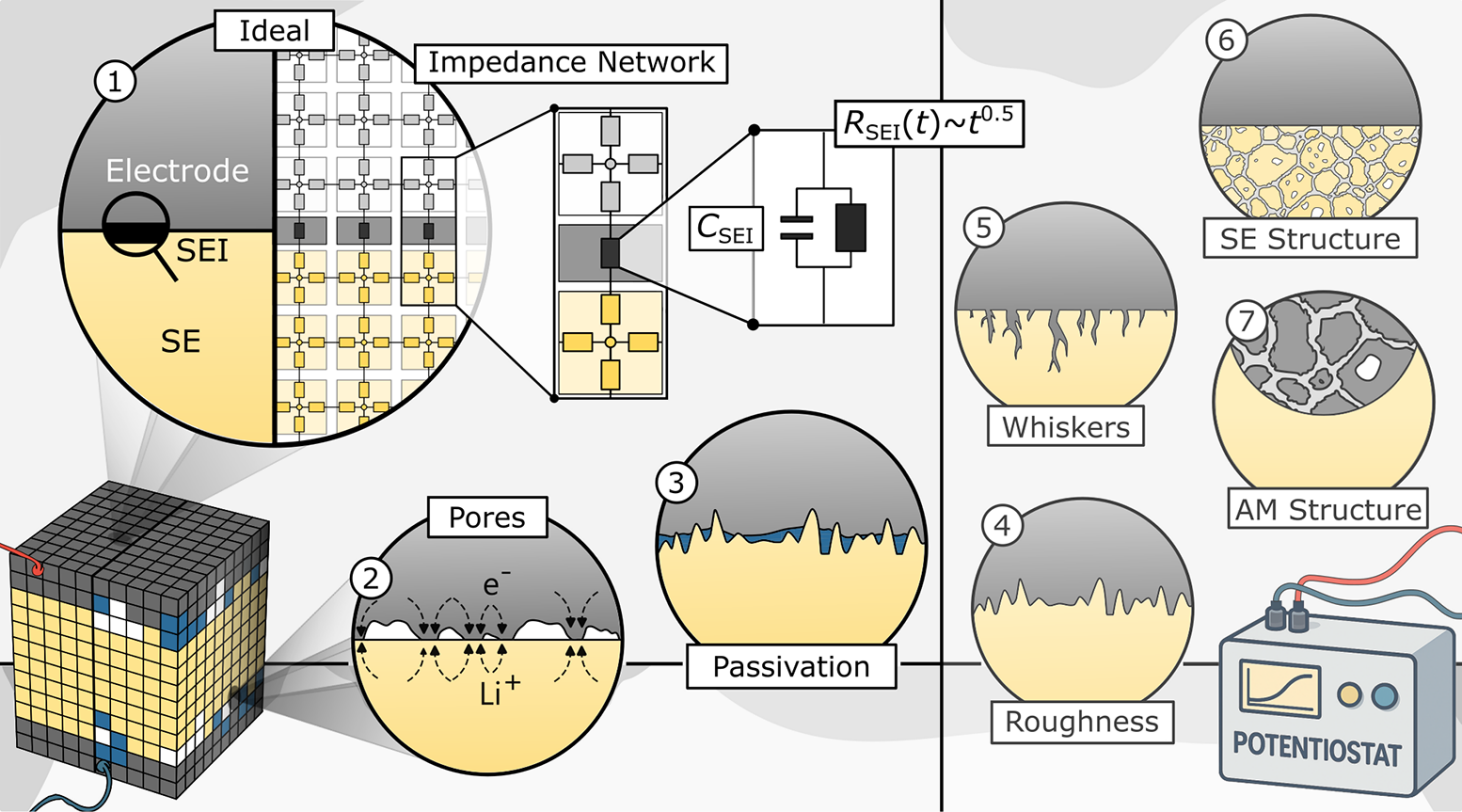
Model systems for investigating the impact of contact conditions on impedance-derived *apparent* SEI growth kinetics. The influence of different interfacial contact conditions is investigated using 3D transport simulations. Simplified geometrical model systems are translated into electrical impedance networks. The local, area-specific SEI resistance at the interface is modeled to evolve with a square-root-of-time dependence (*R*_SEI_ ∼ *t* ^0.5^). The derived SEI rate constants extracted from global^41^ cell-level impedance data are evaluated. Only the configurations to the left of the vertical line are investigated in this study (1–3); the scenarios on the right (4–7) represent additional relevant conditions not considered here.

## 2 Theoretical Background and Model Assumptions

This part outlines the principles and assumptions underlying our analysis, moving from *intrinsic*, *local* growth laws to the simulation of *global* (symmetric) cell-level behavior. In Section 2.1, we briefly summarize the Wagner model, which predicts the characteristic square-root-of-time-dependence of diffusion-controlled interface reactions. Rather than presenting a full theoretical derivation, we focus on conveying a qualitative understanding of the key assumptions involved. Afterward, in Section 2.2, we introduce the Deal-Grove model to describe how additional interlayers, such as native passivation layers found on lithium metal, modify *intrinsic*, *local* growth kinetics. Finally, Section 2.3 details how these *local* rules are integrated into 3D electric networks that we use to simulate how realistic contact conditions affect *apparent*, cell-level growth kinetics derived from impedance data.

### 2.1 Wagner Model – Diffusion-Controlled Interphase Growth in Solid-State

The basic model for diffusion-controlled interphase growth in solid-state was introduced by Wagner in 1933.^36–38^ Originally developed to explain the parabolic rate law observed for the tarnishing of metals at elevated temperatures, the model describes interfacial processes driven by fundamental solid-state processes at solid|gas interfaces.^30^ Reactants diffuse in opposite directions, localizing redox reactions to a narrow interfacial reaction zone. Once a thin but continuous product layer has been formed, further growth becomes limited by diffusion of the reactants through the product layer itself. As reactants' transport across the layer continues, material consumption persists, but at a diminishing rate. This leads to the characteristic time dependence, where growth slows down as the layer thickness increases.

In the model, the growth of reaction layers at planar solid|gas interfaces is derived under one-dimensional flux conditions. A dense, adherent, and homogeneous, product phase is assumed that fully covers the interface. The reaction layer is considered sufficiently thick to neglect space and surface charge effects. Growth is driven solely by the constant chemical potential gradient of the mobile component across the reaction layer. Electroneutrality enforces flux coupling among charged species. Thus, interphase formation is limited by the transport of the least mobile species within the reaction layer.

Under these assumptions, the growth rate of the reaction layer can be determined based on its transport properties. Adapting the model for solid|solid interfaces requires accounting for the free reaction enthalpy of the corresponding decomposition reaction. With the chemical potential gradient of the active metal (e.g., lithium or sodium) across the interphase being the reaction’s driving force, the resulting reaction layer thickness (*d*_int_) as a function of the reaction time (*t*) is given by

1 $$d_{\mathrm{int}}=\sqrt{\frac{2\cdot M_{\mathrm{int}}\cdot \sigma_{\mathrm{amb},\mathrm{int}}}{F^{2}\cdot \rho_{\mathrm{int}}\cdot x_{\mathrm{Me}}}\cdot \Delta \mu_{\mathrm{Me}}}\cdot \sqrt{t}=k\cdot \sqrt{t}$$

where *F*, *ρ*_int_, *x*_Me_, *M*_int_, *σ*_amb,int_ Δ*μ*_Me_, and *k* represent the Faraday constant, the mean mass density of the interphase layer, the stoichiometric factor (*i.e.*, number of moles of active metal required for the stoichiometric decomposition reaction), the mean molar mass of the interphase layer, the ambipolar conductivity of the interphase layer, the chemical potential difference of the active metal across the interphase, and the resulting rate constant, respectively. The ambipolar conductivity *σ*_amb,int_ represents the *effective* conductivity of the interphase for the overall redox process, limited by the slower of the two charge carriers. It is mathematically equivalent to a series connection of an ionic and electronic resistor (inverse of conductivity):

2 $$\sigma_{\mathrm{amb},\mathrm{int}}=\frac{\sigma_{\mathrm{eon},\mathrm{int}}\cdot \sigma_{\mathrm{ion},\mathrm{int}}}{\sigma_{\mathrm{eon},\mathrm{int}}+\sigma_{\mathrm{ion},\mathrm{int}}}$$

where, *σ*_eon,int_ and *σ*_ion,int_ are the partial electronic and ionic conductivity, respectively.

If *σ*_amb,int_ is known, the interphase resistance can be determined from the linear relationship between *d* vs *t* ^0.5^, and vice versa. For primarily ion-conducting interphases, *i.e*., a SEI, the area-specific ionic interphase resistance *R*_SEI_ can be estimated using *σ*_ion,SEI_ = *d*/*R*_SEI_:

3 $$R_{\mathrm{SEI}}=\frac{1}{\sigma_{\mathrm{ion},\mathrm{SEI}}}\cdot \sqrt{\frac{2\cdot M_{\mathrm{SEI}}\cdot \sigma_{\mathrm{amb},\mathrm{SEI}}}{F^{2}\cdot \rho_{\mathrm{SEI}}\cdot x_{\mathrm{Me}}}\cdot \Delta \mu_{\mathrm{Me}}}\cdot \sqrt{t}=k^{\prime }\cdot \sqrt{t}$$

Here, *k′* reflects the rate constant for the areal SEI resistance growth. Revisiting eq 1 and eq 3 reveals that *k* and *k*′** are connected by *σ*_ion,SEI_:

4 $$k=k^{\prime }\cdot \sigma_{\mathrm{ion},\mathrm{SEI}}$$

If one partial conductivity dominates, *k* and can be simplified by considering only the lower, rate-limiting partial conductivity in the square-root-term. In the case of an SEI in SSBs, where *σ*_ion_ _,SEI_ ≫ *σ*_eon,SEI_ electronic transport primarily governs SEI’s thickness increase. The ionic conductivity, in contrast, determines the SEI’s resistance at a given thickness *d*.

Notably, this study is focused on the Li|Li_6_PS_5_Cl interface as a model system. In Section S1 of the Supporting Information, we derive the reaction’s driving force (*i.e*., Δ*μ*_Li_) for interphase formation using thermodynamic data. If all transport properties are known, eq 1 and eq 3 can be applied to predict interphase growth (*i.e*., *d*_SEI_) and the associated resistance contribution (*i.e.*, *R*_SEI_) to evaluate its impact on overall cell resistance and long-term performance of SSBs.^27,39^ This approach, including the derivation of the reaction’s driving force, is readily adaptable to other model systems and cell chemistries beyond lithium metal and Li_6_PS_5_Cl.

### 2.2 Deal-Grove Model – The Impact of Additional Interlayers

In contact regions, where a SEI is separated from the electrode by an additional interlayer, *e.g*., native surface passivation on alkali-metal foils, SEI growth will remain but at a different rate. Assuming that the Wagner-type growth mechanism remains valid (ambipolar diffusion limited), the presence of an additional resistive layer slows down *local* SEI growth. For a contact containing an additional passivation layer of thickness *d*_p_ and an ambipolar conductivity *σ*_amb,p_ in series with the growing SEI, the SEI thickness *d*_SEI_ evolves as (see Section S1 of the Supporting Information for derivation):

5 $$d_{\mathrm{SEI}}=\sigma_{\mathrm{amb},\mathrm{SEI}}\cdot \left[-\frac{d_{p}}{\sigma_{\mathrm{amb},p}}+\sqrt{{\left(\frac{d_{p}}{\sigma_{\mathrm{amb},p}}\right)}^{2}+\frac{2\cdot M_{\mathrm{SEI}}\cdot \Delta \mu_{\mathrm{Me}}}{F^{2}\cdot \rho_{\mathrm{SEI}}\cdot x_{\mathrm{Me}}\cdot \sigma_{\mathrm{amb},\mathrm{SEI}}}\cdot t}\right]$$

which is a passivation layer adapted version of the Deal-Grove^40^ model. For short times, eq 5 is dominated by the constant ambipolar resistance of the passivation layer (*i.e*., the first summand in the square-root-term) and the reaction current is roughly constant, *i.e*., *d*_SEI_ becomes a linear function of time:

6 $$d_{\mathrm{SEI}}\approx \frac{M_{\mathrm{SEI}}\cdot \sigma_{\mathrm{amb},p}\cdot \Delta \mu_{\mathrm{Me}}}{F^{2}\cdot \rho_{\mathrm{SEI}}\cdot x_{\mathrm{Me}}\cdot d_{p}}\cdot t=k_{\mathrm{early}}\cdot t$$

For long times, the second summand in the square-root-term dominates and eq 5 reduces to eq 1, thus the square-root-of-time-dependence is recovered (*d*_SEI_ ≈ *k*_late_·*t* ^0.5^). In this case, the SEI layer dominates the ambipolar resistance and therefore limits its own growth. If the ionic conductivities control the *measured* resistance, the area-specific resistance evolution of the contact spot is given as

7 $$R_{\mathrm{spot}}\approx R_{\mathrm{ion},\mathrm{spot}}=\frac{d_{p}}{\sigma_{\mathrm{ion},p}}+\frac{d_{\mathrm{SEI}}}{\sigma_{\mathrm{ion},\mathrm{SEI}}}$$

Accordingly, the ionic resistance of a single prepassivated contact spot is expected to increase linearly at first, before transitioning to a square-root-of-time dependence. Comparable trends are also expected when considering an initially surface-controlled reaction.^40^

### 2.3 Heterogeneous Contact and *Apparent* Growth Kinetics – Impedance Network Simulations

In the previous analytical models, interphase resistance evolution is derived for a morphologically stable, planar interface. However, actual interfaces in SSBs – or even in simplified symmetric cells – often deviate substantially from these idealized conditions, as summarized in Figure 1: The ideal case of a flat, continuous contact (1) is never encountered in practice. Instead, interfacial pores (2), partially penetrated native passivation layers (3), and surface roughness (4) lead to complex, spatially heterogeneous contact geometries. Additional factors such as lithium dendrite or whisker formation (5), the microstructure of the solid electrolyte near the interface (6) further contribute to this complexity. When extending the analysis to CEI formation on the cathode side, the system becomes even more intricate due to the heterogeneous surface chemistry of cathode active materials and the inherently more complex electrode geometry (7).

In an impedance measurement, these heterogeneities produce a nonuniform current distribution that depends on both, time and excitation frequency. Consequently, extracted parameters and growth trends represent *effective*, morphology-weighted properties of the overall interface rather than *intrinsic*, *local* properties of the SEI. This distinction becomes particularly relevant given that the actual interface morphology, including contact area, composition, and mechanical integrity, is typically unknown. Unfortunately, it is often unclear how this affects the corresponding *global* growth trends and rate constant estimates.

To address these questions, we consider several simplified 3D model systems with contact imperfections and simulate the *global,* cell level impedance spectra and corresponding growth trends. Our analysis includes: (1) an intimate planar contact, serving as the ideal reference case, (2) a contact with interfacial pores, and (3) punctured passivation layers (revisit Figure 1). Each model comprises a square cross-sectional area (*L*_x_ = *L*_y_ = 100 μm and consists of a 100 μm thick SE layer in contact with a 20 μm thick lithium metal electrode. The *apparent* electrode area (*A*_electrode_) corresponds to the full sample surface, assuming ideal mechanical stability and contact. For simplicity, the SE is treated as dense and free of microstructural features. The interface morphology is varied and systematically explored throughout the simulation study, with details specified in each section.

For the impedance simulations, we use an electric network model where each model system with its specific interface morphology is discretized and represented as a voxel-based geometry. Ion-transport between voxel centers is modeled by *local* equivalent circuit elements, each defined by the phase or interface it belongs to (see Figure 1). In the modeling approach, interphase growth is treated implicitly by introducing *local*, time-dependent interphase resistors and capacitors with area-specific resistances and capacitances, *R*_SEI_, and *C*_SEI_, as described by eq 3 and eq 7. The change in *local* parameters mimics the increase in interphase thickness *d*_SEI_ over time as derived by the *local* models from the previous sections, while the *local* transport properties (*σ*_ion,SEI_, *ε*_SEI_) remain constant.

To ensure clarity throughout this study, we distinguish between two rate constants:

- ***Intrinsic*** (*k* ^′^): The fundamental, *true* rate of SEI growth occurring at a specific, *local* contact point, calculated by eq 3 or eq 7.
- ***Apparent*** $\left(k_{\exp}^{\prime }\right)$ : The *global* rate constant derived from the cell-level impedance, normalized to the *apparent* electrode area *A*_electrode_. This value is susceptible to contact heterogeneities and systematically deviates from the *intrinsic* rate *k*′** , rendering it an *effective* parameter rather than a pure material property.

Our modeling approach implies no lateral coupling between SEI growth at different contact spots as well as 1D planar SEI growth. Thus, the reaction current that drives SEI formation (under OCV conditions) is solely defined by the *local* ambipolar conductivity (thin SEI compared to contact width). The impedance, instead, is a *global* property given by the ensemble of all resistive and capacitive components of the cell and the applied *global* voltage. Finally, we assume that the measured resistance is controlled by ionic conductivity. This means that the interphase behaves as a true SEI, as expected for the interphase formed between Li_6_PS_5_Cl and lithium metal.

While these simple models cannot fully capture the complexity of real solid-state systems, we believe that they can offer valuable insight into how interfacial imperfections on the micro- to mesoscale will manifest in the impedance evolution of the overall system, *i.e*., on the cell level, and consequently affect *apparent* growth trends. A more detailed model description is provided in the Computational Details section. Further information about the electrical network model and impedance spectrum calculations are available in our previous publication.^42^

## 3 Results and Discussion

### 3.1 Simulations – Contact Conditions and Impedance-Derived Interphase Growth Kinetics

#### 3.1.1 Homogeneous Interfaces without Interlayers

To outline the analysis methodology, we begin with an idealized scenario: a planar interface without any interfacial defects, as illustrated schematically in Figure 2a. In the simulations, we assume interphase growth at each contact point at the electrode|SE interface according to eq 3. The resulting time-series of the cell-level impedance is used to extract the *apparent* rate constants $\left(k_{\exp}^{\prime }\right)$ . Three different *intrinsic* interphase rate constants *k*′** are considered, ranging from $1k_{\mathrm{ref}}^{\prime }$ , to $3k_{\mathrm{ref}}^{\prime }$ , with $k_{\mathrm{ref}}^{\prime }=1.4{\Omega \cdot \mathrm{cm}}^{2}\cdot s^{-0.5}$ , as a reasonable estimate from previous work. We model the impedance at different times, starting from *t*_0_, when interphase growth is initiated by establishing contact between materials, but no interphase has been formed yet, and progressing to *t*_3_, the final stage of the simulation.

**Figure 2:**
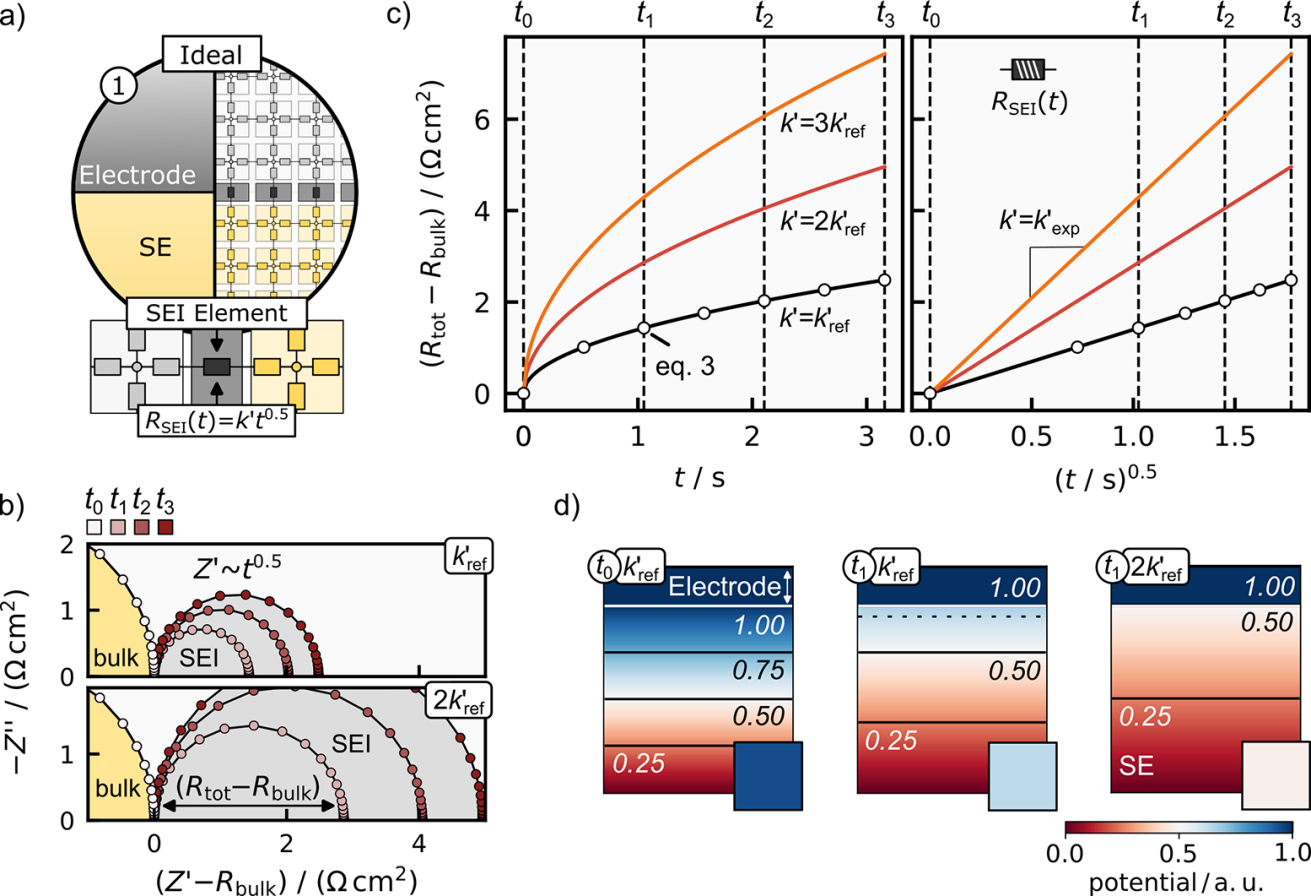
Analysis of SEI resistance growth at a planar, idealized interface. (a) It is assumed that the electrode and the SE are homogeneous, and that the increase in interphase thickness and intrinsic areal interphase resistance (*R*_SEI_) at the interface follow a square-root-of-time dependence. (b) The bulk impedance (yellow) remains constant over time, while the interphase impedance (gray) increases at different rates depending on the assumed intrinsic growth rate *k′*. (c) The intrinsic rate *k* determines the increase in cell-level resistance (*R*_tot_ *–R*_bulk_) over time, or the linear slope when plotted against the square-root-of-time. The resistance difference is anticipated to be indicative for interphase growth. (d) Transport through the system is quasi-1D as indicated by the uniform potential drop along the transport direction and the constant in-plane potential (see inset) below the interface (dashed line).

In experimental practice, impedance data is normalized to the geometric electrode area (*A*_electrode_), assuming a planar interface of perfect, intimate contact between electrode and sample. Unfortunately, obtaining reliable measurements of the *true* contact area *A* in experiments is challenging and, in most cases, not feasible. Therefore, we adapt this experimental practice and normalize all simulated impedance values to *A*_electrode_ (*i.e*., the *apparent* electrode area), despite the potential mismatch with the *true* contact area *A*.

Figure 2b shows the time-dependent impedance evolution in Nyquist representation. At the starting point *t*_0_, the impedance spectrum displays only a bulk semicircle (yellow), indicating the absence of an interphase. To better visualize subsequent impedance changes, the bulk resistance (*R*_bulk_) is subtracted from the real part of the impedance (*Z* ^′^), effectively shifting the spectra along the *Z*′-axis. With increasing time, a second semicircle emerges and increases in size. It reflects interphase growth, which is governed by *k* ^′^ as simulation input parameter. Higher values of *k* ^′^ result in a more rapid increase in interphase thickness and corresponding interphase resistance, as illustrated by the comparison between the $1k_{\mathrm{ref}}^{\prime }$ and the $2k_{\mathrm{ref}}^{\prime }$ simulation series. Notably, the bulk signal remains constant over time, confirming that the observed impedance changes originate solely from the evolving interphase.

To obtain mechanistic insights into interphase evolution, either the total resistance (*R*_tot_) or the *apparent* SEI resistance is typically plotted as a function of time.^18–20^ Isolating the SEI resistance requires a detailed understanding of the underlying impedance features, which is challenging for complex experimental data.^32,33^ For symmetric solid-state cells with planar electrodes, it is reasonable to assume that all impedance contributions other than the bulk and potential grain boundary impedances originate from the electrode|SE interface. In the case of the Li|Li_6_PS_5_Cl interface, this assumption is further justified due to the negligible charge transfer resistance (*i.e*., high exchange current density).^43,44^ Consequently, changes in the resistance difference (*R*_tot_–*R*_bulk_) are anticipated to be indicative for SEI growth (*i.e*., labeled “SEI” in Figure 2b).

Figure 2c shows the resistance difference (*R*_tot_–*R*_bulk_) as a function of time (vs *t* and *t* ^0.5^) for different *k* ^′^. Notably, directly calculating the area-specific SEI resistance *R*_SEI_(*t*), according to eq 3 (open circles) agrees with the resistance difference (*R*_tot_–*R*_bulk_) derived from the impedance analysis approach (black solid line, $k_{\exp}^{\prime }=k^{\prime }$ ). Obviously, this only holds true for this idealized interface, but not necessarily for more complex interface morphologies.

The slope of the different resistance curves changes with the *intrinsic* interphase rate constants *k* ^′^: Higher values lead to a steeper resistance increase. To extract $k_{\exp}^{\prime }$ from a measurement, the data is typically linearized by plotting the respective resistance – here, (*R*_tot_–*R*_bulk_) – over *t* ^0.5^, as done in the right graph of Figure 2c. The slope obtained from linear interpolation of the resistance curves corresponds to the *apparent* rate constant $k_{\exp}^{\prime }$ . For this idealized interface, $k_{\exp}^{\prime }$ values match with the respective *intrinsic* simulation input values *k*′.

Homogeneous SEI (resistance) growth is also reflected in the DC electrical potential distribution. Figure 2d shows the evolution of the DC potential within the system for different *k* ^′^ at various time points (*t_i_*). A cross-section along the transport direction reveals a negligible potential drop in the metal electrode (upper dark blue region), attributed to its high electronic conductivity. Across the SE, the potential drop remains uniform, gradually transitioning to the predefined potential difference between electrodes. The forming interphase layer causes an increasing fraction of the potential drop to be localized at the electrode|SE interface, which is reflected in the shifting equipotential lines within the SE. The higher the SEI resistance, the more pronounced this potential drop becomes, as seen at *t*_1_ for different *k* ^′^.

Transport across the system is quasi-1D, as indicated by the uniform in-plane potential distribution perpendicular to the transport direction just below the electrode (dashed line, see insets in Figure 2d). However, this idealized behavior and the resulting agreement between *apparent* and *intrinsic* rate constants ( $k_{\exp}^{\prime }$ and *k* ^′^, respectively) cannot be expected for interface conditions typically found in solid-state systems.

#### 3.1.2 The Influence of Pores, Contact Area, and Current Constriction

One of the main challenges in solid-state is achieving intimate contact between two solid materials, such as the electrode and the SE. In most practical cases, residual pores at the interface lead to *true* contact areas *A* smaller than the *apparent* electrode area *A*_electrode_. This introduces a more complex transport behavior and challenges in data interpretation.^45–47^

To explore the impact of interfacial pores (or more generally, insulating interfacial phases), we consider a model system featuring a single square-shaped contact spot (Figure 3a). This contact spot is surrounded by an insulating phase along the edges of the simulation domain. In the simulation series, we systematically vary the *relative* contact area (*A*_r_) from 100% (perfect contact) down to 10% (minimal contact) of *A*_electrode_. All transport properties remain constant across different simulations, with $k^{\prime }=1k_{\mathrm{ref}}^{\prime }$ . Notably, interphase growth is restricted to the regions where the electrode is in contact with the SE. As in experimental studies, where detailed information about the *true* contact area *A* is limited, the impedances and extracted resistances are normalized to *apparent* contact area *A*_electrode_.

**Figure 3:**
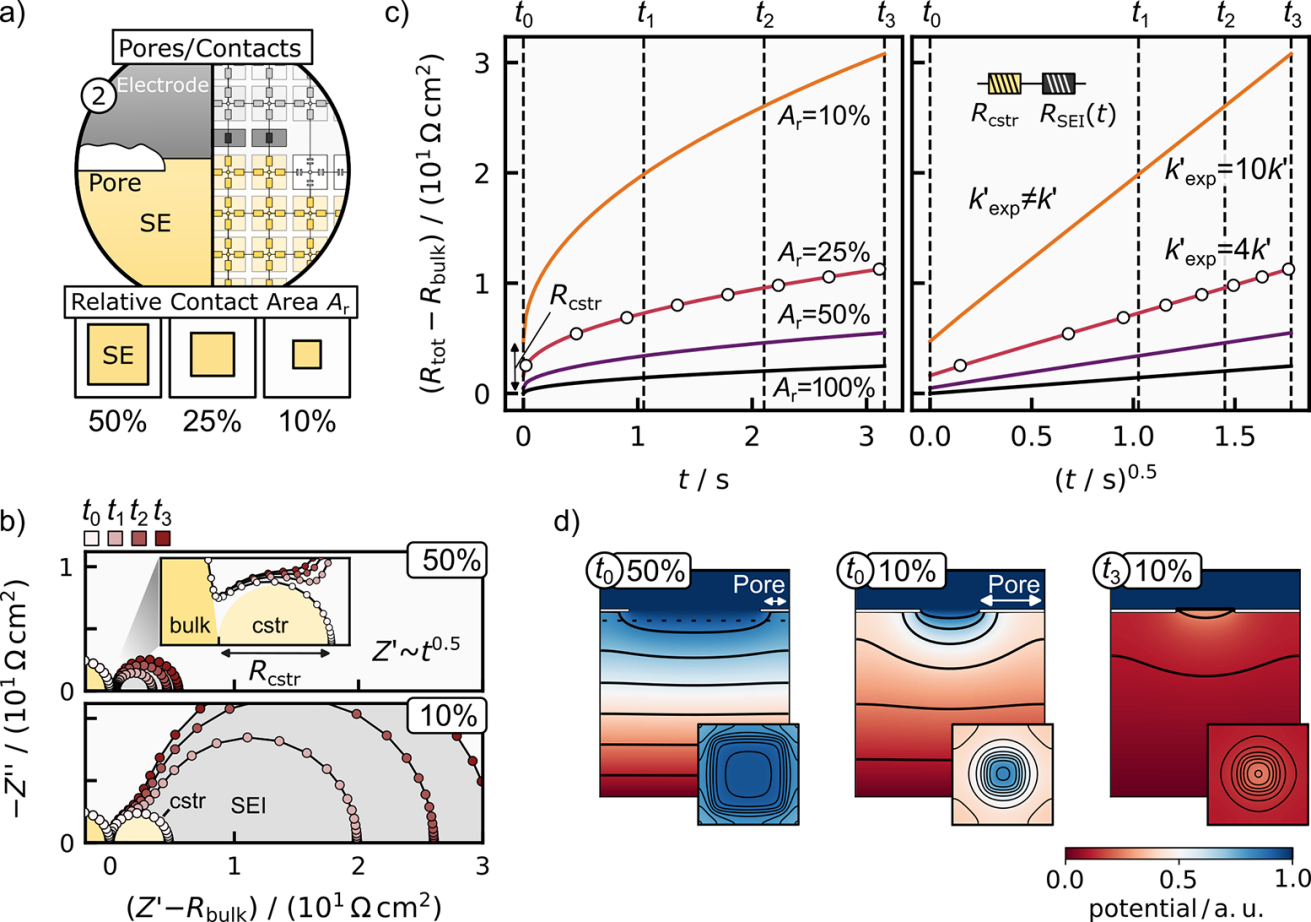
Influence of the true contact area and current constriction on apparent sei resistance growth. The intrinsic interphase rate constant *k* ^′^ was kept constant in the simulations. (a) The relative contact area *A*_r_ between the homogeneous metal electrode and the SE is systematically varied by changing the size of a square-shaped contact spot surrounded by a pore phase. (b) The resulting constriction effect can appear as a distinct signal in the impedance spectrum. The magnitude of the two resistances (*R*_cstr_, (*R*_tot_–*R*_bulk_)) depends on *A*_r_. (c) The apparent growth rate $k_{\exp}^{\prime }$ (*i.e*., slope of the resistance curves) differs from the intrinsic rate *k* ^′^ due to incorrect normalization of the resistance with respect to *A*_electrode_ rather than *A*_r_ (see black curve as a reference). (d) Current paths through the system are multidimensional, as indicated by the inhomogeneous potential drop along the transport direction and within a layer near the interface (dashed line).

Figure 3b shows that when adding interfacial pores, an additional impedance contribution appears in the spectrum, besides the “bulk” and “SEI” signal. It originates from current constriction (“cstr”) at the electrode|SE interface.^48–50^ The constriction signal does not always appear as a distinct contribution in the impedance spectrum. Instead, it may also overlap with the bulk or SEI signal, depending on the characteristic frequency of the processes.^50^ In the case of current constriction due to interfacial pores, the characteristic frequency is a function of the pore depth, spatial pore distribution and SE conductivity.^50^ Only wide, shallow pores are presumed to have capacitances large enough to make the constriction signal separable from the bulk signal.^48^

The comparison of the different simulation series shows that the constriction resistance (*R*_cstr_) and the *apparent* SEI resistance, represented by (*R*_tot_–*R*_bulk_), are sensitive to *A*_r_ variations. *R*_cstr_ increases in size as *A*_r_ decreases, while the increase of (*R*_tot_–*R*_bulk_) over time is enhanced with decreasing *A*_r_. This is also evident when considering the *t*-dependence of (*R*_tot_–*R*_bulk_), as shown in Figure 3c. Compared to the ideal reference curve for *A*_r_ = 100%, discussed in Section 3.1.1, two key differences emerge: (*i*) the individual resistance curves exhibit an offset along the resistance-axis, and (*ii*) $k_{\exp}^{\prime }$ is significantly larger for smaller *A*_r_, although the underlying *k* ^′^ remains unchanged across the simulations. The parameters $k_{\exp}^{\prime }$ (eq 3) and *R*_cstr_ can be extracted from the time dependence of (*R*_tot_–*R*_bulk_) using a simple series equivalent circuit model, as shown in the right graph of Figure 3c (equivalent circuit fit: white circles).

The vertical offset of the resistance curves along *y*-axis is due to *R*_cstr_. It resembles the reduction in SE conduction volume close to the interface. This is evident in the DC potential distribution, both at *t*_0_, before any SEI formation, and at *t*_3_, when an SEI layer has formed (Figure 3d). Along the transport direction, the equipotential lines near the interface exhibit distinct curvature, while the in-plane potential distribution below the interface (dashed line) becomes inhomogeneous. Interfacial pores induce complex transport behavior, giving rise to curved 3D current pathways and a corresponding increased potential drop close to the interface.

However, the main discrepancy between the *apparent* $k_{\exp}^{\prime }$ and the *intrinsic* *k*′ arises simply from a systematic error in data normalization. As discussed in detail in Section 3.1.1, experimental impedance data are commonly normalized to *A*_electrode_, implicitly assuming an intimate contact with the SE (*i.e*., *A* = *A*_electrode_). However, this is rarely guaranteed for solid|solid interfaces (*i.e*., *A* < *A*_electrode_). According to eq 3, *R*_SEI_ is inversely proportional to *A* rather than *A*_electrode_, since *R*_SEI_ is an *area-specific* resistance. Consequently, the incorrect normalization of (*R*_tot_–*R*_bulk_) leads to the different slopes of the resistance curves shown in Figure 3c.

As a result, $k_{\exp}^{\prime }$ reflects an *apparent* value rather than the *intrinsic* *k* ^′^. Its dependence on *A* makes it challenging to distinguish whether changes in the slope of resistance curves arise from variations in *A* or from genuine changes in the underlying SEI growth kinetics. For instance, if *A*_r_ is only 10% of *A*_electrode_, $k_{\exp}^{\prime }$ will overestimate *k* ^′^ by an order of magnitude, as evident from eq 3. While this may sound trivial, it represents a major challenge for reliably assessing SEI growth kinetics since the *true* contact area, *A*, in solid-state systems is rarely known, difficult to quantify, and may even evolve during operation.

#### 3.1.3 Uncertainty in the True Contact Area – the Influence of the Contact Distribution

The uncertainty in the *true* contact area *A* is a fundamental challenge in solid-state systems and intensively discussed in contact physics literature.^51–53^ While external pressure can influence *A* to some extent, its exact value often remains unknown and is often only a small fraction of *A*_electrode_.^54^ One approach to estimate *A* is to correlate it with *R*_cstr_. As shown in the previous section, *R*_cstr_ may appear as an additional contribution in the impedance data, *i.e*., as an additional offset in a plot of (*R*_tot_–*R*_bulk_) vs *t*. Theoretical models from electrical contact literature provide estimates for *A* based on *R*_cstr_ for simple model geometries.^55^ However, *R*_cstr_ is highly dependent on the spatial contact distribution, which in practice makes resistance measurements unsuitable to determine *A*.^48,55,56^

This has direct implications for the interpretation of impedance data and the extraction of $k_{\exp}^{\prime }$ . Even if the initial impedance spectra of two symmetric cells seem similar, the underlying interfacial contact area may differ substantially. Although, these differences in *A* are not directly observable in the initial impedance, they result in significantly different values of $k_{\exp}^{\prime }$ , *i.e*., a faster growth of the impedance. For instance, *A_r_* might vary between 20% and 100% of *A*_electrode_ without noticeable evidence in the electric data, other than an *apparent* increase of $k_{\exp}^{\prime }$ , *i.e*., faster *apparent* interphase growth.^48,57^

For the interested reader, we show and discuss the influence of the contact distribution on our specific model case in Section S3 of the Supporting Information. Here, the contact distribution influences the cell impedance (*i.e*., *R*_cstr_), but not the extracted $k_{\exp}^{\prime }$ .

#### 3.1.4 The Influence of Native Surface Passivation and Interlayers

A practical way to address the uncertainty in *true* contact area is to study the effect of mechanical pressure on *apparent* growth trends.^32^ Increasing pressure typically enhances the *true* contact area. As the *true* contact area approaches the *apparent* one, the corresponding *apparent* rate constant extracted should likewise converge toward its *intrinsic* value. Complementary techniques such as cross-sectional SEM analysis can further help to verify sufficient mechanical contact.^58^

However, even if decent mechanical contact is ensured another uncertainty may persist: Reactive alkali metals like lithium or sodium rapidly form native passivation layers upon exposure to trace atmospheric gases such as nitrogen, oxygen, water vapor, and carbon dioxide.^59^ Even gas residues in the parts-per-million (ppm) range, commonly present in glovebox atmospheres, are sufficient to form passivation layers tens of nanometers thick.^58^ Depending on their thickness and mechanical properties, these passivation layers may be partially penetrated by surface asperities of the SE during cell assembly^33,58^ and may therefore substantially affect the *apparent* growth kinetics.

To investigate to which extent such passivation layers influence *apparent* SEI growth trends, we adapt the geometric model system from Section 3.1.2. with *A*_r_ = 50% (see Figure 4a). However, this time the contact spot is surrounded by a passivation phase with a nonzero ionic conductivity, *σ*_ion,p_, *i.e*., finite ionic resistance *R*_ion,p_. Throughout the simulation series, we vary *R*_ion,p_ between *R*_ref_/100 to 400*R*_ref_, where *R*_ref_ = 9 Ω·cm^2^. To simplify the model, we first assume that the ionically conducting passivation layer has a negligible electronic conductivity compared to the SEI (*R*_p_ = *R*_ion,p_). Accordingly, SEI is only formed at the pristine contact spot, and the prepassivated contact points are modeled with time independent resistors and capacitors (revisit eq 5-eq 7).

**Figure 4:**
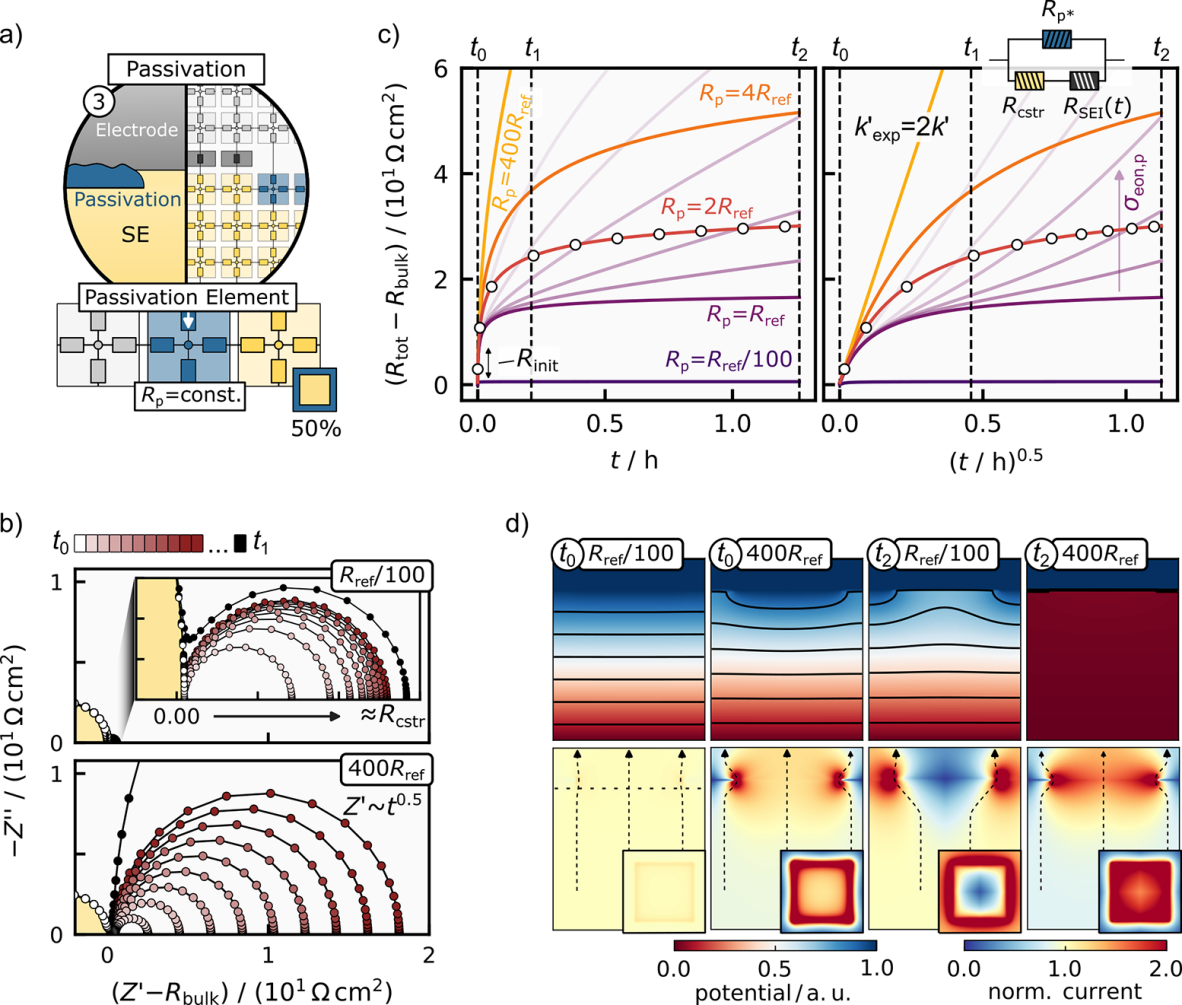
The effect of a partially penetrated interlayer on the apparent SEI resistance growth. In the simulations, *A*_r_ at the interface was kept constant at 50% of *A*_electrode_. (a) The homogeneous interlayer is penetrated by the SE, forming a square-shaped contact spot. (b) The impedance growth of the interface signal shows significant differences depending on electric properties *R*_p_ of the passivation layer. (c) The evolution of (*R*_tot_–*R*_bulk_) shows a saturation when *R*_p_ = const. When the electronic conductivity of the passivation layer (*σ*_eon,p_) becomes significant, the resistance does not saturate anymore (purple transparent lines). (d) Switching between current transport pathways (across the SEI or passivation layer) results in a resistance plateau over time. The time to reach the plateau depends on the electrical properties of the interlayer and the surface coverage (see Figure S2 of the Supporting Information).

The resulting impedance spectra for a highly conductive passivation (*R*_p_ = *R*_ref_/100) and a highly resistive passivation (*R*_p_ = 400*R*_ref_), are shown in Figure 4b. Both impedance series reveal a bulk signal alongside an interfacial contribution, which varies significantly depending on *R*_p_ and *t*. For the simulation with *R*_p_ = *R*_ref_/100, the assigned bulk signal does not solely include bulk contributions at *t*_0_, but is slightly larger (see inset: *Z* ^′^ is larger than 0, after subtraction of *R*_bulk_). This is due to transport across the passivation layer, which does not always appear as a separate signal in the impedance spectrum. In the case of *R*_p_ = 400*R*_ref_, the initial interface signal is significantly larger, and the size matches that of *R*_cstr_ for *A*_r_ = 50%, discussed for the pore case in Section 3.1.3. This shows that initially only a negligible amount of current flows through the passivation which effectively acts like a pore.^50,60^

With increasing time, an additional signal appears in the Nyquist diagram. The assignment of this contribution solely to the SEI, like done before, is not possible, since it also includes impedance contributions from current flow through the passivation. For *R*_p_ = *R*_ref_/100, the interface signal in the impedance spectra remains minimal over time. As shown in the enlarged inset, the signal exhibits only a slight initial increase before converging toward the constriction resistance (*R*_cstr_) of the low resistive contact spot (*R*_p_ ≪ *R*_cstr_). Impedance growth at *t*_1_ has more or less completely saturated. A different trend is observed for *R*_p_ = 400*R*_ref_. In this case, the impedance increase does not saturate in the simulated time frame.

Figure 4c shows the resistance growth over time for different *R*_p_. Except for the case with *R*_p_ = *R*_ref_/100, initially all curves follow a square-root-of-time dependence that gradually levels off into a plateau. This is particularly evident in the linearized representation (vs *t* ^0.5^), where the initial linear increase transitions into saturation. Notably, the transition time *t*_trans_ depends on the magnitude of *R*_p_ and the areal coverage of the interface with passivation layer: The higher the *R*_p_, the later the transition occurs. In the simulated time frame, the resistance in the highly conductive case (*R*_p_ = *R*_ref_/100) instantly enters the plateau region, while no transition is observed in the highly resistive case (*R*_p_ = 400*R*_ref_). Similar results are observed considering the extent of interface coverage: With increasing interface coverage by passivation, *t*_trans_ increases. A more detailed discussion on this, including simulation results, is given in Section S4 of the Supporting Information.

As for the previous cases, the respective growth curves can be well reproduced using a simple equivalent circuit as shown by the white circles in Figure 4c. The corresponding equivalent circuit is depicted in the right upper corner of the square-root-of-time representation. The circuit includes two paths: the SEI path with a time dependent *R*_SEI_(*t*) in series to a time-independent *R*_cstr_ (constriction resistance associated with current flow to SEI spots) and a parallel passivation layer path with a constant passivation path resistance *R*_p∗_. Here, *R*_p∗_ includes the passivation layer resistance *R*_p_ in series to the corresponding constriction resistance *R*_cstr,p_. Since both resistances are time-independent, they cannot be distinguished.

The resistance trends result from the competitive transport pathways at the interface and a resulting switch between preferred transport pathways over time, *i.e*., transport across the passivation/interlayer or across the forming SEI at the electrode|SE contact. This is reflected in the DC potential and current distribution, as shown in Figure 4d. In the highly conductive case (*R*_p_ = *R*_ref_/100) at *t*_0_, the potential and current distribution are almost uniform across the interface, closely resembling the ideal scenario of a planar interface without passivation layer (see Figure 2d). With an increase in SEI thickness, a shift in transport pathways occurs. At some point, all current flows through the passivation layer (see schematic black current lines in Figure 2d). Therefore, the extracted resistance difference (*R*_tot_–*R*_bulk_) reaches a plateau. The corresponding resistance reflects the areal-specific *R*_p_ (weighted by the relative coverage with passivation) plus the corresponding *R*_cstr_.

In the highly resistive case *(R*_p_ = 400*R*_ref_), no major changes in the current pathways are observed in the simulated time frame. All the current flows through the growing SEI layer (see schematic black current lines in Figure 2d). As a result, the square-root-of-time dependence is sustained at the cell-level, but with a two times larger *apparent* rate constant $k_{\exp}^{\prime }=2k^{\prime }$ (due to *A*_r_ = 50%). The preferred transport pathway at any given *t* depends on the resistance ratio between *R*_SEI_(*t*) and *R*_p_, as well as the induced *R*_cstr_. For large values of *R*_p_ or small areal coverage with passivation, the resistance plateau shifts to larger *t* (*i.e*., outside the simulation time frame).

The dependence of the resistance evolution on passivation layer properties highlights the critical role of measurement time frame. In experiments, it is unfeasible to capture the “complete” resistance curve over an infinite timespan. Instead, only discrete snapshots can be recorded, constrained by experimental time scales and delays between cell assembly and the onset of measurements. As a result, the measured data may either directly display fully developed plateau-like regions—reflecting passivation layer properties—or fail to capture the transition region within the available time frame. In the latter case, a larger *apparent* rate constant $k_{\exp}^{\prime }$ will be obtained. Reported literature values for passivation layer thicknesses and their transport properties on alkali-metals show large uncertainty and are likely not easily comparable between different research groups, gloveboxes or even samples. Assuming a negligible *σ*_eon,p_, the estimated *t*_trans_ for passivated lithium foils can vary widely from a few seconds up to several years, depending on the specific passivation layer properties (see detailed discussion in Section S5 of the Supporting Information).

#### 3.1.5 The Impact of the Electronic Conductivity of Interlayers

In practice, the partial electronic conductivity, σ_eon,p_, of a native passivation layer (*i.e*., an interlayer once buried in contact with a SE) is never strictly zero. Consequently, SEI growth will also occur at prepassivated contact spots, albeit, initially, at a reduced rate. In Section S6 of the Supporting Information, we present simulation results based on the analytical Deal-Grove model detailed in Section 2.2. For the parameters used in Section 3.1.4 and a passivation layer with a thickness of 10 nm, the partial electronic conductivity must be less than 10^–12^ S·cm^–1^ (*R*_eon,p_ must exceed 10^6^ Ω·cm^2^) to restrict the total resistance increase of the prepassivated spots to less than 10% of the initial passivation layer resistance over the simulation time frame of ≈1 h. However, even with low electronic conductivity, SEI growth is expected to persist underneath the passivation layer, initially progressing slowly and linearly (revisit eq 6). At long times, this newly formed SEI thickens to the point where its ambipolar resistance overtakes the ambipolar resistance of the static passivation layer underneath. When this transition occurs, the prepassivated spots finally shift into an SEI-limited regime, exhibiting the same square-root-of-time dependence and locally the same rate (*intrinsic* rate constant, *k* ^′^).

The light purple lines in Figure 4c show the (ionic) resistance development for a contact model that accounts for prepassivated contact spots with a significant σ_eon,p_ in parallel to initially pristine contact spots (σ_eon,p_ = 1·10^–11^ S·cm^–1^ to 1·10^–9^ S·cm^–1^ with 45 nm passivation layer thickness). For sufficiently high values of σ_eon,p_, the passivation layer loses its protective properties entirely, and the resistance evolution rapidly transitions into a square-root-of-time dependence.

#### 3.1.6 Lateral Crosstalk between SEI Growth at Different Contact Spots

Our previous simulation results rely on the assumption that the SEI thickness *d*_SEI_ is small compared to the distance between spots with different SEI thicknesses. At some point of an experiment, however, this assumption may fail, leading to lateral crosstalk between prepassivated and initially pristine contact spots and a homogenization of the SEI thickness. Each SEI patch is vertically (along the major growth direction) exposed to the same Δ*μ*_Li_. This leads to a laterally inhomogeneous *μ*_Li_ in SEI patches with different thickness and the buildup of lateral chemical potential gradients. These lateral chemical potential gradients drive lateral fluxes of lithium and will cause crosstalk. This process will become the path of least resistance when the SEI thickness difference (Δ*d*_SEI_ = |*d*_SEI,spot1_–*d*_SEI,spot2_|) between the two SEI spots becomes roughly equal to their lateral distance.

Importantly, although we did not simulate it here, we expect this change from isolated parallel growth, to homogenized growth driven by lateral crosstalk, to yield a resistance evolution remarkably similar to the saturation behavior discussed in Section 3.1.5 and Section 3.1.6: a rapid early resistance increase driven by the degradation of initially pristine and low resistive contact spots that saturates into an *apparent* saturation and slower growth regime. This late-stage regime is governed by current redistribution to prepassivated contact spots and subsequent homogenization of SEI thickness growth via crosstalk between the adjacent contact spots.

### 3.2 Experiments – Influence of Pressure on Impedance-Derived SEI Growth Kinetics

We now turn to the analysis of actual solid-state symmetric cells to investigate whether the insights gained in the simulation studies may be applicable to actual experimental data. The lithium|SE contact quality is affected by both, easily quantifiable parameters, such as applied stack-pressure, and less easily quantifiable variables, such as the atmospheric exposure history of the lithium foil during storage and handling.^58^ Mechanical pressure can be varied most systematically and is therefore mainly used here to assess the sensitivity of the *apparent* SEI growths trends to the experimental contact conditions.

To this end, Li|Li_6_PS_5_Cl|Li cells were prepared by isostatic joining at *P*_join_ = 20 MPa, 40 MPa, 75 MPa, and 350 MPa followed by impedance characterization at ambient pressure (1 atm, vacuum-sealed), *i.e*., without additional stack pressure during measurement (*P*_stack_ = 0 MPa). For each pressure in this measurement series, at least three cells were built. Cross-sectional FIB-SEM analysis of samples prepared at *P*_join_ ≥ 20 MPa revealed no visible differences in the “mechanical” contact area (see Figure 5a and Figure S4 of the Supporting Information). Consequently, we hypothesize that the major differences in the *apparent* SEI growth trends observed among these cells primarily originate from varying degrees of surface penetration by SE asperities into the native passivation layer on the lithium metal. Previous work by Otto *et al*.^58^ on Li|LLZO interfaces supports the assumption that higher joining pressures lead to an increased penetration depth and a larger number of SE surface asperities in direct contact with the alkali metal.

**Figure 5:**
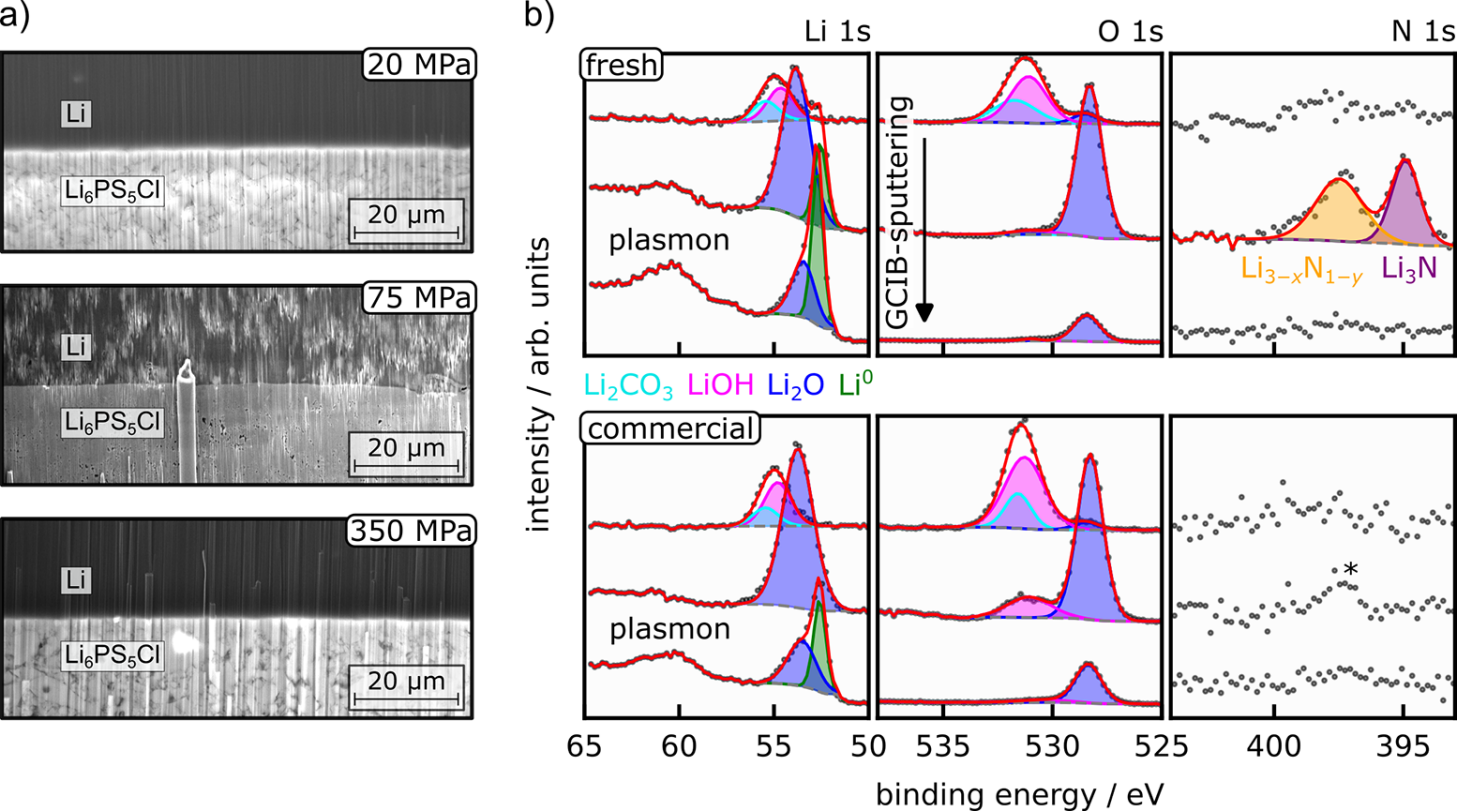
FIB-SEM Cross Sections of the Li|Li_6_PS_5_Cl Contact and XPS Analysis of Lithium Metal Foils. (a) FIB-SEM cross sections of the Li|Li_6_PS_5_Cl interface for cells isostatically joined at 20, 75, and 350 MPa. (b) XP spectra of the Li 1s, O 1s, and N 1s regions for a lithium foil freshly cut from a rod (top row: “fresh”) and of a commercial lithium foil (bottom row: “commercial”). Raw data are displayed by circles and overall fits as red lines. Fits for individual chemical components are represented by shaded areas (magenta: Li_2_CO_3_, pink: LiOH, blue: Li_2_O, green: Li^0^). Within each row, spectra show the depth evolution from the pristine surface (top) to after 2 min GCIB sputtering at 10 kV (middle) and 20 kV (bottom). XP spectra of a fresh lithium foil aged for 1 day in the same glovebox are provided in Figure S5.

The chemical composition of the native passivation layer on lithium metal is dictated by the cell assembly environment. Because the lithium metal electrodes were freshly cut from a rod in an argon-filled glovebox containing a relatively high partial pressure of nitrogen (*p*(N_2_)/*p* ≈ 50 ppm to 500 ppm) alongside trace amounts of oxygen and moisture (*p*(H_2_O)/*p* < 1 ppm, *p*(O_2_)/*p* < 1 ppm), the passivation layer is expected to consist primarily of Li_3_N, Li_2_O, LiOH, Li_2_CO_3_.^58,59^ Notably, Li_3_N has high ionic conductivity (σ_ion_ > 10^–4^ S·cm^–1^, α and β phase at 25 °C) and exceptionally low electronic conductivity, with reported values of σ_eon_ ≤ 10^–12^ S·cm^–1^.^61^ Thus, Li_3_N provides a significantly higher ionic and lower electronic conductivity compared to the SEI formed by Li_6_PS_5_Cl degradation (σ_ion_ ≈ 10^–7^ S·cm^–1^, σ_eon_ ≈ 10^–10^ S·cm^–1^).^39^ Importantly, in liquid type cells, intentionally created Li_3_N layers were already proven to improve the long-term stability of lithium metal anodes.^62^

Because the isostatic joining procedure could not be utilized below 20 MPa due to equipment limitations, spring-loaded press cells were employed for the pressure regime below 20 MPa. For these cells, uniaxial pressure had to be maintained throughout the measurement to preserve the electrical contact between the steel stamps and the lithium electrodes (see Section S7 and Figure S4 of the Supporting Information). Consequently, for the pressure regime from 1 to 20 MPa, the effects of contact formation during joining and those arising from stack pressure during the experiment cannot be decoupled. Regardless of the setup employed, impedance spectra were recorded over several days in a climate chamber at 25 °C with a frequency range between 3 MHz and 100 mHz.

#### 3.2.1 XPS Analysis of the Native Surface Passivation Layer on Lithium Metal Foil

Before discussing the impedance results, we first examine the native surface passivation on the lithium metal electrodes in more detail, since it likely affects the *apparent* SEI (impedance) growth trends. X-ray photoelectron spectroscopy (XPS) measurements were performed on lithium foils freshly prepared in the respective glovebox with *p*(N_2_)/*p* ≈ 300 ppm (*p*(H_2_O)/*p* < 1 ppm, *p*(O_2_)/*p* < 1 ppm, exposure time to the glovebox atmosphere ≈5 min). For comparison, additional XPS analyses were conducted on (*i*) freshly cut foils stored for 1 day in glovebox atmosphere and (*ii*) commercial lithium foils that feature an intentionally formed protective surface layer, primarily composed of Li_2_CO_3_ and LiOH, which were stored in the same glovebox for approximately one month. To obtain qualitative depth-resolved information, XPS spectra were collected both in the pristine state (*i.e*., without sputtering) and after two successive gas cluster ion beam (GCIB) sputtering steps. The resulting spectra for the freshly prepared and commercial foils are shown in Figure 5b (upper and lower row, respectively), while the spectra for the freshly cut and intentionally aged samples are provided in Figure S5 of the Supporting Information.

In the absence of sputtering, all samples exhibit convoluted signals in the Li 1s and O 1s regions, which can be attributed to Li_2_CO_3_ and LiOH (Li 1s: Li_2_CO_3_ ≈ 55.5 eV, LiOH ≈ 54.7 eV; O 1s: Li_2_CO_3_ ≈ 531.7 eV, LiOH ≈ 531.1 eV). These observations are consistent with prior reports^58,59,63^ and confirm that the outermost surface region is covered by a thin passivation layer rich in Li_2_CO_3_ and LiOH. With increasing sputtering depth, the relative intensity of the Li_2_CO_3_ and LiOH signals progressively decreases, indicating that these species are primarily confined to the outermost surface region. Simultaneously, the intensity of the Li_2_O signal (Li 1s ≈ 53.8 eV; O 1s ≈ 528.3 eV) and the signal assigned to lithium metal (Li^0^, Li 1s ≈ 52.7 eV and plasmon-loss ≈ 60 eV) increases.^63^ Importantly, the presence of Li_2_O can also be due to the reaction of metallic lithium with background gas residues in the vacuum chamber or due to sputter induced decomposition of Li_2_CO_3_ and LiOH.^59,63^ However, we expect GCIB-sputtering to be less detrimental compared to Ar^+^-ion sputtering. In addition to the oxygen related signals, for the freshly prepared foil, new spectral features emerge in the N 1s region that we assign to Li_3_N (N 1s: 394.9 eV) and a lithium–nitrogen compound Li_3–*x*_N_1–*y*_ (N 1s: 397.5 eV).^58,63^

This depth-dependent evolution of the XP spectra suggests a layered passivation structure, in which a thin, protective Li_2_CO_3_/LiOH layer overlays a subsurface region enriched in Li_3_N and likely other inorganic species such as Li_2_O. Most significantly, no clear Li_3_N-related signal is observed for the commercial foil. At most, a weak and ambiguous feature (marked with *) appears near the binding energy assigned to Li_3–*x*_N_1–*y*_, but its low intensity precludes an unambiguous assignment. For the freshly prepared foils, Li_3_N-related signals become more prominent already after the first sputtering step, yet they remain low in intensity, indicating the presence of a comparatively thin passivation layer. On the other hand, for the freshly cut but intentionally aged foil, it takes three more sputtering steps to obtain signals related to Li_3_N, exhibiting a significantly higher intensity than the nonaged freshly prepared foil (see Figure S5 of the Supporting Information).

Taken together, the XPS results provide evidence for the presence of a thin Li_3_N layer on the lithium metal surface. The layer seems to be covered by a layer of Li_2_CO_3_ and LiOH. Depending on the applied pressure, SE asperities may locally penetrate the outer Li_2_CO_3_/LiOH layer during joining and establish contact with the underlying Li_3_N-rich region and the metallic lithium. Given the favorable transport properties of Li_3_N, such localized contact points may exhibit enhanced ionic transport properties while maintaining low electronic leakage.

#### 3.2.2 Impedance Evolution and Resistance Growth Trends

Figure 6 summarizes the experimental impedance results for the cells investigated and prepared using varying pressure conditions. All impedance data were normalized to the *apparent* electrode area and multiplied by a factor of 0.5 to account for the two Li|SE-interfaces. Representative initial impedance spectra recorded after cell assembly are presented in Figure 6a. In analogy to the simulation study, the bulk resistance *R*_bulk_ (including grain boundaries) is subtracted from the real part of the impedance *Z* ^′^ to focus on the evolution of features typically assigned to interfacial processes.

**Figure 6:**
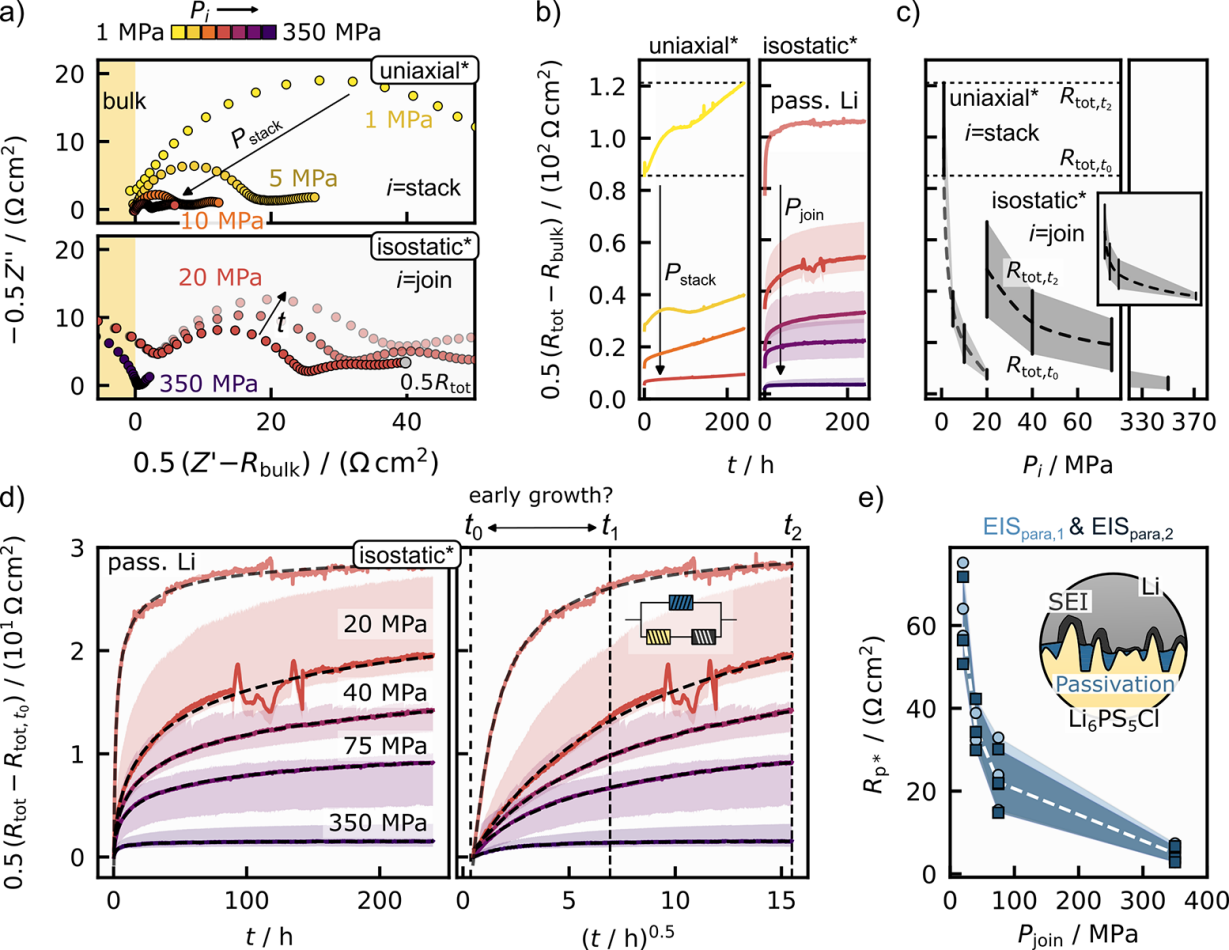
Influence of joining- and stack-pressure on the impedance evolution of symmetric Li|Li_6_PS_5_Cl|Li cells and *apparent* interphase growth trends. (a) Comparison of the initial impedance spectra (i.e., at *t*_0_) at different applied stack pressures (first row: uniaxial*, 10 MPa-20 MPa) and isostatic joining pressure (second row: isostatic*, 20 MPa and 350 MPa). The impedance increases with time, as exemplarily shown for a cell isostatically pressed at 20 MPa (second row, arrow with t ). (b) Temporal resistance evolution for different uniaxial stack pressures (left column: top down: 1 MPa, 5 MPa, 10 MPa, and 20 MPa) and isostatic joining pressures (right column: top down: 20 MPa, 40 MPa, 75 MPa, 350 MPa). The impedance value at the lowest frequency is used as a proxy for the total resistance *R*_tot_ of the respective cell (see gray dot in (a) 0.5 *R*_tot_). The shaded areas illustrate the corresponding data spread of at least three cells, whereas the solid lines represent the resistance evolution of the cell that lies closest to the respective average growth curve. The line labeled as “pass. Li” shows the resistance evolution of a cell isostatically joined at 20 MPa with deliberately degraded lithium foil exposed to laboratory atmosphere. (c) Pressure dependence of the initial resistance $R_{\mathrm{tot},t_{0}}$ and final resistance $R_{\mathrm{tot},t_{2}}$ (*t*_0_ = 0 h, *t*_2_ = 240 h) of the measurement for the uniaxial cells (*i* = uniaxial*) and the isostatic cells (*i* = isostatic*). The dashed lines and shaded areas are drawn as a guide to the eye. The small inset enlarges the resistance trend for the isostatic cells. (d) Fitted resistance trends (black, dashed lines) after subtraction of $R_{\mathrm{tot},t_{0}}$ . The data was fitted with the parallel equivalent circuit model (from Figure 4) displayed in the right column of the square-root-of-time plot. (e) Passivation path resistance $R_{p*}$ fitted with the parallel model from (d) (EIS_para,2_) and a simplified version of it, without constriction resistance (EIS_para,1_). The shaded regions illustrate the data spread. The dashed line linearly connects the average value at each pressure. The inset illustrates the proposed contact scenario. More details are provided in the main text.

The upper graph of Figure 6a (denoted “uniaxial*”) illustrates the pressure dependence of the initial impedance for cells measured under continuous stack pressures ranging from 1 MPa to 20 MPa. The lower graph (denoted “isostatic*”) displays the initial spectra of cells joined isostatically at 20 MPa, and 350 MPa, but characterized without applying stack pressure (*P*_stack_ = 0 MPa). Across both cell setups, a systematic dependence on the applied pressure is clearly visible: increasing the uniaxial stack pressure or the isostatic joining pressure leads to a significant decrease in the overall impedance, in line with a deeper penetration of SE surface asperities into the native passivation layer on the lithium metal foil. Furthermore, the impedance of all evaluated cells increases over time, as exemplarily shown for a cell isostatically joined at 20 MPa (lower graph).

Providing a detailed assignment of the evolving impedance features to specific physical processes remains challenging. As discussed in Section 3.1.4, when multiple parallel-connected phases (such as the SEI and native surface passivation) are present at the interface, assigning specific impedance signals to individual components is rarely feasible. Specific impedance features can originate from localized conductivity relaxations of distinct phases or transitions that shift current pathways entirely.^50^ Nevertheless, in Section S9 of the Supporting Information, we provide a more detailed discussion of the impedance data, including distribution of relaxation time (DRT) analysis.

To analyze the impact of SEI formation consistently, we adopt the approach used in the simulation sections and monitor (*R*_tot_–*R*_bulk_) over *t*. As a proxy for *R*_tot_, we extract *Z* ^′^ from the lowest frequency point of each spectrum (0.1 Hz), as indicated in Figure 6a (data point highlighted in gray). While this approach considers only resistive contributions that occur at characteristic frequencies ≥0.1 Hz, it remains the simplest method for handling the mixed origin of the impedance signals. It must be noted, however, that the resulting resistance evolution represents a lower bound for the underlying total resistance development. Extending the frequency range further down, *e.g*., into the mHz regime, to capture subsequent impedance features is practically precluded during early SEI growth. The time required for such low-frequency sweeps violates the stationarity requirement. Moreover, as we will discuss in more detail in Section 3.3, the extended polarization time would result in a significant amount of lithium being electroplated and dissolved, making the measurement more invasive. Furthermore, the primary conductivity relaxation of the SEI is expected to lie above 0.1 Hz.

The extracted (*R*_tot_–*R*_bulk_) values are presented in Figure 6b as a function of measurement time *t*, for both the uniaxial cells (left panel, 1 MPa–20 MPa) and the isostatic cells (right panel, 20 MPa–350 MPa). Notably, the uniaxial cells exhibit distinct regions of saturation or even sudden decreases in (*R*_tot_–*R*_bulk_). The exact cause of these features, which are particularly pronounced under low stack pressures, remains unclear. One hypothesis is the mechanical or morphological failure of the SEI (*e.g*., the collapse of fragile contact points). However, at these low stack pressures, we cannot rule out that friction between the steel stamps and the rubber O-rings in press cells causes or influences these features. Therefore, for the remainder of the analysis, we focus mainly on the cells monitored without stack pressure, *i.e*., those joined isostatically at higher pressure (right panel).

For these isostatic cells, the (*R*_tot_–*R*_bulk_) vs *t* curves reveal a significant deviation from a square-root-of-time dependence, that is predicted by the Wagner model. Neither does the data reflect a Deal-Grove-type trend (compare with Figure S3 of the Supporting Information); Initially, the resistance increases rapidly before transitioning into an *apparent* saturation. While this contrasts with reports by Wenzel *et al*.^19^ (impedance) and Aktekin *et al*.^20^ (CTTA), it closely aligns with observations made by Riegger *et al*.^32^ for Li|Li_6_PS_5_Cl|Li cells investigated using impedance spectroscopy. Moreover, the (*R*_tot_–*R*_bulk_) vs *t* trend mirrors the simulation results from Section 3.1.4 for interfaces with partially penetrated passivation layers with negligible electronic conductivity; During the later stages of SEI growth, the Li_3_N containing, native passivation layer on the lithium foil may provide alternative, low-resistance ionic current pathways that are protected against SEI formation by low electronic conductivity. By increasing pressure, the initial resistive offset $R_{\mathrm{tot},t_{0}}$ and the final resistance $R_{\mathrm{tot},t_{2}}$ decrease (see Figure 6b and Figure 6c). Higher joining pressure likely forces the SE surface asperities deeper into the passivation layer,^58^ leading to an overall reduced resistance.

To highlight the role of the native passivation layer, a cell was fabricated using a deliberately degraded lithium foil exposed to laboratory air for approximately 10 s prior to joining at 20 MPa. The visually darkened foil yields the expected resistance trend: a large initial resistance $R_{\mathrm{tot},t_{0}}$ , a steep initial resistance growth, and a significantly higher final resistance plateau (“pass. Li”). For a thick surface passivation, only a tiny fraction of SE surface asperities may reach the reactive metal and the average passivation thickness is certainly increased, causing early rapid, geometrically magnified resistance growth that enters into a higher, stable final resistance (compare to Figure S2 of the Supporting Information)

To quantify our observations, the (*R*_tot_–*R*_bulk_) versus *t* curves were fitted using the equivalent circuit models proposed in our simulation study. The parallel model from Section 3.1.4—accounting for simultaneous conduction through the SEI and the passivation layer (with its associated constriction resistance)—fits the data with high accuracy. The corresponding fitted data is presented in Figure 6d, after subtraction of the initial resistance $R_{\mathrm{tot},t_{0}}$ for visualization purposes. Note that this model assumes a negligible electronic conductivity of the passivation layer. Conversely, a classical Wagner (series) model fails to capture the complete growth trend and only describes the trends reasonably well when restricted to the late stages of the experiment (*t* > 48 h, *t*_1_ to *t*_2_ in Figure 6d). Obviously, the choice of the fitting window in case of the series type model is quite arbitrary and yields different results depending on the specific constraints applied. The fitting procedure, model equations, fit parameters, and exemplary fit and residual curves (including cells investigated with stack-pressure) are provided and discussed in more detail in Sections S11, S17, and S18 of the Supporting Information.

The extracted passivation path resistances, *R*_p*_, as a function of joining pressure, *P*_join_, are displayed in Figure 6e. They reflect the resistance plateau to which the growth curves converge. Both parallel models yield quite similar results and show a consistent decrease of *R*_p*_ with increasing pressure, in line with a deeper penetration of SE asperities into the native passivation layer. The proposed contact scenario is illustrated in Figure 6e.

#### 3.2.3 Apparent SEI Rate Constants and Comparison with CTTA

The *apparent* rate constants extracted using the parallel and series models are depicted in Figure 7 (bright blue left-pointing triangles: EIS_para,1_, dark blue right-pointing triangles: EIS_para,2_, gray circles: EIS_series,late_). For comparison, we include rate constants derived from impedance spectroscopy in the literature by Wenzel *et al*.^19^ (green downward-pointing triangle) and Riegger *et al*.^32^ (orange upward-pointing triangles), alongside constants obtained via CTTA from both our own experiments (dark red squares) and the literature (Sivavec *et al*.^34^). In the case of CTTA, the native passivation layer found on lithium should play no significant role, since the method relies on freshly electroplated lithium, like “anode-free” cells. It therefore serves here, in our opinion, as a reliable benchmark for the actual growth kinetics.

**Figure 7:**
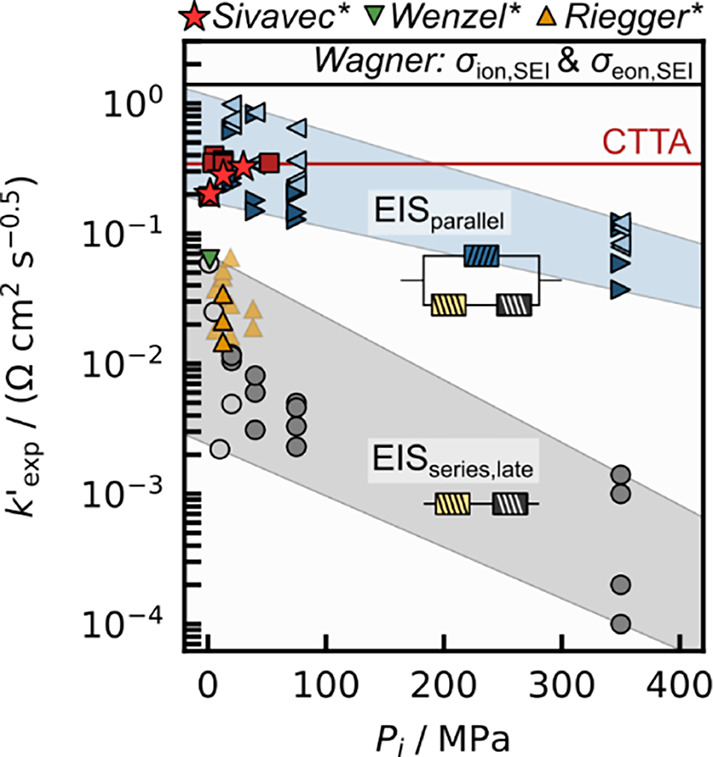
Overview of the influence of pressure on *apparent* rate constants obtained by impedance spectroscopy and CTTA. Data points obtained from our CTTA experiments are shown as dark red squares. Impedance-derived rate constants from our work are divided by fitting methodology: values extracted using the parallel models (bright blue left-pointing triangles: EIS_para,1_, dark blue right-pointing triangles: EIS_para,2_) capture the early stage *apparent* growth kinetics of the resistance growth curve, while values extracted using the series model (gray circles) capture the late saturating region of the resistance curve. Literature data are included for comparison: Wenzel *et al*.^19^ (green downward-pointing triangle, EIS), Riegger *et al*.^32^ (orange upward-pointing triangles, EIS; solid for freshly cut lithium, transparent for commercial lithium), and Sivavec *et al*.^34^ (red stars, CTTA). The solid black line at the top represents the theoretical rate constant predicted from the partial ionic and electronic conductivities of the SEI reported by Alt *et al*.^39^ The shaded areas are drawn as a guide to the eye. The red, solid line marks the rate constant to which the CTTA measurements seem to converge.

Crucially, for all investigated CTTA cells, the evolution of the cumulative charge over time exhibited a trend consistent with a square-root-of-time dependence (see Figure S13 of the Supporting Information), supporting a diffusion-controlled SEI growth mechanism.^36^ Although we observed a slight pressure dependence by increasing the pressure from 1 MPa to 4 MPa, a further increase did not yield significantly different results. Consequently, while these findings qualitatively corroborate the observations of Sivavec *et al*.,^34^ the pressure dependence in our system is notably less pronounced.

To approximate the *apparent* resistance growth rate constants from CTTA measurements, we estimated the rate constant, *k*_exp_, for *apparent* SEI thickness growth according to the procedure used by Aktekin *et al*.^20^ The corresponding rate constant for resistance growth, $k_{\exp}^{\prime }$ , was then estimated based on the ionic conductivity of the SEI determined by Alt *et al*.^39^ The rate constants from the data of Sivavec *et al*.^34^ were estimated from their cumulative charge curves using the same approach (see Section S12 of the Supporting Information for more details). It should be noted that this calculation presupposes the deposition of a homogeneous lithium layer during CTTA, which then degrades into a uniform and dense SEI. Consequently, the derived values should be treated as order-of-magnitude estimates.

The slight pressure dependence in CTTA can be understood by considering an increase in the *true* contact area between the stainless steel and the Li_6_PS_5_Cl SE with increasing pressure. On stainless steel, lithium is not plated as a dense, uniform thin film, but rather as an ensemble of discrete nuclei. Because the measured OCV is a *global* property, it remains at ∼0 V vs Li^+^/Li until the very last lithium nucleus is fully consumed. The nuclei spots do not degrade independently due to their shared electronic connection to the stainless-steel current collector and ionic connection to the SE. Electrons from nuclei at thick SEI spots can bypass the high electronic resistance of their *local* SEI by traveling through the highly electron conductive stainless-steel foil. Simultaneously, lithium ions may diffuse through the *local* SEI into the bulk solid electrolyte to meet these electrons at steel|SE contact points with thinner SEI, forming new SEI at these spots remotely. An improved stainless steel|SE contact area provides more sites for this electrochemical crosstalk to occur, which leads to an increased *apparent* rate constant for lithium consumption. Consequently, the *apparent* rate constants derived from CTTA and impedance spectroscopy exhibit opposing trends: in CTTA, the *apparent* rate constant *increases* with pressure, whereas in impedance, it *decreases* with pressure as shown in Figure 7, both driven by an improvement in the interfacial contact area.

The *apparent* rate constants obtained from the resistance evolution using the parallel models are approximately one order of magnitude larger than those obtained via the series model. According to the model (Section 3.1.4), these rate constants reflect resistance growth induced by the degradation of the initially most pristine contact spots that penetrate the native surface passivation layer. Theoretically, these rates should serve as an upper bound for the *intrinsic* rate constant, since the area of these initially pristine contact spots is likely only a fraction of the total *apparent* contact area.

Conversely, the physical interpretation of the parameters obtained via the series models is inherently more arbitrary in this context, as their values depend significantly on the specific time window utilized for data fitting. The *apparent* resistance saturation captured by these series fits is, in our view, not representative of *intrinsic* SEI growth kinetics. Rather, it likely reflects a dynamic shift in the preferred ionic transport pathways across the heterogeneous interface or in general, an *effective* parameter affected by the presence of the native passivation layer on the metal foil: as the initially most pristine contact spots rapidly degrade and become highly resistive, the ionic current redistributes toward regions that are kinetically protected against SEI formation by the native surface passivation film, as previously discussed in Section 3.1.4 of our simulation study. Homogenization of SEI thickness growth as discussed in Section 3.1.6 might also play a role here.

Overall, the *apparent* rate constants obtained by impedance spectroscopy are smaller compared to CTTA. The values obtained with the parallel models come closer to the rate constants estimated by CTTA but still deviate significantly. Moreover, both the CTTA and impedance estimates yield smaller *apparent* rate constants for resistance growth than theoretical predictions based on the partial ionic (σ_ion,SEI_) and electronic (σ_eon,SEI_) conductivities determined for the SEI by Alt *et al*. (black line, “Wagner”). As these rely on all assumptions of the pure Wagner diffusion model, these can be considered an upper theoretical limit. Finally, the literature data obtained by Wenzel *et al*. and Riegger *et al*. fit seamlessly into the pressure-dependent trend established by our series-model analysis. Although their data analysis methodologies slightly deviate from the one applied in this work, they rely on a similar series-type equivalent circuit fitting of the impedance evolution for symmetric Li|Li_6_PS_5_Cl|Li cells.

The deviation between the *apparent* rate constants derived from EIS, CTTA, and the theoretical predictions highlights the inherent limitations of extracting interlaboratory comparable and reproducible *intrinsic* kinetic parameters. Rather, these rate constants represent *effective* parameters governed by the experimental contact conditions provided, including the atmospheric exposure history of the metal foil. Interestingly, based purely on the geometric considerations of our simplified contact model (*e.g*., a single pristine contact spot surrounded by an electronically insulating passivation layer), one might expect the parallel EIS models to systematically overestimate the *intrinsic* rate constant. Because the *true* contact area of these pristine contact spots is smaller than the *apparent* contact area, localized SEI formation at these spots induces a disproportionately large resistance increase. This geometrically magnified resistance increase inflates the *apparent* rate constant (see Figure 4 in Section 3.1.4: $k_{\exp}^{\prime }$ = $A_{r}^{-1}k^{\prime }$ , where *A*_r_ is the relative areal coverage with initially pristine contact spots).

However, our experimental data show the exact opposite trend: the EIS analysis underestimates the rate compared to CTTA. This discrepancy likely arises because the actual contact scenario is vastly more complex than our idealized models propose. Real interfaces possess a broad statistical distribution of asperity penetration depths, multiphase native passivation layers, and non-negligible residual electronic conductivity within those passivating films. Thus, while our simplified models successfully capture the qualitative trends observed for the resistance evolution—such as resistance saturation in the presence of a passivation layer, as well as different slopes—they cannot fully describe the scenario quantitatively. In reality, the SE surface asperities may fail to completely penetrate through the native passivation layer. Given this layer’s potential low electronic and high ionic conductivity, SEI growth may genuinely decelerate compared to CTTA measurements, which rely on freshly deposited lithium. Thus, our observations likely convolute two effects: an *actual* deceleration of SEI growth due to kinetic hindrance by the native passivation layer, and an *apparent*, saturation driven by current redistribution and thickness homogenization.

### 3.3 Shortcomings, Uncertainties, and Directions for Future Work

Our analysis should be understood as a *simplified*, yet *physically consistent*, *qualitative* description of the phenomena governing the observed resistance trends. The underlying model assumptions necessarily represent abstractions of complex interfacial processes. In practice, a range of additional, interrelated factors may influence resistance evolution, including surface roughness, the processing-induced microstructure of the alkali-metal electrodes, and the *local* microstructure of the solid electrolyte.

The SEI formed between lithium and Li_6_PS_5_Cl is a composite consisting of several decomposition products, likely primarily Li_2_S, Li_3_P, and LiCl, which possess vastly different ionic and electronic conductivities.^19^ Notably, the electronic conductivity of Li_3_P exceeds that of Li_2_S and LiCl by more than 6 orders of magnitude.^64^ As a consequence, the effective transport properties of the interphase may evolve significantly during the early stages of growth. Therefore, alternative mechanisms contributing to the observed resistance trends cannot be excluded.

Experimental limitations further compound these constraints. In our EIS measurements, there is a temporal delay (up to 1 h) between cell assembly and the acquisition of the first complete impedance spectrum. For a heterogeneous interface, rapid SEI growth at the deepest, most pristine contact spots may already been completed during this unmonitored window. Consequently, what we fit as “early-stage” SEI growth may already represent the onset of saturation. At this time, the ionic current is shifting into the native passivation layer due to high *local* resistance at the primary contact spots.

A critical assumption that must be challenged in future studies is the noninvasive nature of the EIS measurement itself, particularly at low frequencies. In Section S15 of the Supporting Information, we estimate the amount of lithium metal being stripped and plated in a half-cycle at different frequencies, assuming homogeneous plating and stripping. For an excitation voltage of 10 mV, 100 mHz corresponds to ≈0.6 nm (1–2 atomic layers), whereas 10 mHz, 1 mHz, and 0.1 mHz correspond to 6 nm, 60 nm, and 600 nm, respectively. For one cell where measurements were extended down to 1 mHz, the low-frequency portion of the spectrum (below approximately 1 Hz) is highly nonlinear and strongly dependent on the applied excitation voltage (tested from 0.5 mV to 20 mV; see Figure S9 in Section S10 of the Supporting Information). We hypothesize that this nonlinear feature originates from electroplating, dissolution, and electrocrystallization at the highly heterogeneous interfaces.

Because these nonlinear processes of unclear origin are (partially) excluded by our 0.1 Hz cutoff, the resistance evolution we extracted must be taken as a lower bound. Importantly, this methodological constraint may partially explain why the apparent rate constants extracted from our impedance data are systematically smaller compared to those extracted from CTTA. Future work should therefore prioritize a more detailed understanding of the impedance data of such cells and specifically the origin of the low-frequency impedance feature.

In addition, combining impedance spectroscopy with direct-current techniques may provide deeper insight into the practical relevance of SEI growth in solid-state batteries. Preliminary results presented in Section S13 of the Supporting Information already indicate that *apparent* SEI resistance growth scales with the amount of stripped lithium, consistent with a decreasing *true* contact area. Furthermore, performing EIS on cells with electroplated lithium may help to decouple *intrinsic* from *apparent* growth kinetics.

In Table S1 of the Supporting Information, we contextualize our findings against prior, relevant literature on SEI growth at the sulfide solid electrolyte|lithium metal interface. This table outlines the historical progression of the field, providing a comprehensive overview of some relevant studies where EIS or CTTA have been applied.

## 4 Conclusions

The central message of our analysis is twofold. First, theoretical modeling of SEI growth based on transport theory (*e.g*., the Wagner model as demonstrated by Alt *et al.*), establishes an upper limit for *intrinsic* SEI rate constants. These rate constants represent a “worst-case” scenario of the fastest possible SEI formation within a cell. Second, *apparent* rate constants determined by EIS or CTTA seem to underestimate these *intrinsic* rate constants that are governed solely by the *intrinsic* transport properties of the SEI itself. In our EIS measurements, this discrepancy is likely driven by the presence of a native surface passivation layer on the lithium metal foils, which kinetically hinders SEI growth. Competing transport pathways (*e.g*., through both SEI and a native passivation layer on reactive alkali metal electrodes) can lead to an *apparent* saturation in cell resistance that does not *necessarily* reflect a *true* saturation of the interphase growth.

Consequently, the choice of measurement conditions must be guided by the intended purpose of the investigation. Investigating *intrinsic* SEI growth kinetics requires pristine surfaces and intimate contact. In contrast, if the objective is to understand how SEI growth affects overall cell resistance under certain conditions, these specific conditions should be adopted. Anyhow, in this case, extracted growth trends do not reflect *intrinsic* kinetics of interphase formation. To ensure cross-study comparability, detailed reporting of applied pressure, and atmospheric exposure history is essential.

Finally, we emphasize the critical need to resolve remaining knowledge gaps in the impedance data analysis of symmetrical Li|Li_6_PS_5_Cl|Li cells, particularly in the low-frequency regime. A complete understanding of the impedance features is a strict prerequisite for ensuring accurate interpretation of extracted resistance trends and SEI growth mechanisms, and should be a primary focus of future studies.

## 5 Computational Details

### 5.1 Generation of Three-Dimensional Microstructures

The geometrical model systems with all their geometrical parameters used are presented at the beginning of each simulation series in the Results and Discussion section. The icons in Figures 2–4 show the considered experimental system in comparison to the corresponding computational model system. All microstructures were built using Numpy.^65^

### 5.2 Transport Description and Choice of Material Parameters

All transport computations are based on a microstructure-resolved 3D network model that describes ionic transport. The modeling approach is illustrated in Figure 1 for an ideal interface contact. To this end, the model system is discretized, and transport is described between the individual voxels. The *local* transport properties depend on the phase assignment of each voxel. Bulk and grain boundary transport in different materials (*i.e*., SE, interlayer, electrode) are represented by *RC*-elements, while pores are modeled as capacitors. Transport across an interphase is considered as an additional *RC*-element between two voxels of different materials. The *local* transport parameters of these processes are considered constant (*i.e*., time-independent) within a simulation series. Low-frequency diffusion processes are not considered in the simulations, and the electronic conductivity of the solid electrolyte and the interphase is assumed to be negligible.

For the time-dependence of the *local* SEI parameters (*R*_SEI_, *C*_SEI_), we assume diffusion-controlled interphase growth following (eq 3). This implies that interphase growth at each interface element remains quasi-1D and is not affected by its neighboring elements. Since the interphase is significantly thinner than the voxel resolution of our simulation, it is not explicitly resolved as a structural domain. This means that charge transport through the interphase is only considered in the direction perpendicular to the electrode interface, with no lateral transport within the interphase plane. This is similar to the assumption of the Wagner diffusion model presented in Section 2.1, and an acceptable simplification given the thin nature of the interphase and the significantly lower ionic conductivity compared to the bulk solid electrolyte.

The *local* circuit parameters in the electric network are determined using the standard formulas for resistors and plate capacitors. The material-specific transport parameters for the metal and SE are based on literature data.^66,67^ Unless stated otherwise, the following parameters are used: (i) The permittivity of the pores is set to vacuum permittivity, *i.e*., *ε*_pore_ = *ε*_0_. (ii) The parameters for Li_6_PS_5_Cl are set to *σ*_SE_ = 2.0 mS·cm^–1^, and *ε*_SE_ = 20 *ε*_0_. The partial conductivity of the forming SEI at the Li|Li_6_PS_5_Cl interface is set to *σ*_ion_ = 134 nS·cm^–1^ and *σ*_el_ = 0.3 nS·cm^–1^, based on recent literature values.^39^ In Section S14 of the Supporting Information, we test the truncation error using a model system with a single square-shaped pore. The results show that small quantitative variations <10% occur with changes in grid resolution. As our simulations aim to capture qualitative trends based on simplified model geometries, we consider these differences acceptable. Based on Δ*μ*_Li_ (= 158.7 kJ·mol^–1^), and the SEI partial electronic and ionic conductivities determined by Alt *et al*.^39^ (*σ*_ion,SEI_ = 1.34·10^–7^ S·cm^–1^ and *σ*_eon,SEI_ = 0.3·10^–9^ S·cm^–1^), we determine a rate constant for the areal resistance growth of $k_{\mathrm{ref}}^{\prime }=1.4\;{\Omega \cdot \mathrm{cm}}^{2}\cdot s^{-0.5}$ .

### 5.3 Experimental Details

Preparation of materials and cell assembly were carried out in an argon-filled glovebox (*LabMasterPRO*, MBraun, Garching, Germany), with *p*(O_2_)/*p* and *p*(H_2_O)/*p* < 1 ppm. The *p*(N_2_)/*p* varied between ≈ 50 ppm and 500 ppm.

### 5.4 Preparation of Materials

Commercial Li_6_PS_5_Cl powder (POSCO Chemical Co, Korea) with a particle size of 5 μm was used as separator. Lithium metal electrodes were prepared by cutting lithium metal (99.8% purity, MaTecK Material-Technologie & Kristalle GmbH, Jülich, Germany) freshly from a rod. The lithium was subsequently pressed manually between two pouch foils to achieve a thickness of ∼100 μm. Circular electrodes (*d* = 9.6 mm for press cells, *d* = 6 mm for pouch cells) were punched out from these foils. A steel foil (AISI 304 steel, *d* = 9.6 mm) with a thickness of 20 μm was used as a current collector.

### 5.5 Cell Assembly

Cells were assembled using press cell casings with a 10 mm polyetheretherketone (PEEK) mantle and tested under stack pressures ranging from 1 MPa to 20 MPa. To prepare the SE separator, 90 mg of Li_6_PS_5_Cl powder were uniaxially pressed between stainless-steel stamps at 380 MPa for 3 min. Afterward, a hand-pressed lithium metal foil and a stainless-steel foil were placed on each side of the compacted pellet. The cell was then mounted into a pressure frame equipped with a metal spring and force sensor. Stack pressures of 1 MPa, 5 MPa, 10 MPa, and 20 MPa were applied.

Pouch cells were prepared using a similar setup. First, Li_6_PS_5_Cl pellets (90 mg) were precompacted at 400 MPa in an isostatic press. Then, the assembled cells consisting of lithium metal and stainless-steel foils on both sides were again isostatically pressed at 20 MPa, 40 MPa, 75 MPa, and 350 MPa. Finally, the cells were vacuum-sealed in bags with nickel current collector tabs.

### 5.6 FIB-SEM

Cross sections were prepared by FIB-SEM using a XEIA3 system (TESCAN GmbH, Czech Republic). The samples were transferred from an argon filled glovebox to the XEIA3 system under inert gas conditions using a LeicaEM VCT500 transfer shuttle (Leica Microsystems GmbH, Germany). Samples were milled and imaged under cryo-conditions (liquid nitrogen cooling). Cross sections were milled with Xe^+^ ions with an energy of 30 kV. Beam currents of 2400 nA, 410 nA, and 140 nA were used for milling and polishing. The cross sections were imaged using a beam current of 1600 pA, an acceleration voltage of 15 kV, and a secondary electron detector.

### 5.7 XPS

The XPS measurements on lithium foils (commercial lithium foil: 100 μm thickness, 99.9% purity, China Energy Lithium, China; lithium foils cut from a rod: ≈100 μm, 99.8% purity, MaTecK Material-Technologie & Kristalle GmbH, Jülich, Germany) were carried out using a Versa Probe IV system (Physical Electronics GmbH, Germany). Monochromated Al–Kα radiation (1487.6 eV, 15 kV, 200 μm beam diameter and 50 W power) was used in the measurements (with dual beam charge compensation). For depth profiling, Ar-ion clusters with acceleration voltages of 10 kV and 20 kV were used for sputtering over an area of 1 mm^2^, while Ar^+^ -ions with an acceleration voltage of 4 kV were used over an area of 4 mm^2^. A pass energy of 224 eV with 0.8 eV steps was used for overview spectra and a pass energy of 55 eV with 0.2 eV steps was used for the detail spectra. All samples were fixed with insulating double-sided adhesive tape. The sample preparation was performed in an argon-filled glovebox with *p*(O_2_)/*p* < 1.0 ppm and *p*(H_2_O)/*p* < 1.0 ppm. All samples were transferred to the XPS machine in an airtight transfer vessel. Data evaluation was carried out with the software CasaXPS (version 2.3.26, Casa Software Ltd.). All data were calibrated in relation to the signal of adventitious carbon at 284.8 eV. A Shirley background was used, and all spectra were fitted with a GL line shape.

### 5.8 Electrochemical Measurements

Electrochemical characterization was performed in a climate chamber at 25 °C using VMP3 or VMP300 potentiostats (BioLogic, France). Press cells were investigated while applying the respective stack pressure. Electrochemical measurements of pouch cells were performed at atmospheric pressure in vacuum sealed pouch bags, *i.e*., without applying stack pressure. All cells were investigated within 1 h after assembly.

Potentiostatic electrochemical impedance spectroscopy (PEIS) was performed in a frequency range between 3 MHz and 100 mHz with a voltage amplitude of 10 mV. After each PEIS measurement, the open-circuit voltage was measured for 2 min. The procedure was repeated for several days until the experiment was completed. To check if the electrochemical protocol itself has an impact on the measurement outcome, for selected Li|Li_6_PS_5_Cl|Li cells, the measurement protocol was interrupted and afterward started again (see Figure S18 and Figure S19).

Coulometric Titration Time Analysis (CTTA) tests were performed while applying the respective pressure in a climate chamber at 25 °C using a MACCOR battery cycling workstation. Stainless steel discs (with 9.6 mm diameter) punched from 20 μm-thick foils were used as working electrode and current collector in press cell setups. A high-precision punch (Nogamigiken Co., Ltd.) was used to ensure punching of flat and deformation-free planar discs. As counter and reference electrode, lithium discs with 9 mm diameter were used. The lithium plating current was 10 μA and the charge was limited to 1 μAh in each step. During OCV periods, the upper voltage limit was 0.05 V.

## Supplementary Material



## Data Availability

The data that support the findings of the study are available from the corresponding author upon reasonable request.

## Notes

S1: derivation of the deal-grove model for interphase growth on pre-passivated interfaces; S2: derivation of Δ*μ*_Li_ of the decomposition reaction at the Li|Li_6_PS_5_Cl interface; S3: the influence of the spatial contact distribution; S4: influence of native, partially penetrated surface passivation – surface coverage; S5: transition time estimation; S6: the influence of passivation layer electronic conductivity on SEI resistance growth; S7: FIB-SEM cross sections after joining; S8: XPS data of a lithium foil cut from a rod and aged in glovebox atmosphere; S9: detailed impedance data and distribution of relaxation time; S10: low-frequency impedance and excitation-voltage dependence; S11: model and parameter overview; S12: CTTA measurements; S13: apparent interphase growth kinetics after electro-dissolution and plating; S14: truncation error; S15: amount of lithium metal being electroplated and dissolved during EIS; S16: summary table and literature overview; S17: parameter tables; and S18: resistance curves, fits, and residuals (PDF)
