# Supplementary material for: SEI Formation in Sulfide-Based Solid-State Batteries: Influence of Contact Conditions on Impedance-Derived Interphase Growth Kinetics
Source: ACS Appl Mater Interfaces. 2026 Jun 4;18(23):33450–68. doi: 10.1021/acsami.6c07844 (PMC13288402; doi:10.1021/acsami.6c07844)
Supplement: Supplementary file 1 [file am6c07844_si_001.pdf]

Supporting Information

SEI Formation in Sulfide-Based Solid-State Batteries: Influence of Contact Conditions on Impedance-Derived Interphase Growth Kinetics

Sascha Kremer[1,2⊥], Christoph D. Alt[1,2⊥], Luca Schuster[1,2], Johannes Westphal[1,2], Burak Aktekin[1,2], Jürgen Janek[1,2], and Janis K. Eckhardt[2*]

[1]Institute of Physical Chemistry, Justus-Liebig-University Giessen, Heinrich-Buff-Ring 17, Giessen D-35392, Germany.
[2]Center for Materials Research (ZfM), Justus-Liebig-University Giessen, Heinrich-Buff-Ring 16, D-35392 Giessen, Germany.

⊥ S.K. and C.D.A. contributed equally to this work.

*janis.k.eckhardt@theo.physik.uni-giessen.de

S1. Derivation of the Deal-Grove Model for Interphase Growth on Pre-Passivated Interfaces[1]

The core assumption of the Wagner-[2] and the Deal-Grove[1] model is that the rate at which a degradation layer grows is proportional to the thermodynamic driving force and inversely proportional to the resistance blocking the necessary transport through the degradation layer itself (in this case, ambipolar transport of ions and electrons). Because the new SEI forms under an existing passivation layer, the reactive species must travel through both layers in series. Therefore, the total ambipolar area-specific resistance $R_{\mathrm{amb,tot}}$ is the sum of the (areal) ambipolar resistances of both layers:

$$R_{\mathrm{amb,tot}} = R_{\mathrm{amb,p}} + R_{\mathrm{amb,SEI}} = \frac{d_{\mathrm{p}}}{\sigma_{\mathrm{amb,p}}} + \frac{d_{\mathrm{SEI}}}{\sigma_{\mathrm{amb,SEI}}} . \qquad \textbf{(eq. S1)}$$

The growth rate is inversely proportional to this total resistance. The proportionality constant C includes the thermodynamic driving force and necessary material constants:

$$C = \frac{M_{\mathrm{SEI}} \cdot \Delta\mu_{\mathrm{Me}}}{F^2 \cdot \rho_{\mathrm{SEI}} \cdot x_{\mathrm{Me}}} . \qquad \textbf{(eq. S2)}$$

As a result, the differential equation for the growth rate is:

$$\frac{\mathrm{d}(d_{\mathrm{SEI}})}{\mathrm{d}t} = \frac{C}{R_{\mathrm{amb,tot}}} = \frac{C}{\frac{d_{\mathrm{p}}}{\sigma_{\mathrm{amb,p}}} + \frac{d_{\mathrm{SEI}}}{\sigma_{\mathrm{amb,SEI}}}} . \qquad \textbf{(eq. S3)}$$

To find the thickness at a given time, we separate the variables and integrate time from 0 to t and thickness from 0 to d_{SEI}:

$$\int_0^{d_{\mathrm{SEI}}} \left(\frac{d_{\mathrm{p}}}{\sigma_{\mathrm{amb,p}}} + \frac{d_{\mathrm{SEI}}}{\sigma_{\mathrm{amb,SEI}}} \right) \mathrm{d}(d_{\mathrm{SEI}}) = \int_0^t C \mathrm{d}t$$
$$= \frac{d_{\mathrm{p}}}{\sigma_{\mathrm{amb,p}}} d_{\mathrm{SEI}} + \frac{d_{\mathrm{SEI}}^2}{2 \cdot \sigma_{\mathrm{amb,SEI}}} = C \cdot t . \qquad \textbf{(eq. S4)}$$

This can be transferred into the standard form of a quadratic equation:

$$d_{\mathrm{SEI}}^2 + 2\sigma_{\mathrm{amb,SEI}} \cdot \frac{d_{\mathrm{p}}}{\sigma_{\mathrm{amb,p}}} \cdot d_{\mathrm{SEI}} - 2\sigma_{\mathrm{amb,SEI}} \cdot C \cdot t = 0 . \qquad \textbf{(eq. S5)}$$

Solving for d_{SEI} yields:

$$d_{\mathrm{SEI}} = -\sigma_{\mathrm{amb,SEI}} \cdot \frac{d_{\mathrm{p}}}{\sigma_{\mathrm{amb,p}}} \pm \sqrt{\left(\sigma_{\mathrm{amb,SEI}} \cdot \frac{d_{\mathrm{p}}}{\sigma_{\mathrm{amb,p}}} \right)^2 + 2\sigma_{\mathrm{amb,SEI}} \cdot C \cdot t} . \qquad \textbf{(eq. S6)}$$

Obviously, only a positive SEI thickness is physically meaningful ($\pm \rightarrow +$).

By factoring out $\sigma_{\mathrm{amb,SEI}}$ and substituting C back into the equation, **eq. 5** of the main manuscript is obtained.

S2. Derivation of $\Delta\mu_{Li}$ of the Decomposition Reaction at the Li|Li_6PS_5Cl Interface

The chemical potential of lithium μ_{Li}(electrode) in the lithium metal electrode is assumed to be equal to the standard chemical potential of lithium (μ_{Li}^0). Consequently, the lithium chemical potential difference across the interphase ($\Delta\mu_{Li}$), is given by $\Delta\mu_{Li} = \mu_{Li}(\text{reaction front}) - \mu_{Li}^0$.

$$Li_6PS_5Cl + 8\ Li^0 \rightarrow 5\ Li_2S + Li_3P + LiCl \qquad \textbf{(eq. S7)}$$

For the proposed decomposition reaction at the Li|Li_6PS_5Cl interface (see **eq. S7**), the stoichiometric factor x_{Li} is 8.

Thus, for the stoichiometric decomposition of Li_6PS_5Cl, $\Delta\mu_{Li}$ is approximated by the corresponding change of standard free energy $\Delta_r G$ for the decomposition of Li_6PS_5Cl, divided by x_{Li}:

$$\Delta\mu_{Li} = \frac{\Delta_r G}{x_{Li}} \qquad \textbf{(eq. S8)}$$

Based on available thermodynamic data,[3] $\Delta_r G(Li_6PS_5Cl)$ is −1269.75 kJ mol^{-1}. This yields an estimated $\Delta\mu_{Li}$ of approximately −158.7 kJ mol^{-1} for the decomposition reaction at the Li|Li_6PS_5Cl interface.

S3. The Influence of the Spatial Contact Distribution

To study the influence of contact distribution on the impedance data and derived interphase growth kinetics (k'_{exp}), we conducted a series of simulations. In this series, the relative contact area, A_r, was held constant at 10% of the electrode area, $A_{electrode}$, but the size and spatial distribution of the contact spots was varied (see **Figure S1a**). The resulting impedance spectra shown in **Figure S1b** reveal that R_{cstr} decreases with a finer contact distribution, while ($R_{tot} - R_{bulk}$) seems to increase with the same rate, independent of the contact geometry. This is also reflected in the extracted resistance curves (vs. t and $t^{0.5}$), as shown in **Figure S1c**. They only differ in their offset along the y-axis due to the varying R_{cstr}, while their slope in the linearized plot is equal. The differences in R_{cstr} arise from differences in the DC potential distribution in the SE (**Figure S1d**).

At time t_0, the potential drop close to the interface is more pronounced for coarse contacts than for fine contacts: A finer contact distribution leads to a more homogenous potential distribution close to the interface and thus a smaller potential drop and lower R_{cstr}. For a fine and homogenously distributed arrangement of contacts, it is likely that R_{cstr} is negligible compared to R_{tot}, although the actual A_r might be considerably lower than $A_{electrode}$.[4]

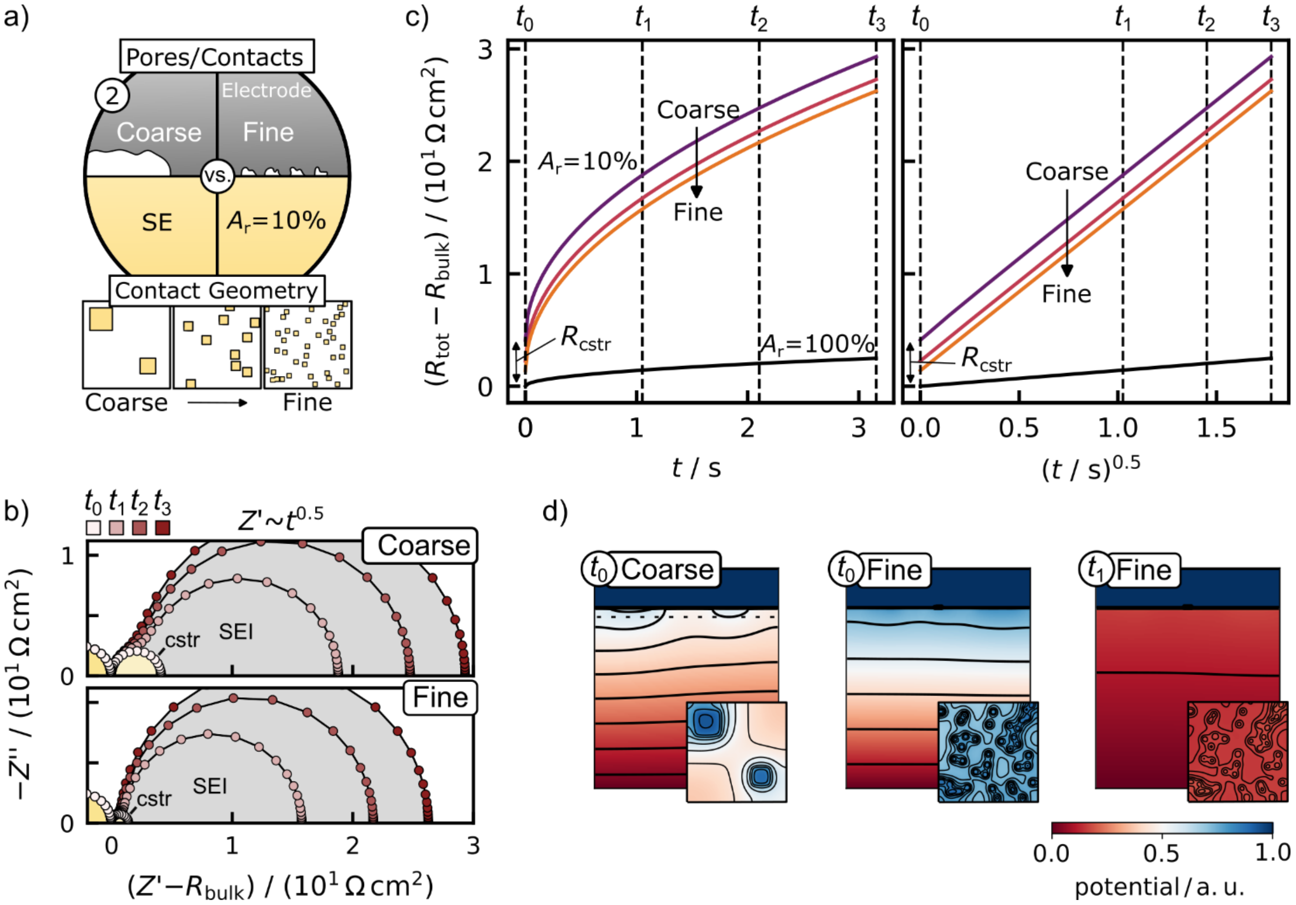

Figure S1. Effect of Contact Distribution on Apparent SEI Growth Kinetics. The *intrinsic*, *local* SEI growth rate k' was kept constant in the simulations. (a) Contact spots of different sizes are randomly distributed at the interface with a constant A_r of 10% between a homogeneous electrode and SE. (b) The constriction signal in the impedance spectrum decreases with finer contact distribution, while the SEI signal is unaffected. (c) The macroscopic rate constant k'_{exp}, describing SEI resistance growth over time, is independent of the contact distribution. It solely depends on A_r. The contact distribution affects only the magnitude of the constriction resistance R_{cstr}, resulting in a shift along the y-axis of the resistance curves. (d) The cumulative DC potential distribution shows that the pore-induced potential drop is more pronounced with a coarse contact distribution.

S4. The Influence of Native, Partially Penetrated Surface Passivation – Surface Coverage

To investigate how the areal coverage with passivation layer impacts impedance data and extracted rate constants (k'_{exp}), we conducted another series of simulations. Here, R_p was fixed at $4R_{ref}$ (with $R_{ref} = 9\ \Omega\cdot cm^2$) and randomly distributed contact spots were introduced at the interface. A_r, defined as the fraction of the interface where SEI formation occurs (*i.e.*, where the passivation layer is penetrated by SE surface asperities), was systematically varied from 1% to 99% of $A_{electrode}$ (see schematic in **Figure S2a**). Thus, the remaining fraction $(1 - A_r)$ was assumed to be covered by an intact passivation layer.

Figure S2b shows the time evolution of ($R_{tot} - R_{bulk}$). With increasing A_r, the transition from a square-root-of-time dependence to a time-independent ($R_{tot} - R_{bulk}$) takes more time. For large values of A_r, such as 99%, the transition is not fully captured within the simulation timeframe (up to t_2), resulting in an almost ideal linear dependence in the plot of ($R_{tot} - R_{bulk}$) vs. $t^{0.5}$. In contrast, at a small A_r (*e.g.*, 1%), the resistance evolution resembles a smoothed step function: The resistance appears to rise rapidly at first, but quickly saturates. The size of the saturated resistance increases with increasing A_r, while the initial resistance offset R_{init} becomes smaller.

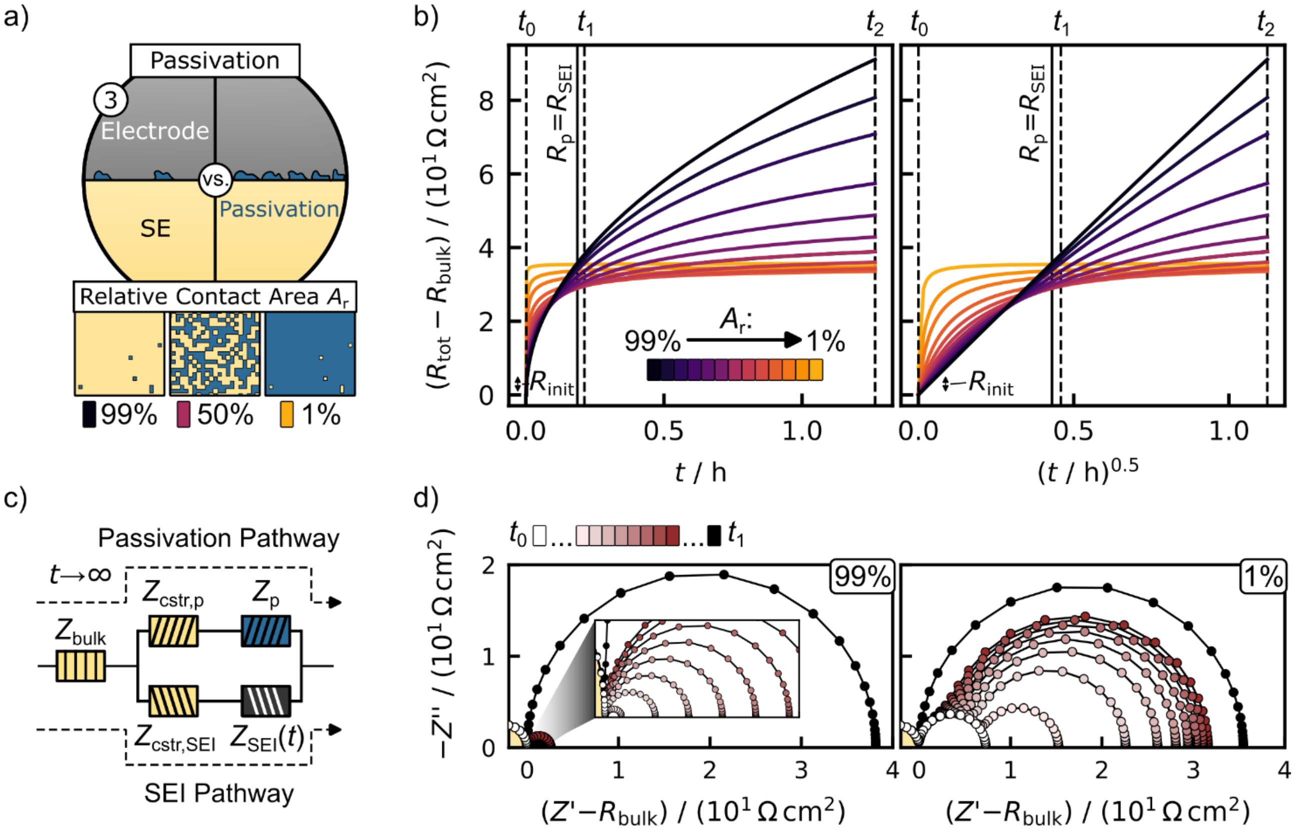

Figure S2. Effect of Contact and Passivation Layer Fraction on *Apparent* SEI Growth Kinetics. The intrinsic interphase growth rate k', and the interlayer resistance $R_p = 4R_{ref}$ were kept constant in the simulations. (a) A_r at the interface between a homogeneous electrode and SE is systematically varied between 1% and 99% by randomly flipping individual voxels. (b) As A_r decreases, there is a transition in the resistance evolution curves from a square root time behavior (black) to an almost instantaneous increase (i.e., step function) that reaches a plateau (yellow). Note that each curve reaches a plateau for sufficiently long simulation times. (c) The behavior results from competing transport pathways at the interface, where the size of the initial resistances is determined by A_r. (d) At t_1, the interface impedances are approximately the same for a nearly intimate (99%) or non-ideal (1%) contact, but the time evolution is significantly different.

The resistance evolution can be well understood by considering the simple equivalent circuit model depicted in **Figure S2c**. It consists of a bulk impedance Z_{bulk} that is connected in series to two parallel interfacial pathways characterized by transport through the passivation Z_p or across the growing SEI $Z_{SEI}(t)$.

Each interfacial pathway includes an individual constriction impedance ($Z_{\mathrm{cstr,p}}$ and $Z_{\mathrm{cstr,SEI}}$) due to current focusing near the interface. This constriction impedance differs for the SEI and interlayer pathway due to differences in coverage and spatial distribution of the respective phases.

Both R_{p} and $R_{\mathrm{SEI}}(t)$ are anti-proportional to the areal coverage of the interface with the respective phases. R_{SEI} becomes smaller when A_{r} becomes larger. In contrast, R_{p} becomes larger when A_{r} becomes smaller. The same trends are observed for the changes in $R_{\mathrm{cstr,p}}$ and $R_{\mathrm{cstr,SEI}}$. As a result of the decrease of $R_{\mathrm{SEI}}(t)$ with increasing A_{r}, the time required to reach the resistance plateau is increased. These trends are also reflected in the impedance spectra shown in **Figure S2d**. At A_{r} = 99% of $A_{\mathrm{electrode}}$, the initial impedance is low but increases steadily over time. In contrast, at $A_{\mathrm{r}} = 1\%$ of $A_{\mathrm{electrode}}$, the initial impedance is larger, but saturation occurs much earlier in time. At time t_1, the impedance of the system with A_{r} = 99% already exceeds that of the system with A_{r} = 1%.

R_{init} is also evident in the impedance data: At t_0, before any SEI has formed, the impedance of the system with A_{r} = 99% of $A_{\mathrm{electrode}}$ closely matches the bulk response. At this point, current predominantly flows through the SEI pathway, which spans most of the interface. In contrast, when A_{r} = 1% of $A_{\mathrm{electrode}}$, the small interface coverage leads to a significant initial constriction impedance, which manifests as an additional contribution clearly visible in the impedance data at t_0. The signal roughly corresponds to the constriction impedance of the SEI pathway, neglecting small fractions of the current that may already flow through the passivation layer at that time.

S5. Transition Time Estimation

If the transport properties and thickness of the passivation layer are known, the transition time (t_{trans}), which marks the shift of preferred transport from across the SEI layer to through the passivation layer, can be estimated. The transition occurs when $R_{\mathrm{SEI}}(t)$ and R_{p} become comparable, *i.e.*, when $R_{\mathrm{SEI}} \approx R_{\mathrm{p}}$. Assuming that they are identical, t_{trans} can be calculated via:

$$t_{\mathrm{trans}} = \left(\frac{R_{\mathrm{p}} \cdot A_{\mathrm{p}}}{k' \cdot A}\right)^2 \qquad \textbf{(eq. S9)}$$

Where R_{p} and A_{p} are the ionic resistance and contact area of the passivation layer, respectively, k' is the parabolic rate constant for the ionic resistance R_{SEI} of interphases (**eq. 3** of the main text), and A denotes the contact area between the SE and the electrode. R_{p} can be calculated from the ionic conductivity and layer thickness of the passivation layer. For passivated lithium foils, Otto *et al.*[5,6] reported passivation thicknesses ranging from 5 nm to 65 nm.

Based on literature values, the primary components of these native films (*e.g.*, Li_2O, Li_2CO_3, LiOH, Li_3N) exhibit ionic conductivities between 10^{-4} and 10^{-10} S cm^{-1}.[7–12] Assuming a negligible partial electronic conductivity and $A_p = 50\%$ of $A_{electrode}$, the estimated t_{trans} can vary widely from seconds up to several years.

The timeframe in which this transition can be observed is a function of R_p. At t_{trans}, the local current density for transport through the passivation layer begins to exceed that of transport through the SEI layer. However, this local shift in preferred current pathways does not immediately manifest in the macroscopic impedance: A measurable change at the cell level becomes apparent when the *total* current through the interlayer becomes significant (*i.e.*, comparable in magnitude to the current through the SEI). This second transition is decisive and depends not only on the interfacial transport properties but also on the relative areal coverage of the interface with SEI and passivation.

S6. The Influence of Passivation Layer Electronic Conductivity on SEI Resistance Growth

To investigate the influence of the partial electronic conductivity of the passivation layer (interlayer), the evolution of the areal ionic resistance of a single contact spot ($R_{ion,spot}$,) was simulated using the Deal-Grove model detailed in **Section S1** and **Section 2.2** (main text). The partial electronic and ionic conductivities of the SEI ($\sigma_{ion,SEI}$ and $\sigma_{eon,SEI}$) were adapted from the work of Alt *et al.*[13] The density of the SEI (ρ_{SEI}), molar mass (M_{SEI}) and the stoichiometric factor (x_{Li}) were also adopted from Alt *et al.*[13] The initial areal ionic resistance of the passivation layer ($R_{ion,p}$) was set to 9 Ω·cm^2, consistent with the prior simulations, whereas its areal electronic resistance ($R_{eon,p}$) was varied between $1\cdot10^0$ Ω·cm^2 to $1\cdot10^7$ Ω·cm^2. Assuming a 10 nm thick passivation layer, this range translates to a partial electronic conductivity ($\sigma_{eon,p}$) between $1\cdot10^{-6}$ S·cm^{-1} and $1\cdot10^{-13}$ S·cm^{-1}.

A collection of simulated $R_{ion,spot}$ curves plotted against t and $t^{0.5}$ are shown in **Figure S3**a and **Figure S3**b, respectively. For low values of $\sigma_{eon,p}$ (high $R_{eon,p}$), only the linear growth regime is visible in the time plot (**Figure S3**a), which mathematically translates to a parabolic curve in the $t^{0.5}$-plot (**Figure S3**b). For long times ($t\rightarrow\infty$), all curves converge toward a SEI-limited regime. In this late stage, they all exhibit a linear dependence against $t^{0.5}$ with an identical slope, defined by the intrinsic rate constant k'.

The contour plot displayed in **Figure S3**c summarizes the change in resistance ($\Delta R_{\mathrm{ion,spot}}$) over the timeframe of the simulations of the main text (≈ 1h) as a function of $R_{\mathrm{eon,p}}$. For instance, a $\Delta R_{\mathrm{ion,spot}}$ of 1 Ω·cm^2 indicates that the total spot resistance has increased from the initial 9 Ω·cm^2 (the ionic resistance of the pure passivation layer) to 10 Ω·cm^2 due to SEI growth.

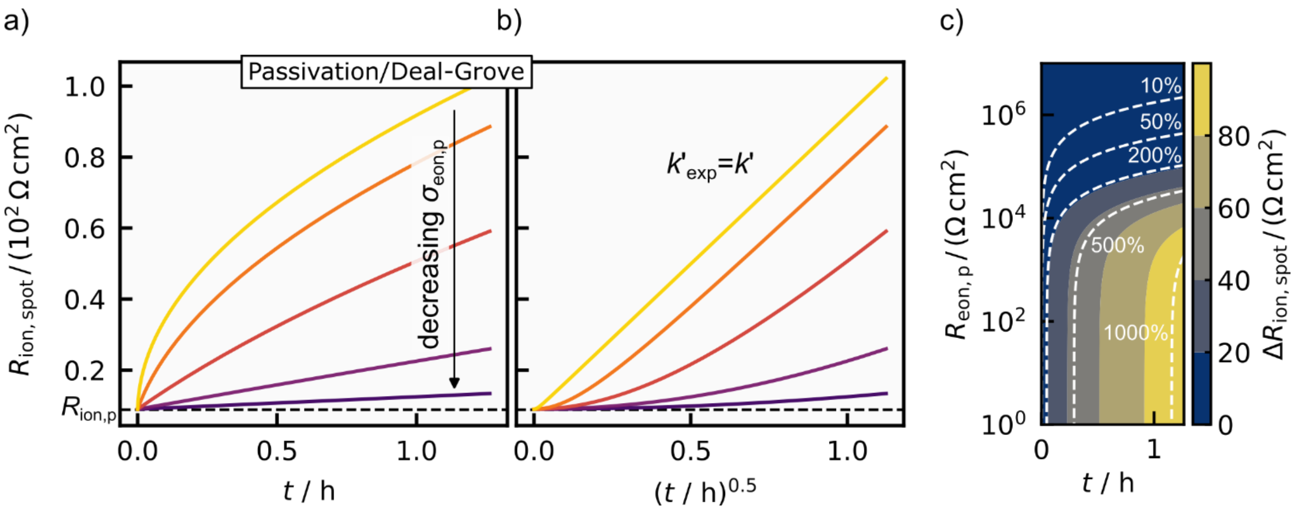

Figure S3. Effect of Passivation Layer Electronic Conductivity on *Apparent* SEI Resistance Growth. Simulations were performed using the adapted Deal-Grove model (Section S1) for pre-passivated contacts. (a) The evolution of the areal ionic spot resistance ($R_{\mathrm{ion,spot}}$) versus time (t) for varying electronic conductivities ($\sigma_{\mathrm{eon,p}}$) of the initial passivation layer. (b) The same resistance data plotted against $t^{0.5}$. (c) A contour map illustrating the total change in ionic resistance ($\Delta R_{\mathrm{ion,spot}}$) over time as a function of the passivation layer's areal electronic resistance ($R_{\mathrm{eon,p}}$). Dashed white lines indicate relative resistance increases compared to the initial 9 Ω·cm^{2}of the pure passivation layer.

The superimposed dashed contour lines highlight the exact coordinates where $\Delta R_{\mathrm{ion,spot}}$ reaches 10%, 50%, 200%, 500% and 1000% of the initial 9 Ω·cm^2 baseline. In summary, the contour plot reveals that $R_{\mathrm{eon,p}}$ must exceed 10^6 Ω·cm^2 to restrict the total contact spot resistance growth to less than 10% over the timeframe of the simulation (*i.e.*, $R_{\mathrm{ion,spot}}$ < 10 Ω·cm^2). For a 10 nm thick passivation layer, this requires a partial electronic conductivity of < 1·10^{-12} S·cm^{-1}. For comparison, in the absence of any passivation layer—or with a layer possessing negligible ionic and electronic resistance—the SEI resistance would rapidly grow by ≈94 Ω·cm^2 within the same timeframe.

S7. FIB-SEM Cross-Sections after Joining

To investigate the extent to which the applied joining and stack pressures affect the mechanical contact between the solid electrolyte (SE) and the lithium metal foil (Li), symmetric cells were prepared under varying pressure conditions.

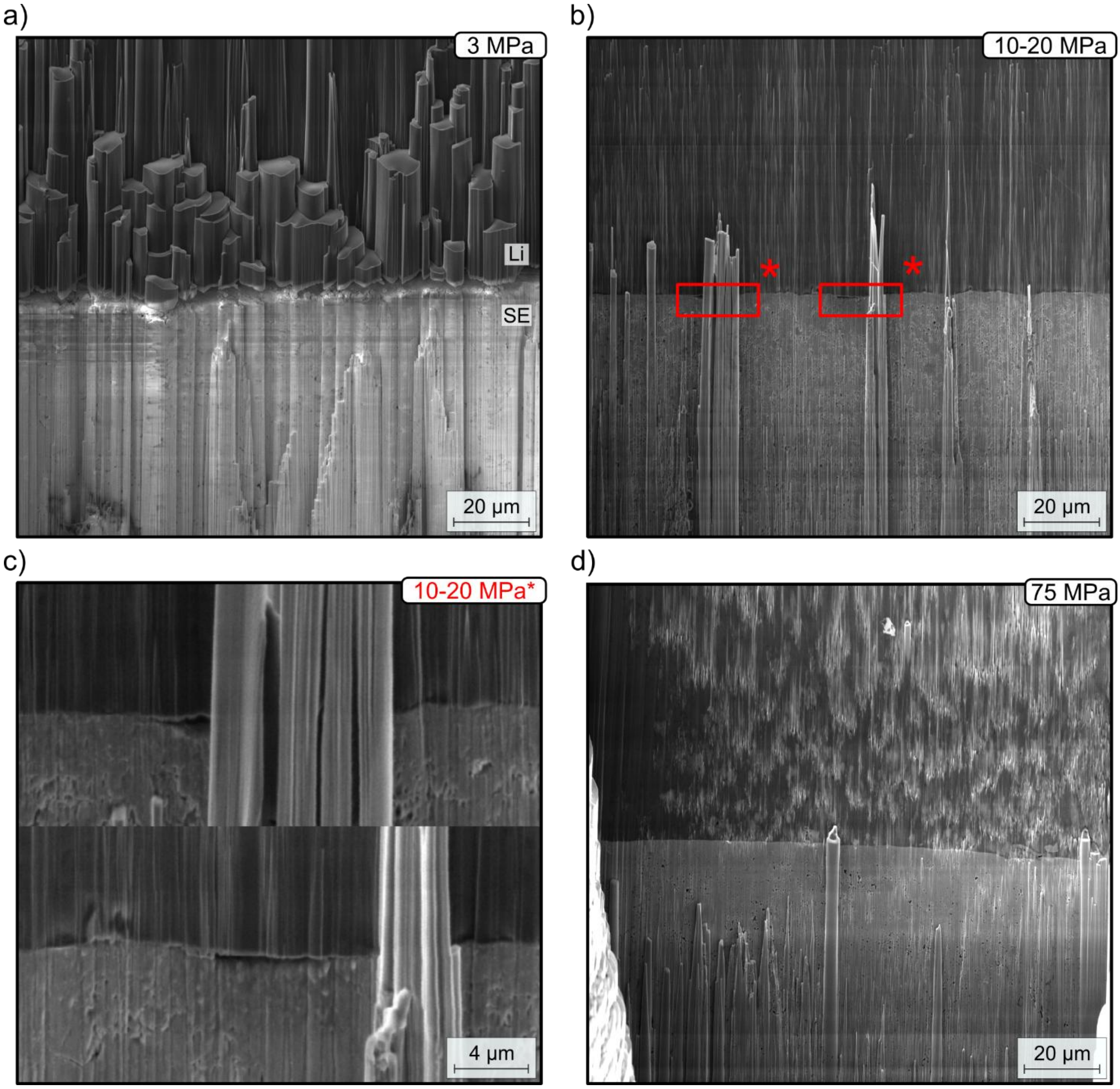

Figure S4. SEM Images of Li|Li_6PS_5Cl Cross-Sections after Joining at Varying Pressures. (a) Cross-section of a Li|Li_6PS_5Cl interface joined in a press-cell type setup at 3 MPa. (b) Cross section of a Li|Li_6PS_5Cl interface isostatically joined at 10-20 MPa. (c) Enlarged sections from (b) highlighting visible interfacial porosity. (d) Cross-section of a Li|Li_6PS_5Cl interface isostatically joined at 75 MPa.

Initial attempts to prepare a cell in the press-cell setup using a uniaxial stack pressure of 1 MPa resulted in immediate delamination of the lithium electrode upon removal of the steel piston. Consequently, the joining pressure was slightly increased to 3 MPa for the preparation of cross-sections.

While this prevented macroscopic detachment of the electrode, the subsequent FIB cross-section revealed poor to negligible interfacial contact (**Figure S4**a). This observation suggests that for press cells investigated within the 1 MPa to 20 MPa range, the mechanical contact area does change significantly with pressure.

To evaluate higher-pressure regimes, FIB cross-sections were also performed on isostatically pressed cells at 10MPa-20 MPa, 75 MPa (**Figure S4**b and **Figure S4**c), 20 MPa (main text), and 350 MPa (main text). In the pressure range between 10 MPa-20 MPa, we could not precisely adjust the pressure of our isostatic press to 10 MPa, therefore we denote it here with 10 MPa-20 MPa. For the respective cell, a few small interfacial pores are identifiable (**Figure S4**c). However, given the microscopic field of view relative to the macroscopic Li|Li_6PS_5Cl interface, it is difficult to conclude whether these small isolated voids are representative of the entire contact area. Furthermore, despite utilizing cryogenic conditions during FIB milling, minor preparation-induced artifacts at the interface cannot be entirely ruled out. Overall, the cross-sections at 20 MPa, 75 MPa, and 350 MPa exhibit highly comparable morphologies, both demonstrating near-ideal, conformal contact. Consequently, we conclude that the vast majority of mechanical contact formation occurs within the pressure range of 0 MPa to 20 MPa, while further increasing the pressure to 75 MPa (or even larger) yields only minor visible changes.

S8. XPS Data of a Lithium Foil Cut from a Rod and Aged in Glovebox Atmosphere

X-ray photoelectron spectroscopy (XPS) measurements were performed on lithium foils cut from a rod in the respective glovebox with $p(N_2)/p \approx 300$ ppm ($p(H_2O)/p < 1$ ppm, $p(O_2)/p < 1$ ppm. The respective foils were stored for 1 day in glovebox atmosphere. To obtain qualitative depth-resolved information, XPS spectra were collected both in the pristine state (*i.e.*, without sputtering) and after successive gas cluster ion beam (GCIB) sputtering steps (2 min at 10 kV, 2 min at 20 kV, 5 min at 20 kV) and a final 5 min Ar^+-ion sputtering step at 4 kV. The resulting spectra are shown in **Figure S5**.

With increasing sputtering depth, the relative intensity of the Li_2CO_3 and LiOH signals (Li 1s: $Li_2CO_3 \approx 55.5$ eV, LiOH ≈ 54.7 eV; O 1s: $Li_2CO_3 \approx 531.7$ eV, LiOH ≈ 531.1 eV) progressively decreases, indicating that these species are primarily confined to the outermost surface region. Simultaneously, the intensity of the Li_2O signal (Li 1s ≈ 53.8 eV; O 1s ≈ 528.3 eV) and the signal assigned to lithium metal (Li^0, Li 1s ≈ 52.7 eV & plasmon-loss) increases. Moreover, after the last sputtering step, new spectral features emerge in the N 1s region that we be attribute to Li_3N (N 1s: 394.9 eV) and a lithium-nitrogen compound $Li_{3-x}N_{1-y}$ (N 1s: 397.5 eV).[5,6,14]

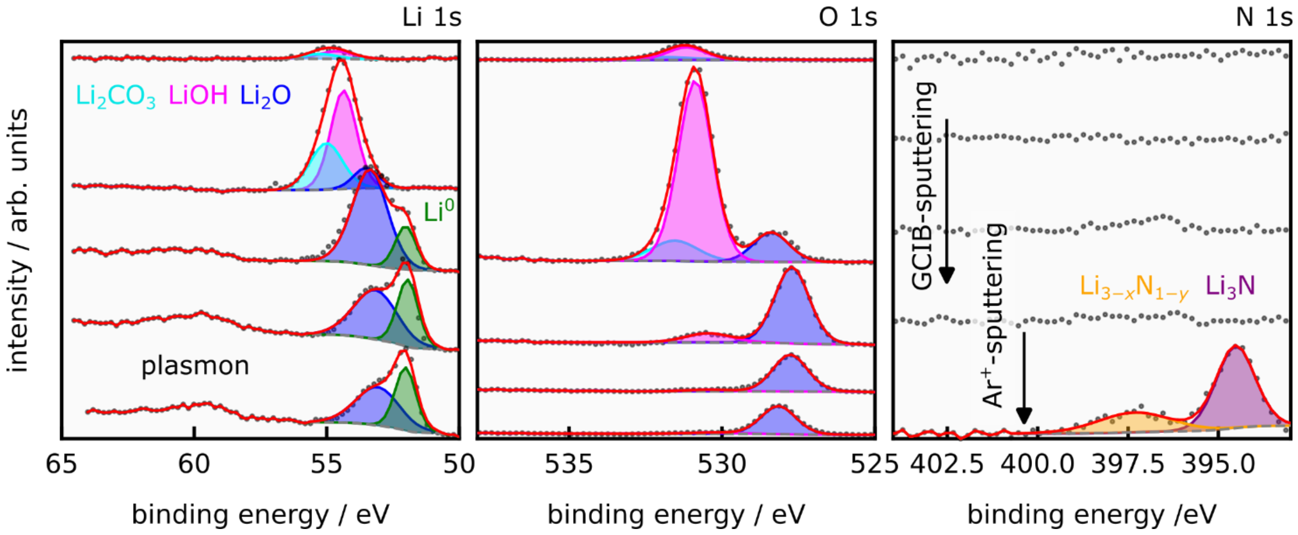

Figure S5. XPS Analysis of an Aged Lithium Foil Cut from a Rod. XP spectra of the Li 1s, O1 S, and N1 S regions of the pristine surface (top spectra) and after successive gas cluster ion beam (GCIB) sputtering steps (2 min at 10 kV, 2 min at 20 kV, 5 min at 20 kV) and a final 5 min Ar^+-ion sputtering step at 4 kV (from top to bottom). Raw data are displayed by circles and overall fits as red lines. Fits for individual chemical components are represented by shaded areas (magenta: Li_2CO_3, pink: LiOH, blue: Li_2O, green: Li^0). Within each row, spectra show the depth evolution from the pristine surface (top) to the last Ar^+-ion sputtering step.

S9. Detailed Impedance Data and Distribution of Relaxation Time

Impedance spectra for Li|Li_6PS_5Cl|Li cells isostatically joined at 20 MPa, 40 MPa, 75 MPa and 350 MPa are shown in **Figure S6a**. As the joining pressure increases, the impedance at frequencies below 10^6 Hz consistently decreases. The corresponding distribution of relaxation times (DRT) for these spectra are depicted in **Figure S6b**. The DRT data is plotted against frequency (f), aligning with standard experimental practice. The high-frequency contribution is primarily attributed to the bulk conductivity relaxation of the Li_6PS_5Cl solid electrolyte, including grain boundaries. However, this high-frequency regime may also be influenced by highly conductive components within the solid electrolyte interphase (SEI), such as Li_3P. As discussed in the simulation studies, the lower-frequency contributions are more complex and cannot be definitively assigned to individual phases or interphases. Instead, we attribute these features to a convolution of constriction effects, the SEI, and the native passivation layer on the lithium metal foil. Because the SEI is a composite[13,15] that likely includes mixed conductors (*e.g.*, Li_3P), the spectra may also be influenced by large chemical capacitances, which manifest as low-frequency relaxations.[16]

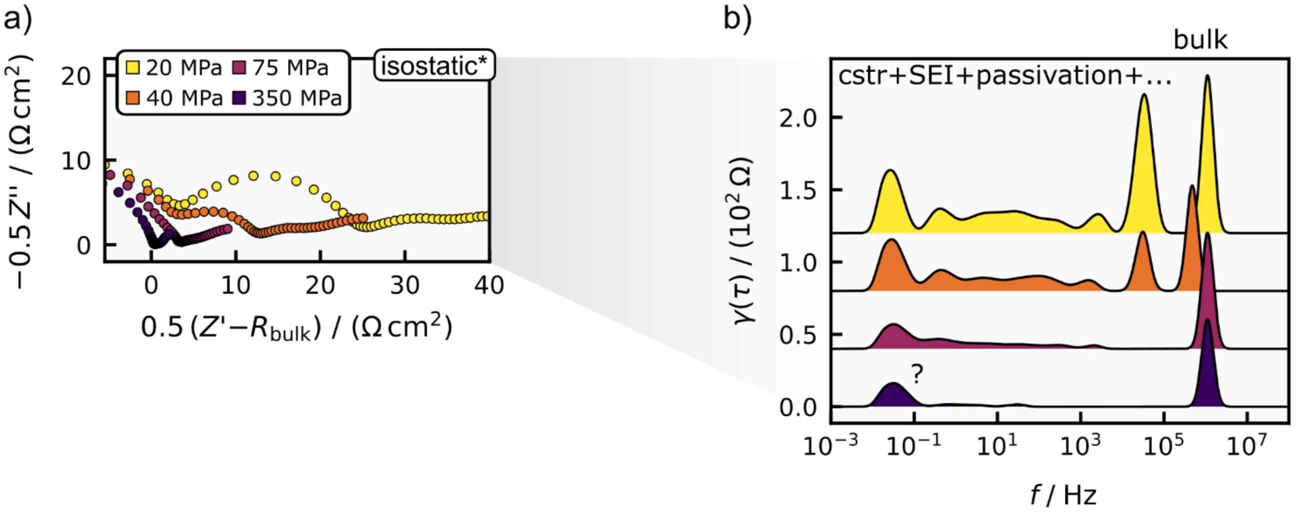

Figure S6. Joining Pressure Dependence of the Impedance Spectra of Symmetrical Li|Li_6PS_5Cl|Li cells. (a) Nyquist plots for Li|Li_6PS_5Cl|Li cells isostatically joined at 20 MPa, 40 MPa, 75 MPa and 350 MPa. (b) Corresponding DRT results.

Figure S7a displays the time evolution of an exemplary impedance spectrum for a Li|Li_6PS_5Cl|Li cell isostatically joined at 20 MPa. Over time, the impedance at frequencies below 10^6 Hz increases across a broad range. This broad increase is further reflected in the corresponding DRT profile shown in **Figure S7**b. While previous studies by Rieger *et al.*[17] and Wenzel *et al.*[15] assigned the SEI to the mid-frequency semicircle-like impedance contribution, we take an alternative approach and track the over-all change of the real part of the impedance with time (see main text).

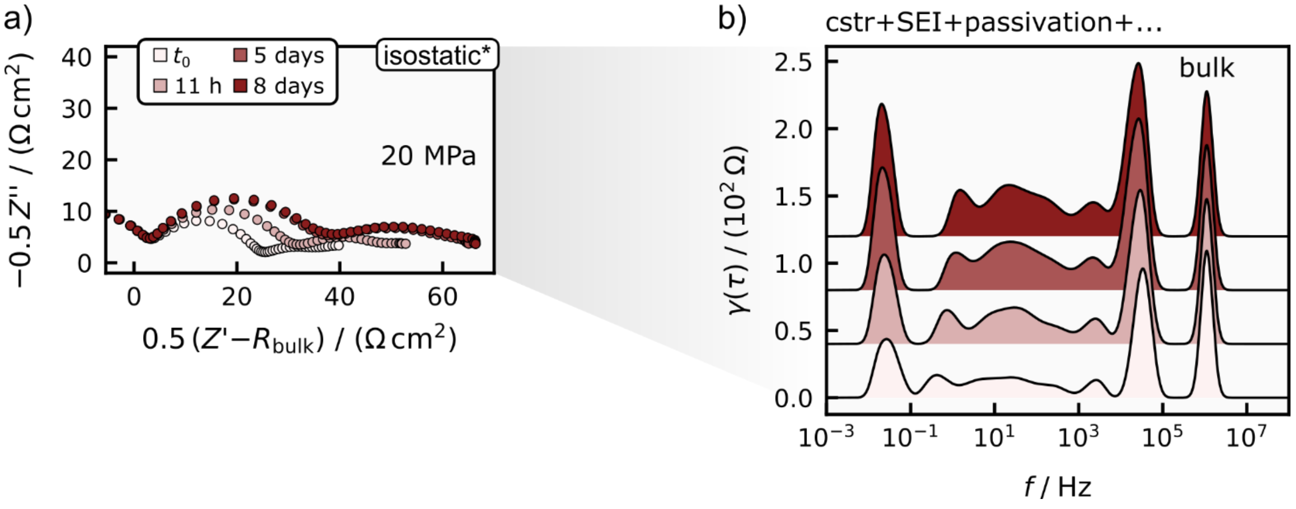

Figure S7. Time Evolution of the Impedance Spectrum of a Symmetrical Li|Li_6PS_5Cl|Li cell Joined at 20 MPa. (a) Nyquist plot at different times (t_0, 11 h, 5 days, 8 days). The inset highlights the impedance increase in the mid-frequency region (b) Corresponding DRT.

Similarly, **Figure S8**a illustrates the time evolution of an impedance spectrum for a cell isostatically joined at 75 MPa. Although the data reveals a slight impedance increase in the mid-frequency range (as highlighted in the inset), the primary impedance growth occurs at frequencies below 1 kHz.

This dominant low-frequency increase is also consistent with the impedance spectra obtained at 350 MPa. **FigureS8**b displays the corresponding evolution of the DRT over time and shows that most of the changes happen at frequencies below 10^3 Hz.

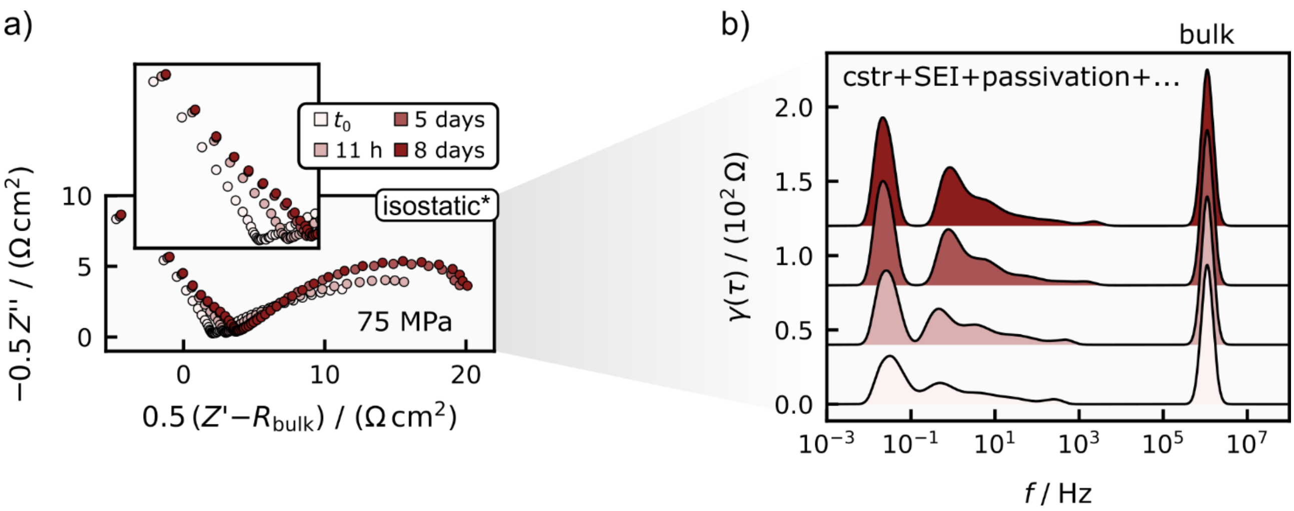

Figure S8. Time Evolution of the Impedance Spectrum of a Symmetrical Li|Li_6PS_5Cl|Li cell Joined at 75 MPa. (a) Nyquist plot at different times (t_0, 11 h, 5 days, 8 days). The inset highlights the impedance increase in the mid-frequency region (b) Corresponding DRT.

S10. Low-Frequency Impedance and Excitation-Voltage Dependence

To check the linearity of the impedance data, the AC excitation voltage ($\tilde{V}$) was systematically increased from 0.5 mV to 20 mV. We utilized a symmetric cell that had been isostatically pressed at 350 MPa and subsequently aged for over 40 days. Following this extensive resting period, the impedance exhibits minimal temporal drift, rendering it suitable for extended low-frequency measurements down to 1 mHz. Interestingly, as shown in **Figure S9**a, while the high-frequency impedance (likely governed by bulk and grain boundaries) remains perfectly stable and independent of the excitation voltage, the low-frequency impedance (apex frequency ≈ 10 mHz) is severely non-linear.

The total apparent interfacial resistance systematically decreases with increasing excitation voltage (**Figure S9**b), dropping from ~70 Ω·cm^2 at 0.5 mV to roughly 20 Ω·cm^2 at 20 mV. While the exact physical origin of this low-frequency process remains unknown, we hypothesize that it is driven by amplitude-dependent morphological changes at the solid-solid interface. Specifically, heterogeneous nucleation, electrocrystallization, and the reversible buildup and relaxation of localized mechanical stress may play a role. Note that all impedance spectra are collected with excitation voltages well below the thermal voltage of ≈ 25 mV (at 25 °C).

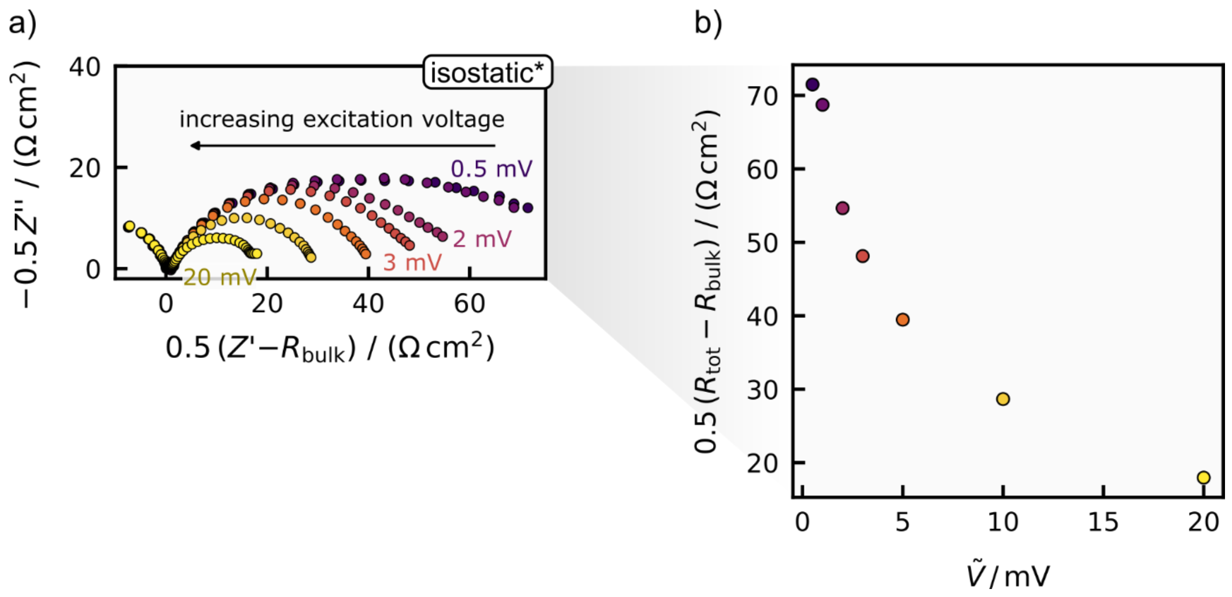

Figure S9. Non-Linear, Excitation-Voltage-Dependent Low-Frequency Impedance of a Symmetrical Li|Li_6PS_5Cl|Li cell. (a) Nyquist plots recorded at varying AC excitation voltages ($\tilde{V}$) ranging from 0.5 mV to 20 mV (0.5 mV, 1 mV, 2 mV, 3 mV, 5 mV, 10 mV, 20 mV). (b) $(R_{\mathrm{tot}} - R_{\mathrm{bulk}})$ plotted as a function of the applied excitation voltage. The lowest frequency measurement point is at 1 mHz.

S11. Model and Parameter Overview

We used three different models to fit the extracted resistance data. The simplest model, which reflects a series-type equivalent circuit model with a time dependent resistor, is the usual Wagner-type model:

$$(R_{\mathrm{tot}} - R_{\mathrm{bulk}}) \approx R_{\mathrm{SEI}} + R_{\mathrm{off}} = k'_{\mathrm{exp}} \cdot \sqrt{t - t_{\mathrm{off}}} + R_{\mathrm{off}}\,, \qquad \textbf{(eq. S10)}$$

where R_{off} is an additional series resistance that accounts for a resistive offset. As discussed in the main manuscript, the reason for this offset might be the constriction resistance related to current focusing on contact spots or deviations from a Wagner-type behavior in the early stage of SEI growth. By introducing a time offset, t_{off}, we consider the uncertainty in the time needed between joining the components and starting the actual measurement. The parameter was constrained between 4 min and 1 h.

Using the respective model, we fitted the resistance evolution during the whole 10 days but also performed fits where we targeted the later stage of the resistance evolution. For cells investigated under stack-pressure (press-cell setup), the first 6 days of the measurement, showing the sharp initial resistance increase and unsteady resistance behavior, were neglected. For the isostatically prepared cells, the timeframe was extended to the last 8 days of the measurement, excluding the initial sharp rise in resistance.

All fit parameters for the uniaxial cells and isostatic cells are displayed in **Table S2** and **Table S4**. Moreover, the pressure dependence of k'_{exp} and R_{off} are displayed in **Figure S10** (parameters fitted with restricted time windows). In general, a systematic decrease of k'_{exp} and R_{off} with pressure is obtained.

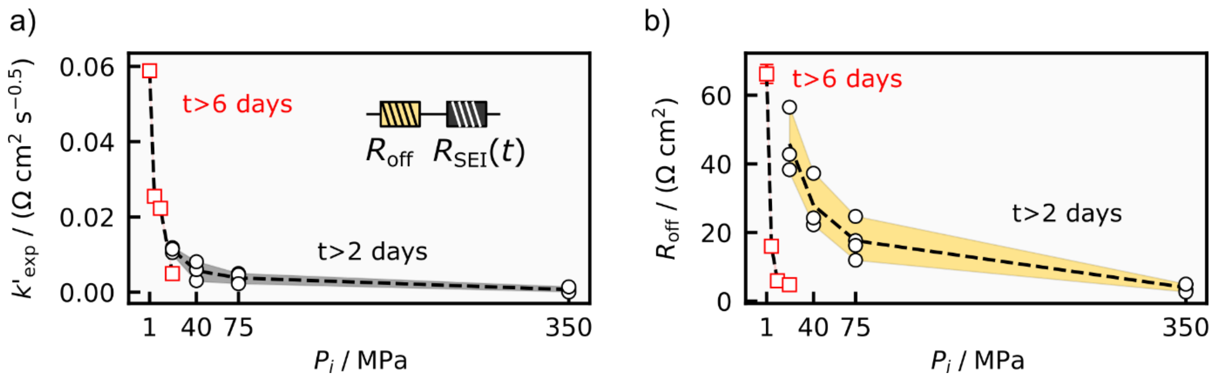

Figure S10. Pressure Dependence of the Wagner-Type Model Fit Parameters for the Later Stages of SEI Resistance Evolution. (a) The kinetic growth parameter k'_{exp} and (b) the resistive offset R_{off} plotted as a function of the (joining/stack) pressure P_i. Red squares denote the press-cells investigated under stack-pressure, where the first six days of the measurement were neglected. Black circles represent isostatically prepared cells, where the fitting timeframe was extended to the last 8 days of the measurement. The black, dashed line connects the average data points at each pressure, whereas the shaded regions indicate the minimum and maximum values, illustrating the spread of the data for the isostatically prepared cells.

In addition to the series-type model, parallel models were used for fitting, accounting for the presence of a partially penetrated native passivation layer on the lithium metal electrode. The parallel model considers the native passivation layer resistance R_{p} in series with a corresponding constriction resistance $R_{\mathrm{cstr,p}}$ and combines them into an effective passivation layer resistance $R_{\mathrm{p*}}$, *i.e.*, the resistance associated with current flow through passivation spots (see equivalent circuit model in **Figure S11**a). The passivation layer is connected in parallel to the constriction resistance R_{cstr} associated with current flow through SEI spots as well as the actual time-dependent SEI resistance R_{SEI}.

$$(R_{\mathrm{tot}} - R_{\mathrm{bulk}}) = \left(\frac{1}{R_{\mathrm{SEI}} + R_{\mathrm{cstr}}} + \frac{1}{R_{\mathrm{p*}}}\right)^{-1} = \left(\frac{1}{k'_{\mathrm{exp}} \cdot \sqrt{t - t_{\mathrm{off}}} + R_{\mathrm{cstr}}} + \frac{1}{R_{\mathrm{p*}}}\right)^{-1} \quad \textbf{(eq. S11)}$$

The model contains one more parameter compared to the series model from **eq. S10**. Therefore, we also used a simplified model without R_{cstr} for data fitting, relying on the assumption that the constriction resistance associated with current flow through SEI spots is small compared to the areal resistance of the SEI. All fit parameters are displayed in **Table S3** and **Table S5**.

Only the data of cells prepared via isostatic joining were fitted using the parallel models due to the unsteady resistance behavior obtained for the press cells. The pressure dependence of the parameters from **eq. S11** is displayed in **Figure S11**.

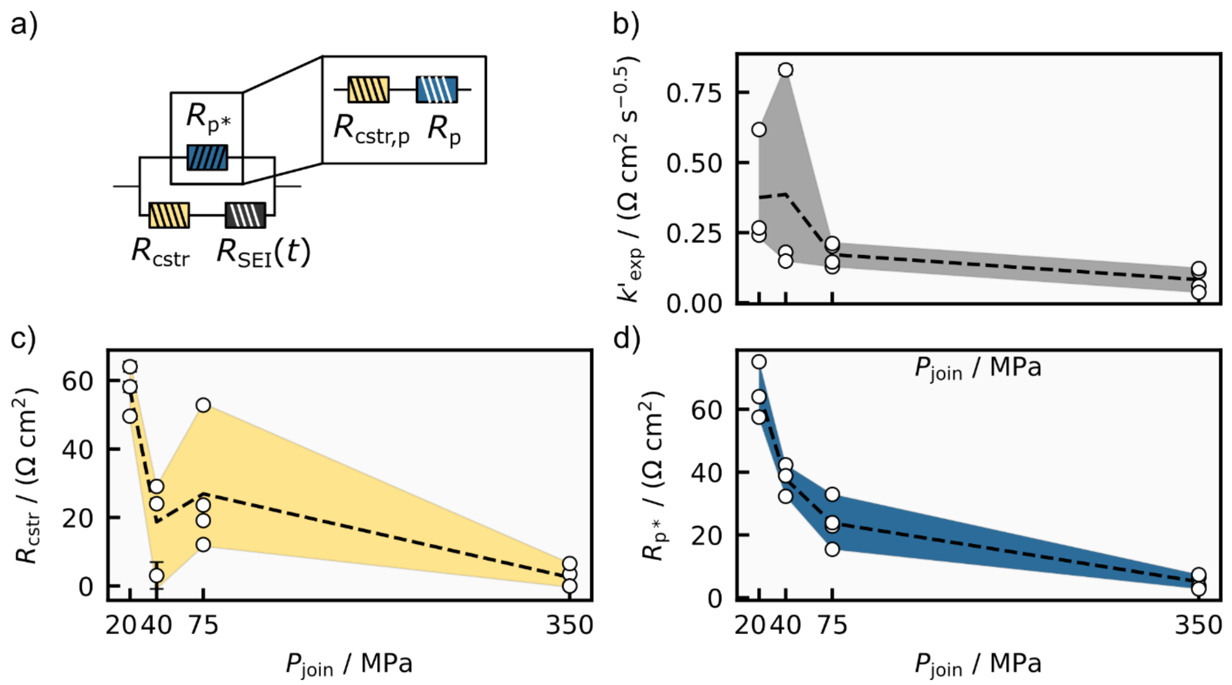

Figure S11. Joining Pressure Dependence of the Parallel Model Fit Parameters. (a) Schematic of the parallel equivalent circuit model accounting for the native passivation layer. The pressure dependence of the extracted fit parameters is shown for (b) k'_{exp}, (c) R_{cstr}, and (d) R_{p*}. The black dashed lines connect the average values at each pressure, and the shaded regions illustrate the spread (minimum to maximum) of the data. Fitting standard errors are displayed by error bars.

In general, a systematic decrease of k'_{exp}, R_{cstr}, and R_{p*} with increasing pressure is obtained. A few datapoints show a deviation from this trend, which we attribute to day-to-day variations in the glove-box atmosphere during cell assembly.

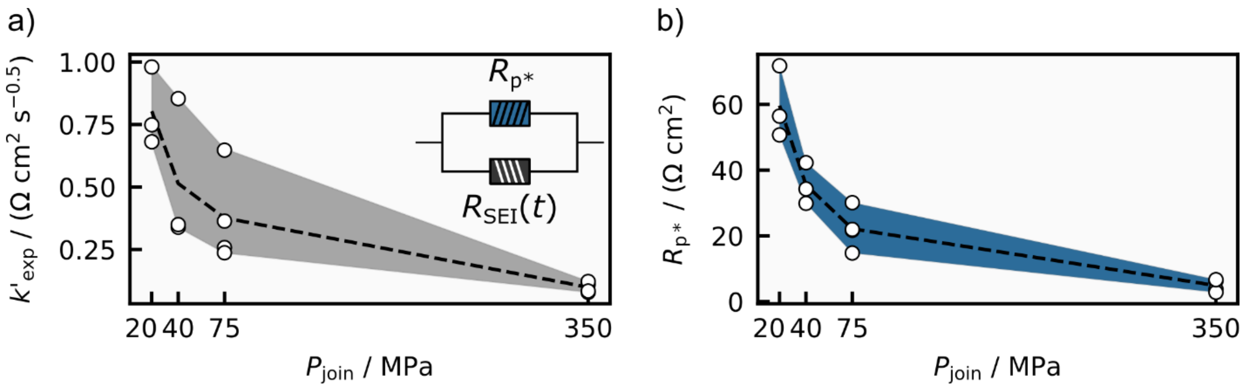

Figure S12. Joining Pressure Dependence of the Simplified Parallel Model Fit Parameters. (a) Pressure dependence of k'_{exp} and (b) R_{p*}. The inset in (a) displays the simplified equivalent circuit model, which relies on the assumption that R_{cstr} is small. The black dashed lines connect the average values at each pressure, and the shaded regions illustrate the data spread (minimum to maximum).

The pressure dependence of the parameters obtained with the simplified parallel model is shown in **Figure S12**. As for the previous models, a systematic decrease of k'_{exp} and $R_{\mathrm{p*}}$ with increasing pressure is obtained. Exemplary fitting curves, residuals and raw data are displayed in **Section S16**.

S12. CTTA Measurements

CTTA measurements were performed at 1 MPa, 4 MPa, 5 MPa, 13 MPa and 52 MPa in a press-cell setup equivalent to the one used by Aktekin *et al.*[18] The exact measurement procedure was also adopted from their work: 1 µAh of charge was plated in each titration step using a current of 10 µA on a circular stainless-steel disc electrode (0.64 cm^2 area) at 25 °C. The cutoff criterion for the resting period (OCV) was fixed at 0.05 V. The resulting cell voltage (E (vs. Li^+/Li)) over time for cells investigated at 1 MPa and 4 MPa is displayed in **Figure S13**a. The titration resting steps ($t_{\mathrm{OCV},i}$) for the 4 MPa cell are significantly shorter compared to the 1 MPa cell. This indicates that it takes longer in the 1 MPa cell to consume an equivalent amount of charge via SEI formation compared to the 4 MPa cell.

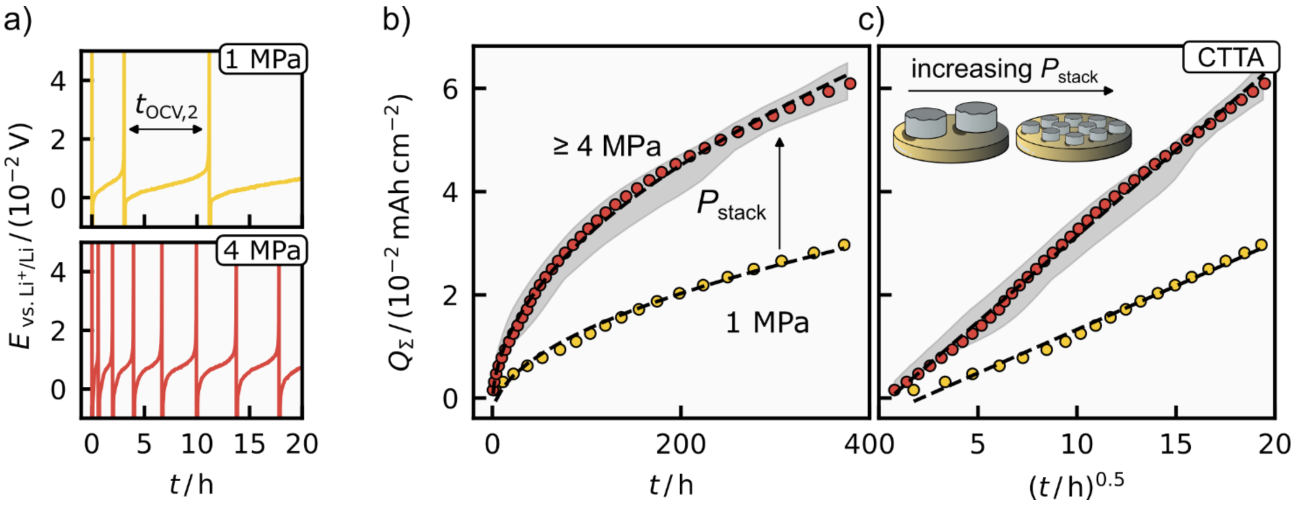

Figure S13. Stack Pressure Dependence of CTTA. (a) Cell-voltage profiles (vs. Li^+/Li) during CTTA at 1 MPa and 4 MPa, illustrating the differing durations of the open-circuit resting periods. (b) Cumulative charge consumption (Q_Σ) vs time (t). The grey shaded region bounds the data spread for measurements between 4 MPa and 52 MPa. (c) Q_Σ plotted against the square root of time. Dashed lines show fits with a square root model. The inset schematically illustrates the expected development of the lithium morphology at insufficient stack pressures compared to higher stack pressures.

The cumulative charge (Q_Σ) vs. time (t) and vs. square root of time ($t^{0.5}$) curves are displayed in **Figure S13**b and **Figure S13**c. The grey shaded area represents the maximum and minimum deviation from the 4 MPa baseline when increasing the stack pressure further up to 52 MPa (encompassing the measurements at 5 MPa, 13 MPa, and 52 MPa).

Increasing the pressure beyond 4 MPa up to 52 MPa resulted in no significant changes of lithium consumption over time. The cumulative curves show roughly a linear dependence vs. $t^{0.5}$, supporting a diffusion-controlled SEI growth mechanism.[2,15] Consequently, while increasing the pressure from 1 MPa to 4 MPa led to a slight increase in the slope of the cumulate charge curve (indicating faster lithium consumption), further increasing the pressure from 4 MPa to 54 MPa did not yield significantly different results.

To estimate rate constants for comparison with the impedance derived values, the cumulative charge curves were fitted using a square root model ($Q_\Sigma = k_{Q,\mathrm{exp}} \cdot \sqrt{t}$). Here, the parabolic rate constant $k_{Q,\mathrm{exp}}$ represents the rate constant for charge consumption. Assuming 1 μAh·cm^2 corresponds to an SEI thickness of ≈ 9 nm,[18] the rate constant for SEI thickness growth k_{exp} can be obtained by multiplying $k_{Q,\mathrm{exp}}$ by 9 nm·(μAh·cm^2)$^{-1}$. Based on the derived k_{exp} and the partial ionic conductivity of the SEI determined by Alt *et al.*[18] k'_{exp} can be estimated. The respective k'_{exp} values are summarized in **Table S6**.

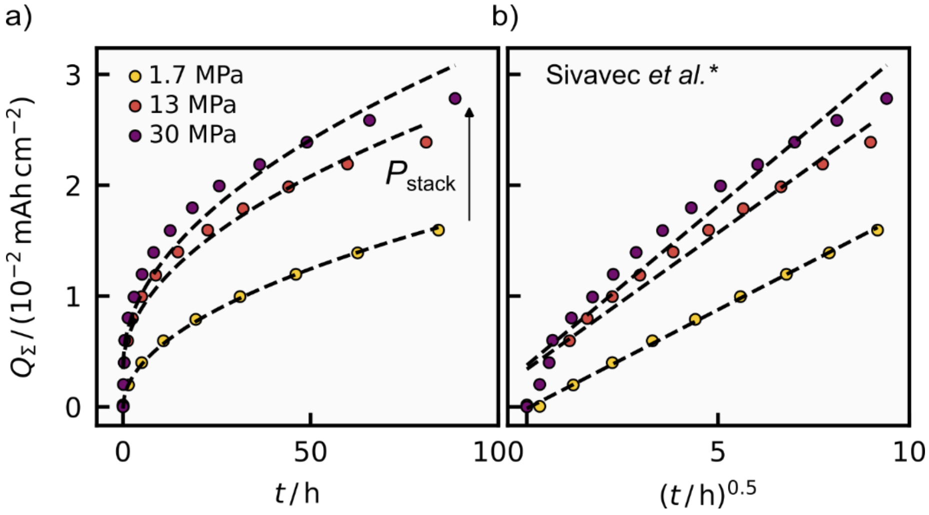

Figure S14. Stack Pressure Dependence of CTTA Obtained by Sivavec *et al.*[19] (a) Cumulative charge consumption (Q_Σ) vs time for varying applied stack pressures (1.7 MPa, 13 MPa, and 30 MPa). (b) Q_Σ plotted against the square root of time. Data points were extracted from the original publication using a digitization tool and fitted (dashed line) with a square root model.

In addition to our own measurements, we analyzed the data of Sivavec *et al.*[19] Data points were extracted from their original publication using a digitization tool and fitted using the identical square root model. The extracted data and corresponding fitting curves are shown in **Figure S14**. Similar to our results, the data from Sivavec *et al.* shows a slight increase in charge consumption with increasing pressure. The estimated rate constants for these datasets are also summarized in **Table S6**.

S13. Apparent Interphase Growth Kinetics After Electro-Dissolution and Plating

Two experiments were conducted to investigate the influence of lithium dissolution and plating on the *apparent* SEI growth trends. First, we examined the impedance during electro-dissolution (**Figure S15Figure S15**a-c). For this purpose, a cell joined at 350 MPa, which had already undergone over 10 days of aging, was subjected to a current density of 100 mA·cm^{-2}. Following every 3 h of electro-dissolution (and plating), the cell was rested for 30 min at open-circuit voltage (OCV), after which 16 impedance spectra were collected over approximately 1.1 h. The experiment ended when the cell voltage E reached 0.3 V.

The resulting voltage vs. time curve (**Figure S15**a) exhibits the characteristic evolution associated with contact loss at the dissolution electrode. Contact loss is further reflected in the impedance data collected during rest periods (**Figure S15**b). From step 1 to step 8, the impedance increased significantly. Specifically, during the resting periods for steps 2 through 6, a marked rise in impedance was observed. In contrast, the impedance remained roughly constant during step 1 (prior to current application); this aligns with the fact that the cell had already aged for 10 days. In the rest periods of steps 7 and 8, we observed a decrease in impedance over time. This is attributed to contact relaxation, which becomes prominent at small contact areas, likely driven by the atmospheric pressure acting on the vacuum sealed pouch cells.

The evolution of $(R_{\mathrm{tot}} - R_{\mathrm{tot},t_0})$ during these rest periods is shown in **Figure S15c** for steps 1–6. For steps 7 and 8, the data is omitted from the graph as the impedance decreased significantly due to the aforementioned contact relaxation. Notably, the impedance increase during rest periods scales with the decreasing contact area, *i.e.*, increasing overvoltage, on the electro-dissolution side. This trend was expected, given that measured resistance is inversely proportional to the electrochemical active contact area. While also the plating side of the cell may contribute, we hypothesize that the dissolution interface (*i.e.* the “anode”) is the primary driver of this resistance trend.

A second experiment was conducted to further probe these effects (**Figure S15**d-f). A separate pellet, also joined at 350 MPa and aged for more than 10 days, underwent lithium dissolution at 100 mA·cm^{-2} until a cut-off voltage of 0.3 V was reached (**Figure S15**d). Upon reaching the cut-off voltage, at which the remaining contact area between the SE and lithium metal is minimal, impedance spectra were collected. Again, contact relaxation caused the impedance to decrease over time (**Figure S15**d, inset). Subsequently, we plated back less than 10% of the dissolved lithium by reversing the current for 1 hour (**Figure S15**e), followed by continuous impedance tracking (**Figure S15e,** inset). The resulting resistance evolution versus time is compared in **Figure S15**f against the cell’s initial resistance evolution immediately after isostatic joining.

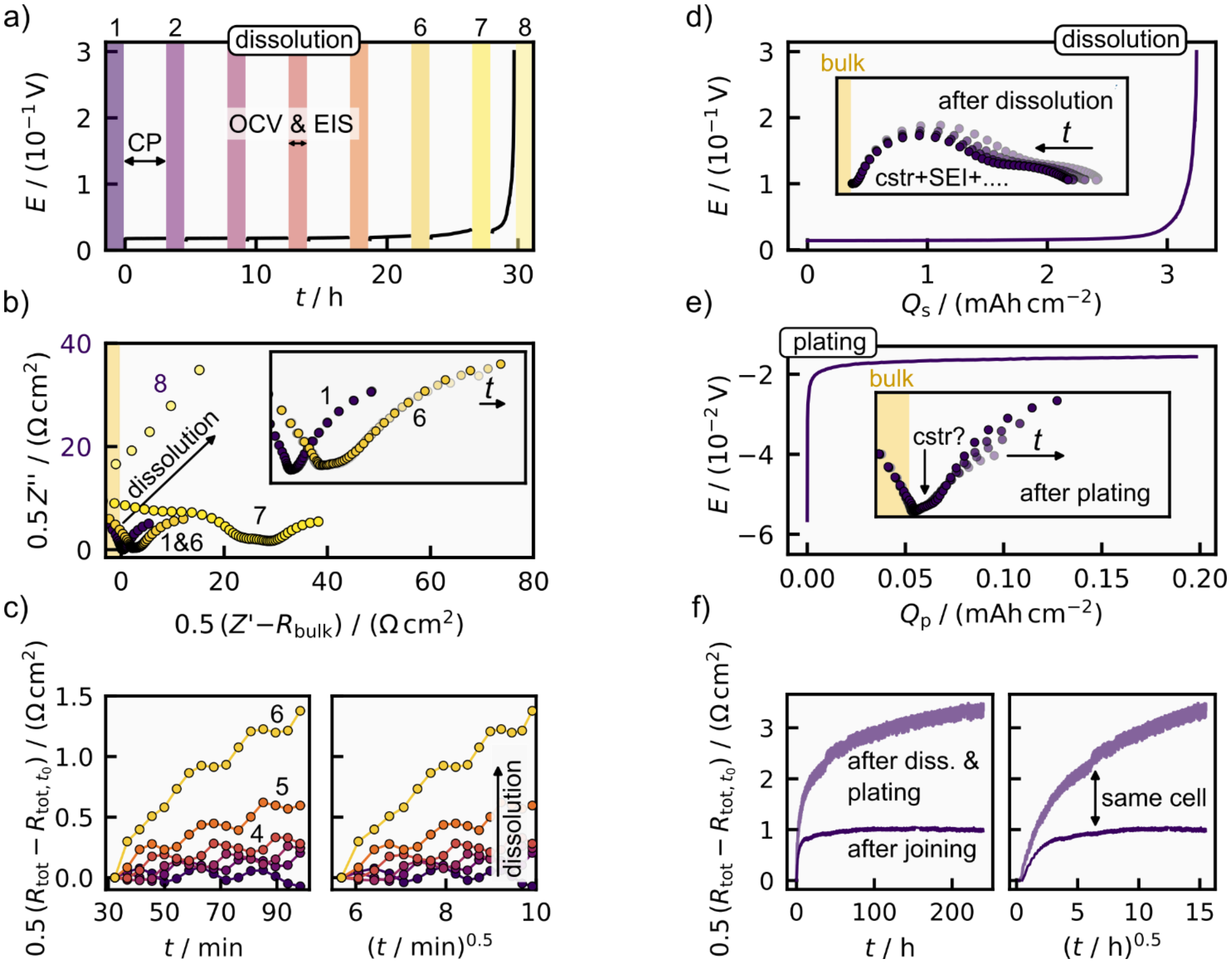

Figure S15. Effect of Electro-Dissolution and Plating on Apparent SEI Growth Kinetics. (a) Cell Voltage profile during lithium electro-dissolution at 100 mA·cm^{-2}. Vertical colored bars indicate periods of 30 min OCV and 1.1 h EIS measurements. The voltage rise toward the 0.3 V cut-off indicates progressive contact loss. (b) Nyquist plots illustrating the impedance evolution from Step 1 to Step 8. The inset highlights the impedance increase from Step 1 to Step 6 as well as the corresponding impedance increase during the resting period. (c) Time-dependent evolution of the extracted ($R_{tot} - R_{tot,t_0}$) during the resting periods for Steps 1–6. (d) Voltage profile for a second dissolution experiment reaching the 0.3 V cut-off. The inset shows the Nyquist spectra immediately after dissolution, demonstrating a decrease in impedance over time due to contact relaxation at the lithium/electrolyte interface. (e) Subsequent plating of <10% of the dissolved lithium back onto the previous dissolution side. The inset shows an increasing impedance with time after this plating step, indicative for SEI growth. (f) Comparison of ($R_{tot} - R_{bulk}$) as a function of time (left) and square-root-of-time (right) for the cell in its initial state (after isostatic joining) versus after the stripping/plating cycle.

The data indicates that after stripping and plating back only a fraction of the initial lithium, the apparent interfacial degradation is accelerated. This faster resistance growth is likely related to the reduced contact area of lithium with the SE. The initial significant rise in impedance suggests the formation of "fresh" SE-lithium contacts; however, follow-up experiments are required to confirm these observations and isolate their mechanistic origin.

S14. Truncation Error

To evaluate the truncation error of the 3D impedance simulations, the spatial resolution was systematically varied. Simulations were performed using voxel lengths ranging from 0.5 µm to 4 µm for a representative structure (analogous to the morphology in **Figure 3**, main text) with a relative contact area of approximately 8% (A_r). This was evaluated at three different simulated times: $t = 0$ s, $t = 12$ h, and $t = 24$ h. The resulting total resistances R_{tot} are plotted as a function of voxel length in the upper panels of **Figure S16**. To quantify the error, the theoretical continuum limit (voxel length → 0) was estimated for each time step by extrapolating the y-axis intercept via a linear fit. The relative truncation error was then calculated as the percentage deviation of the simulated resistance at a given resolution from this extrapolated ideal value.

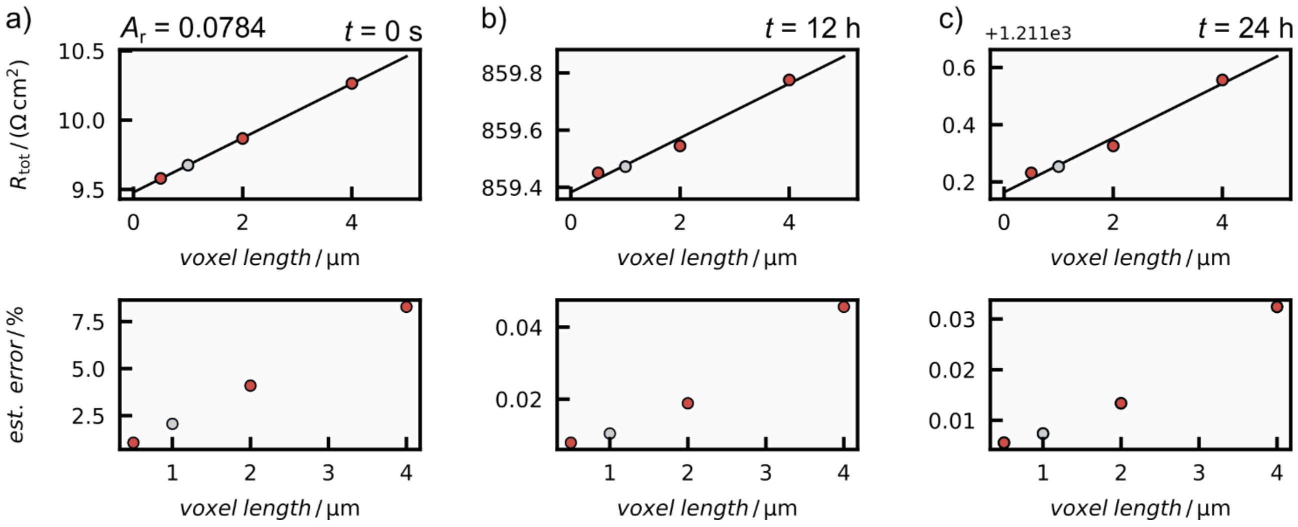

Figure S16. Spatial Resolution Test and Truncation Error Estimation for the 3D Impedance Simulations. The top row displays the simulated total resistance R_{tot} as a function of voxel length for a modeled interface with an 8% relative contact area at (a) $t = 0$ s, (b) $t = 12$ h, and (c) $t = 24$ h. Solid black lines represent linear extrapolations to a voxel length of zero. The bottom row displays the corresponding relative truncation error, calculated as the percentage deviation of the simulated resistance from the extrapolated y-intercept. The grey markers highlight the 1 µm voxel resolution utilized throughout this study.

As depicted in the bottom row of **Figure S16**, the maximum truncation error at the standard 1 µm resolution is approximately 2.5% at $t = 0$ s. At later times (12 h and 24 h), the total resistance is no longer governed by the spatially demanding constriction resistance—which is highly sensitive to the lateral resolution of sharp contact edges—but is instead dominated by the uniform solid electrolyte interphase (SEI) resistance. Consequently, the relative error drops drastically to below 0.05% for these later time steps.

S15. Amount of Lithium Metal being Electroplated and Dissolved During EIS

This section details the theoretical estimation of the thickness of lithium plated (or stripped) during a single half-cycle of an EIS measurement. A 1.5 mm thick pellet ($L = \text{thickness}$) with a SE conductivity (σ_{SE}) of 2 mS·cm^{-1} and electrode area (A) of 0.28 cm^2 (diameter of 6 mm) has a resistance of R = 265 Ω ($R = L/(\sigma \cdot A)$), which corresponds to a peak current amplitude of I_0 = 37.7 µA (excitation voltage U_0 = 10 mV, $I_0 = U_0/R$). In an EIS measurement, the current follows a sinusoidal profile:

$$I(t) = I_0 \sin(2\pi f t), \qquad \textbf{(eq. S12)}$$

where f is frequency.

To find the total charge Q transferred during a single unidirectional polarization event (one half-cycle), we integrate the current over the half-period from $t = 0$ to $t = 1/(2f)$:

$$Q = \int_0^{1/(2f)} I(t)\mathrm{d}t = I_0/(\pi f) \qquad \textbf{(eq. S13)}$$

Using Faraday's law, the total thickness change Δd of the lithium layer plated or stripped per half-cycle is determined by dividing the plated volume by the electrode area:

$$\Delta d = \frac{Q \cdot M_{\text{Li}}}{A \cdot F \cdot \rho_{\text{Li}}} = \frac{U_0 \cdot \sigma_{\text{SE}} \cdot M_{\text{Li}}}{L \cdot \pi \cdot f \cdot F \cdot \rho_{\text{Li}}}, \qquad \textbf{(eq. S14)}$$

where M_{Li}, F, and ρ_{Li} are the molar mass of lithium, Faraday's constant and the density of lithium, respectively.

With M_{Li} = 6.94 g/mol, ρ_{Li} = 0.534 g/cm^3, F = 96485 C/mol and an excitation voltage U_0 of 10 mV, f = 100 mHz corresponds to ≈ 0.6 nm (1-2 atomic layers), whereas 10 mHz, 1 mHz, and 0.1 mHz correspond to ≈6 nm, ≈60 nm, and ≈600 nm, respectively.

This estimation assumes ideal planar lithium deposition and that the applied voltage drops entirely across the bulk solid electrolyte resistance.

S16. Summary Table and Literature Overview

Table S1. Literature Overview. Pressure values marked with an asterisk (*) denote joining pressures and pressure values without an asterisk denote stack pressures.

Study	Method	*P*/MPa	Relevant Findings
Kremer and Alt *et al.* (this work)	EIS	1, 5, 10, 20, 20*, 40*, 75*, 350*	Demonstrates that apparent SEI growth rate estimates are affected by contact conditions. Shows that native passivation layers can hinder SEI growth *and* can cause an *apparent* saturation in SEI resistance growth. Derives models to explain the experimental impedance evolution of Li|Li_6PS_5Cl|Li cells based on contact conditions. Highlights challenges in EIS analysis and provides an explanation for the pressure dependence observed in CTTA. Compares rate constants derived from both CTTA and impedance to theoretical predictions from the Wagner model.
Wenzel *et al.*[20] 2015	EIS	≈ 1.6	Evaluated SEI formation between $Li_7P_3S_{11}$ and lithium metal using EIS. Observed an impedance increase of Li|$Li_7P_3S_{11}$|Li cells. Attributed impedance increase to SEI growth. Observed a "stabilization" of the cell impedance after a few hours. Did not address the influence of pressure and contact conditions.
Wenzel *et al.*[21] 2016	EIS	≈ 1.6	Measured the impedance of Li|$Li_{10}GeP_2S_{12}$|Li cells over time. Found a faster impedance growth for $Li_{10}GeP_2S_{12}$-containing cells compared to $Li_7P_3S_{11}$-containing cells, consistent with the formation of an electronically conductive Li-Ge alloy degradation product. Reported a square-root-of-time dependence for SEI resistance evolution and verified impedance results via cyclic voltammetry. Did not address the influence of pressure and contact conditions.
Wenzel *et al.*[15] 2017	EIS	≈ 1.6	Investigated the impedance evolution of the argyrodite family (Li_6PS_5X) in contact with lithium metal. Highlighted the influence of specific halogens and their corresponding lithium halides on reaction kinetics. Reported a square-root-of-time dependence for SEI resistance evolution. Proposed the Wagner model to describe the growth trends. Pressure and contact conditions were not addressed.
Riegger *et al.*[22] 2020	EIS	12.7	Investigated the impedance evolution of Li|Li_3InCl_6|Li and Li|Li_3YCl_6|Li cells. Observed a non-linear continuous impedance increase for Li_3InCl_6, attributed to SEI formation. Conversely, Li|Li_3YCl_6|Li exhibited a continuous decrease in impedance, indicating the formation of a highly electronically conductive degradation product. Did not address the influence of pressure and contact conditions.
Zou *et al.*[23] 2021	EIS	/	Investigated the impedance evolution of Li|Li_6PS_5Cl|Li cells. Reported a square-root-of-time dependence for the extracted SEI resistance evolution. Did not address the influence of stack pressure and contact conditions.
Riegger *et al.*[24] 2022	EIS	6.4	Observed a non-linear and continuous impedance increase for Li|Li_7SiPS_8|Li cells. Hypothesized that phosphidosilicates provide sufficient electronic conductivity to enable continuous interphase growth, distinguishing this solid electrolyte from pure Li-P-S systems. Did not address the influence of pressure and contact conditions.
Riegger *et al.*[17] 2023	EIS	6.4, 12.7, 19.1, 38.2	Investigated the impedance evolution of Li|Li_6PS_5Cl|Li cells. Observed variance in rate constants and a saturation of impedance growth. Found these to be strongly influenced by factors such as applied stack pressure and the initial surface passivation of the lithium foils. *This work is the primary motivation for the current study*.
Aktekin *et al.*[18] 2023	CTTA	13	Introduced CTTA and demonstrated that the cumulative charge for Li_6PS_5Cl can be well described using a square-root-of-time law for SEI formation.
Sivavec *et al.*[19] 2025	CTTA	1.7, 13, 30	Demonstrated that CTTA results for symmetrical Li|Li_6PS_5Cl|Li cells are dependent on the applied stack-pressure. Showed that increasing stack-pressure leads to faster degradation.
Burton *et al.*[25] 2025	EIS during CTTA	13	Elucidated the role of phosphorus in the SEI growth of Li_6PS_5Cl. Measured impedance during CTTA, observing a deviation from a square-root-of-time dependence in the first 20 hours of the experiment. Explored whether a Deal-Grove-type model could explain the observed trends.

S17. Parameter Tables

Table S2. Series Model Fit Parameters (eq. S10) for Cells Evaluated in a Press-Cell-Type Setup. For pressure values marked with an asterisk (*), only data points between 6 and 10 days were fitted. Errors denote standard fitting errors.

**P/MPa**	**k'_{exp}/($\Omega\cdot cm^2\cdot s^{-0.5}$)**	**R_{off}/($\Omega\cdot cm^2$)**	**t_{off}/s**	**RMSE/($\Omega\cdot cm^2$)**
1	$(3.84 \pm 0.02) \cdot 10^{-2}$	$(8.29 \pm 0.02) \cdot 10^{1}$	$(4 \pm 2) \cdot 10^{3}$	1.52
1*	$(5.9 \pm 0.2) \cdot 10^{-2}$	$(6.6 \pm 0.3) \cdot 10^{1}$	$(0 \pm 4) \cdot 10^{4}$	0.23
5	$(9.9 \pm 0.1) \cdot 10^{-3}$	$(2.873 \pm 0.009) \cdot 10^{1}$	$(0 \pm 2) \cdot 10^{3}$	1.12
5*	$(2.55 \pm 0.06) \cdot 10^{-2}$	$(1.6 \pm 0.1) \cdot 10^{1}$	$(0 \pm 3) \cdot 10^{4}$	0.09
10	$(1.519 \pm 0.007) \cdot 10^{-2}$	$(1.161 \pm 0.006) \cdot 10^{1}$	$(4 \pm 1) \cdot 10^{3}$	0.54
10*	$(2.23 \pm 0.07) \cdot 10^{-2}$	$(6 \pm 1) \cdot 10^{0}$	$(0 \pm 4) \cdot 10^{4}$	0.09
20	$(3.22 \pm 0.01) \cdot 10^{-3}$	$(6.23 \pm 0.01) \cdot 10^{0}$	$(4 \pm 1) \cdot 10^{3}$	0.09
20*	$(4.9 \pm 0.2) \cdot 10^{-3}$	$(4.8 \pm 0.3) \cdot 10^{0}$	$(0 \pm 5) \cdot 10^{4}$	0.03

Table S3. Parallel Model Fit Parameters (eq. S11) for Cells Evaluated in a Pouch-Cell-Type Setup Without Applying Stack-Pressure. Errors denote standard fitting errors.

**P/MPa**	**k'_{exp}/($\Omega\cdot cm^2\cdot s^{-0.5}$)**	**R_{cstr}/($\Omega\cdot cm^2$)**	**R_{p*}/($\Omega\cdot cm^2$)**	**t_{off}/s**	**RMSE/($\Omega\cdot cm^2$)**
20	$(6.17 \pm 0.04) \cdot 10^{-1}$	$(5.0 \pm 0.1) \cdot 10^{1}$	$(7.517 \pm 0.005) \cdot 10^{1}$	$(3.6 \pm 0.2)\ 10^{3}$	0.17
20	$(2.4 \pm 0.1) \cdot 10^{-1}$	$(5.8 \pm 0.2) \cdot 10^{1}$	$(5.75 \pm 0.04) \cdot 10^{1}$	$(2 \pm 9)\ 10^{3}$	0.75
20	$(2.6 \pm 0.1) \cdot 10^{-1}$	$(6.4 \pm 0.1) \cdot 10^{1}$	$(6.40 \pm 0.03) \cdot 10^{1}$	$(2 \pm 8)\ 10^{3}$	0.73
40	$(8.3 \pm 0.1) \cdot 10^{-1}$	$(3 \pm 3) \cdot 10^{1}$	$(4.233 \pm 0.004) \cdot 10^{1}$	$(3.6 \pm 0.6)\ 10^{3}$	0.30
40	$(1.800 \pm 0.008) \cdot 10^{-1}$	$(24.0 \pm 0.2) \cdot 10^{1}$	$(3.230 \pm 0.002) \cdot 10^{1}$	$(5 \pm 1)\ 10^{4}$	0.06
40	$(1.487 \pm 0.008) \cdot 10^{-1}$	$(29.1 \pm 0.1) \cdot 10^{1}$	$(3.886 \pm 0.004) \cdot 10^{1}$	$(2 \pm 1)\ 10^{3}$	0.08
75	$(2.02 \pm 0.03) \cdot 10^{-1}$	$(23.6 \pm 0.6) \cdot 10^{1}$	$(2.280 \pm 0.003) \cdot 10^{1}$	$(2 \pm 3)\ 10^{3}$	0.13
75	$(2.11 \pm 0.04) \cdot 10^{-1}$	$(52.8 \pm 0.5) \cdot 10^{1}$	$(3.293 \pm 0.006) \cdot 10^{1}$	$(2 \pm 3)\ 10^{4}$	0.14
75	$(1.28 \pm 0.08) \cdot 10^{-1}$	$(19.1 \pm 0.1) \cdot 10^{1}$	$(2.390 \pm 0.002) \cdot 10^{1}$	$(2 \pm 1)\ 10^{3}$	0.06
75	$(1.45 \pm 0.02) \cdot 10^{-1}$	$(12.1 \pm 0.5) \cdot 10^{1}$	$(1.542 \pm 0.002) \cdot 10^{1}$	$(3.6 \pm 0.5)\ 10^{4}$	0.07
350	$(5.90 \pm 0.05) \cdot 10^{-2}$	$(3.5 \pm 0.1) \cdot 10^{0}$	$(6.477 \pm 0.006) \cdot 10^{0}$	$(2 \pm 2)\ 10^{3}$	0.03
350	$(1.11 \pm 0.02) \cdot 10^{-1}$	$(0.0 \pm 0.3) \cdot 10^{0}$	$(3.753 \pm 0.003) \cdot 10^{0}$	$(8 \pm 2)\ 10^{4}$	0.03
350	$(1.12 \pm 0.02) \cdot 10^{-1}$	$(0.0 \pm 0.4) \cdot 10^{0}$	$(2.810 \pm 0.002) \cdot 10^{0}$	$(3 \pm 2)\ 10^{3}$	0.02
350	$(3.70 \pm 0.07) \cdot 10^{-2}$	$(6.5 \pm 0.1) \cdot 10^{0}$	$(7.35 \pm 0.02) \cdot 10^{0}$	$(2 \pm 4)\ 10^{4}$	0.05

Table S4. Series Model Fit Parameters (eq. S10) for Cells Evaluated in a Pouch-Cell-Type Setup Without Applying Stack-Pressure. For pressure values marked with an asterisk (*), only data points between 2 and 10 days were fitted. Errors denote standard fitting errors.

***P*/MPa**	$k'_{exp}/(\Omega\cdot cm^2\cdot s^{-0.5})$	$R_{off}/(\Omega\cdot cm^2)$	t_{off}/s	$RMSE/(\Omega\cdot cm^2)$
20	(1.92 ±0.02) ·10^{-2}	(5.10 ±0.02) ·10^{1}	(0±3) 10^{3}	1.59
20*	(1.18 ±0.03) ·10^{-2}	(5.65 ±0.04) ·10^{1}	(0±2) 10^{3}	0.36
20	(1.14 ±0.01) ·10^{-2}	(3.56 ±0.01) ·10^{1}	(0±2) 10^{3}	1.06
20*	(1.05 ±0.06) ·10^{-2}	(3.83 ±0.08) ·10^{1}	(0±5) 10^{4}	0.77
20	(1.59 ±0.02) ·10^{-2}	(3.95 ±0.01) ·10^{1}	(0±2) 10^{3}	1.14
20*	(1.15 ±0.06) ·10^{-2}	(4.28 ±0.08) ·10^{1}	(0±5) 10^{4}	0.76
40	(8.1 ±0.1) ·10^{-3}	(3.35 ±0.01) ·10^{1}	(0±4) 10^{3}	1.14
40*	(3.1 ±0.2) ·10^{-3}	(3.72 ±0.03) ·10^{1}	(0±7) 10^{4}	0.30
40	(8.98 ±0.09) ·10^{-3}	(2.006±0.007)·10^{1}	(0±2) 10^{3}	0.64
40*	(6.0 ±0.1) ·10^{-3}	(2.26 ±0.02) ·10^{1}	(0±1) 10^{4}	0.13
40	(1.106±0.009)·10^{-2}	(2.208±0.007)·10^{1}	(0±2) 10^{3}	0.64
40*	(8.1 ±0.1) ·10^{-3}	(2.43 ±0.02) ·10^{1}	(0±1) 10^{4}	0.15
75	(5.13 ±0.06) ·10^{-3}	(1.630±0.005)·10^{1}	(0±3) 10^{3}	0.44
75*	(3.28 ±0.09) ·10^{-3}	(1.77 ±0.01) ·10^{1}	(0±2) 10^{4}	0.12
75	(6.33 ±0.05) ·10^{-3}	(2.37 ±0.04) ·10^{1}	(0±2) 10^{3}	0.38
75*	(4.79 ±0.06) ·10^{-3}	(2.470±0.007)·10^{1}	(0±1) 10^{4}	0.07
75	(6.53 ±0.06)·10^{-3}	(1.486±0.005)·10^{1}	(0±2) 10^{3}	0.44
75*	(4.65 ±0.08) ·10^{-3}	(1.62 ±0.01) ·10^{1}	(0±2) 10^{4}	0.11
75	(3.59 ±0.04) ·10^{-3}	(1.098±0.003) ·10^{1}	(0±3) 10^{3}	0.31
75*	(2.27 ±0.07) ·10^{-3}	(1,195±0.009)·10^{1}	(0±2) 10^{4}	0.08
350	(1.75 ±0.02) ·10^{-3}	(4.38 ±0.02) ·10^{0}	(0±3) 10^{3}	0.17
350*	(1.03 ±0.03) ·10^{-3}	(4.91 ±0.04) ·10^{0}	(0±3) 10^{4}	0.04
350	(5.8 ±0.1) ·10^{-4}	(3.16 ±0.01) ·10^{0}	(0±5) 10^{3}	0.10
350*	(2.3 ±0.3) ·10^{-4}	(3.41 ±0.03) ·10^{0}	(0±9) 10^{4}	0.03
350	(3.10 ±0.09) ·10^{-4}	(2.493±0.007)·10^{0}	(0±7) 10^{3}	0.07
350*	(5 ±2) ·10^{-5}	(2.68 ±0.02) ·10^{0}	(0±3) 10^{5}	0.02
350	(1.95 ±0.02) ·10^{-3}	(4.57 ±0.02) ·10^{0}	(0±2) 10^{3}	0.13
350*	(1.42 ±0.04) ·10^{-3}	(4.97 ±0.05) ·10^{0}	(0±2) 10^{4}	0.04

Table S5. Simplified Parallel Model Fit Parameters (eq. S11 without R_{cstr}) for Cells Evaluated in a Pouch-Cell-Type Setup Without Applying Stack-Pressure. Errors denote standard fitting errors.

***P*/MPa**	**k'_{exp}/(Ω·cm²·s⁻⁰·⁵)**	**R_{p*}/(Ω·cm²)**	**t_{off}/s**	**RMSE/(Ω·cm²)**
20	$(9.80 \pm 0.05) \cdot 10^{-1}$	$(7.176 \pm 0.005) \cdot 10^{1}$	$(3.6 \pm 1)\ 10^{3}$	0.67
20	$(6.81 \pm 0.08) \cdot 10^{-1}$	$(5.071 \pm 0.008) \cdot 10^{1}$	$(3.6 \pm 3)\ 10^{3}$	1.06
20	$(7.49 \pm 0.08) \cdot 10^{-1}$	$(5.645 \pm 0.008) \cdot 10^{1}$	$(3.6 \pm 3)\ 10^{3}$	1.13
40	$(8.53 \pm 0.04) \cdot 10^{-1}$	$(4.226 \pm 0.002) \cdot 10^{1}$	$(3.6 \pm 1)\ 10^{3}$	0.30
40	$(3.39 \pm 0.02) \cdot 10^{-1}$	$(2.990 \pm 0.003) \cdot 10^{1}$	$(3.6 \pm 2)\ 10^{3}$	0.34
40	$(3.49 \pm 0.02) \cdot 10^{-1}$	$(3.427 \pm 0.004) \cdot 10^{1}$	$(3.6 \pm 2)\ 10^{3}$	0.51
75	$(3.63 \pm 0.02) \cdot 10^{-1}$	$(2.171 \pm 0.002) \cdot 10^{1}$	$(3.6 \pm 2)\ 10^{3}$	0.22
75	$(6.47 \pm 0.06) \cdot 10^{-1}$	$(3.011 \pm 0.002) \cdot 10^{1}$	$(3.6 \pm 2)\ 10^{3}$	0.38
75	$(2.57 \pm 0.02) \cdot 10^{-1}$	$(2.196 \pm 0.002) \cdot 10^{1}$	$(3.6 \pm 2)\ 10^{3}$	0.26
75	$(2.37 \pm 0.01) \cdot 10^{-1}$	$(1.478 \pm 0.001) \cdot 10^{1}$	$(3.6 \pm 2)\ 10^{3}$	0.15
350	$(7.85 \pm 0.03) \cdot 10^{-2}$	$(6.303 \pm 0.003) \cdot 10^{0}$	$(3.6 \pm 1)\ 10^{3}$	0.04
350	$(1.111 \pm 0.007) \cdot 10^{-1}$	$(3.753 \pm 0.002) \cdot 10^{0}$	$(7.6 \pm 6)\ 10^{2}$	0.03
350	$(1.122 \pm 0.009) \cdot 10^{-1}$	$(2.810 \pm 0.001) \cdot 10^{0}$	$(2.8 \pm 5)\ 10^{2}$	0.02
350	$(8.35 \pm 0.07) \cdot 10^{-2}$	$(6.681 \pm 0.008) \cdot 10^{0}$	$(3.6 \pm 2)\ 10^{3}$	0.10

Table S6. CTTA Fit Parameters. Rate constants in rows marked with an asterisk (*) were derived from the data of Sivavec et al.[19] Data points were extracted from the original published figure and fitted using a Wagner-type model. The derivation of k_{exp} and k'_{exp} from $k_{Q,\mathrm{exp}}$ relies on multiple assumptions with unknown error margins. Consequently, specific error values are withheld, and the resulting estimates are denoted by the "≈" symbol.

***P*/MPa**	**$k_{Q,\mathrm{exp}}$/(mAh·cm⁻²·s⁻⁰·⁵)**	**k_{exp}/(nm·s⁻⁰·⁵)**	**k'_{exp}/(Ω·cm²·s⁻⁰·⁵)**
0.7	$(1.73 \pm 0.02) \cdot 10^{-3}$	≈0.26	≈0.19
4.0	$(3.11 \pm 0.02) \cdot 10^{-3}$	≈0.47	≈0.40
5.2	$(3.53 \pm 0.03) \cdot 10^{-3}$	≈0.53	≈0.35
13	$(3.02 \pm 0.04) \cdot 10^{-3}$	≈0.45	≈0.34
13	$(3.25 \pm 0.03) \cdot 10^{-3}$	≈0.49	≈0.36
13	$(3.14 \pm 0.05) \cdot 10^{-3}$	≈0.47	≈0.35
13	$(3.05 \pm 0.04) \cdot 10^{-3}$	≈0.46	≈0.34
52	$(3.11 \pm 0.05) \cdot 10^{-3}$	≈0.47	≈0.35
1.7*	$(1.8) \cdot 10^{-3}$	≈0.27	≈0.20
13*	$(2.5) \cdot 10^{-3}$	≈0.38	≈0.28
30*	$(2.9) \cdot 10^{-3}$	≈0.44	≈0.32

S18. Resistance Curves, Fits, and Residuals

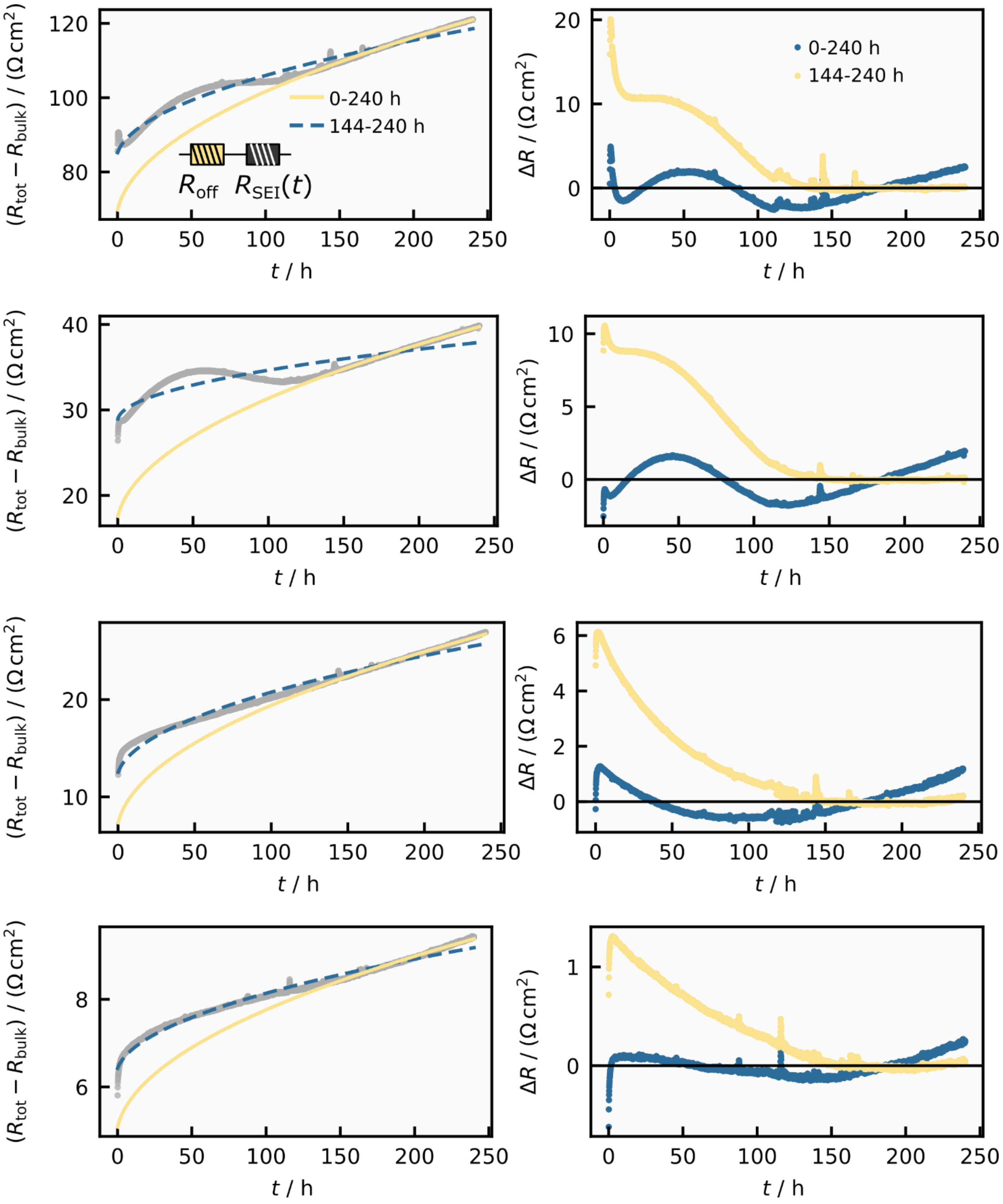

Figure S17. Series Wagner-Type Model Fits (left) and Color-Matched Residuals (right) for Cells in a Press-Cell-Type Setup. Applied pressures from top to bottom are 1 MPa, 5 MPa, 10 MPa, and 20 MPa. The experimental resistance data (grey) was fitted with equation S10 over the entire duration (blue dashed curves, 0-240 h) and selectively restricted to the final 4 days (yellow lines, 144–240 h).

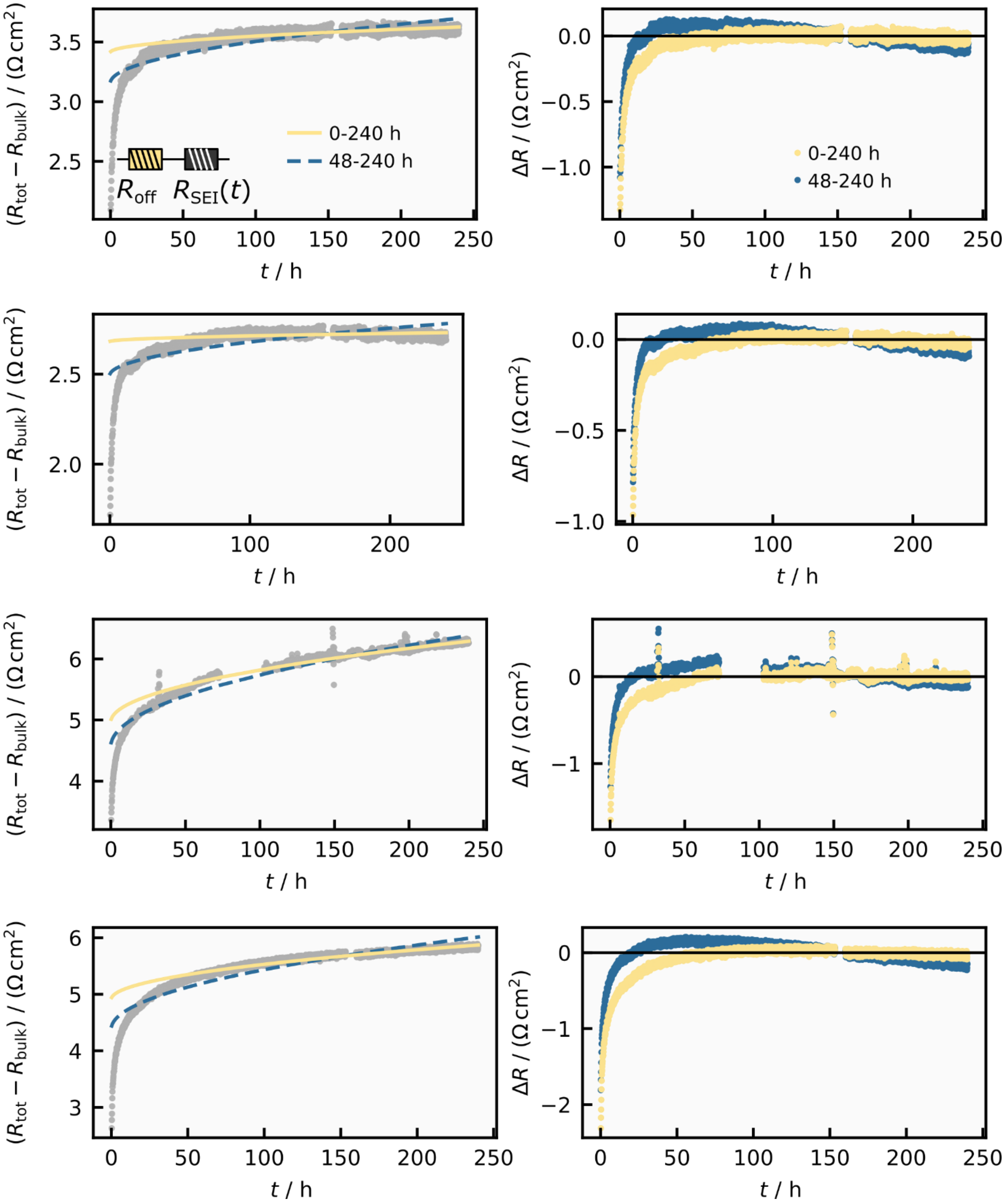

Figure S18. **Series Wagner-Type Model Fits (left) and Color-Matched Residuals (right) for Cells Isostatically Joined at 350 MPa and Evaluated in a Pouch-Cell-Type Setup Without Applying Stack-Pressure.** The experimental resistance data (grey) was fitted with equation S10 over the entire duration (blue dashed curves, 0-240 h) and selectively restricted to the final 8 days (yellow lines, 48–240 h).

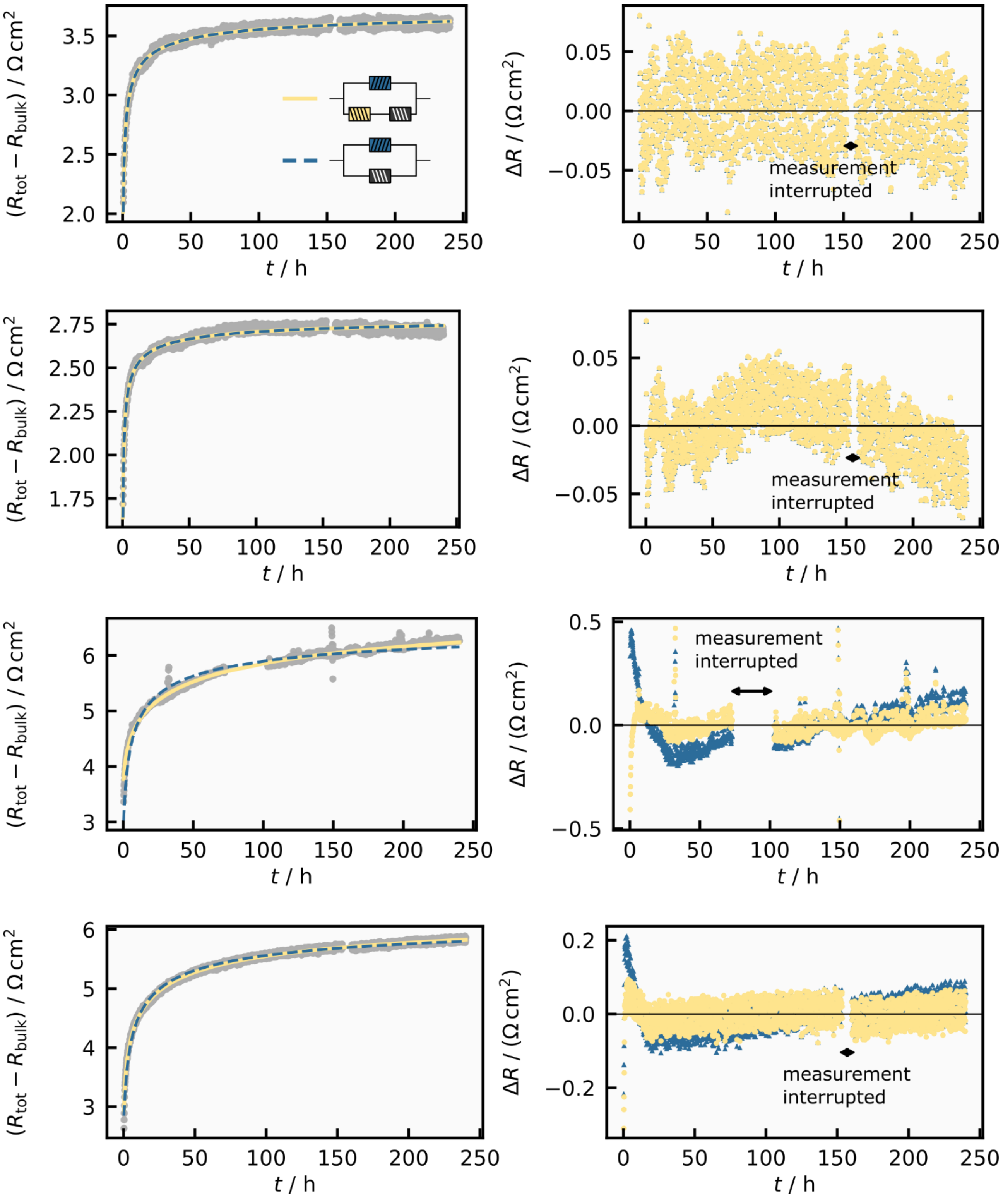

Figure S19. Parallel Model Fits (left) and Color-Matched Residuals (right) for Cells Isostatically Joined at 350 MPa and Evaluated in a Pouch-Cell-Type Setup Without Applying Stack-Pressure. The experimental resistance data (grey) was fitted with equation S11 (yellow lines, full parallel model) and equation S11 without R_{cstr} (blue dashed curves, reduced parallel model). All measurements at 350 MPa were interrupted for different timespans to investigate the impact on the impedance evolution. However, no systematic dependence/significant influence could be observed.

References

(1) Deal, B. E.; Grove, A. S. General Relationship for the Thermal Oxidation of Silicon. *Journal of Applied Physics* **1965**, *36* (12), 3770–3778. DOI: 10.1063/1.1713945.

(2) Wagner, C. Beitrag zur Theorie des Anlaufvorgangs. *Zeitschrift für Physikalische Chemie* **1933**, *21B* (1), 25–41. DOI: 10.1515/zpch-1933-2105.

(3) Bale, C. W.; Bélisle, E.; Chartrand, P.; Decterov, S. A.; Eriksson, G.; Gheribi, A. E.; Hack, K.; Jung, I.-H.; Kang, Y.-B.; Melançon, J.; Pelton, A. D.; Petersen, S.; Robelin, C.; Sangster, J.; Spencer, P.; van Ende, M.-A. FactSage Thermochemical Software and Databases, 2010–2016. *Calphad* **2016**, *54*, 35–53. DOI: 10.1016/j.calphad.2016.05.002.

(4) Eckhardt, J. K.; Klar, P. J.; Janek, J.; Heiliger, C. Interplay of Dynamic Constriction and Interface Morphology between Reversible Metal Anode and Solid Electrolyte in Solid State Batteries. *ACS Applied Materials & Interfaces* **2022**, *14* (31), 35545–35554. DOI: 10.1021/acsami.2c07077.

(5) Otto, S.-K.; Moryson, Y.; Krauskopf, T.; Peppler, K.; Sann, J.; Janek, J.; Henss, A. In-Depth Characterization of Lithium-Metal Surfaces with XPS and ToF-SIMS: Toward Better Understanding of the Passivation Layer. *Chem. Mater.* **2021**, *33* (3), 859–867. DOI: 10.1021/acs.chemmater.0c03518.

(6) Otto, S.-K.; Fuchs, T.; Moryson, Y.; Lerch, C.; Mogwitz, B.; Sann, J.; Janek, J.; Henss, A. Storage of Lithium Metal: The Role of the Native Passivation Layer for the Anode Interface Resistance in Solid State Batteries. *ACS Appl. Energy Mater.* **2021**, *4* (11), 12798–12807. DOI: 10.1021/acsaem.1c02481.

(7) Fujita, Y.; Kawasaki, Y.; Inaoka, T.; Kimura, T.; Sakuda, A.; Tatsumisago, M.; Hayashi, A. Amorphous Li_2O–LiI Solid Electrolytes Compatible to Li Metal. *Electrochemistry* **2021**, *89* (4), 334–336. DOI: 10.5796/electrochemistry.21-00049.

(8) Guo, R.; Gallant, B. M. Li_2O Solid Electrolyte Interphase: Probing Transport Properties at the Chemical Potential of Lithium. *Chem. Mater.* **2020**, *32* (13), 5525–5533. DOI: 10.1021/acs.chemmater.0c00333.

(9) Lorger, S.; Usiskin, R.; Maier, J. Transport and Charge Carrier Chemistry in Lithium Oxide. *J. Electrochem. Soc.* **2019**, *166* (10), A2215-A2220. DOI: 10.1149/2.1121910jes.

(10) Shi, S.; Qi, Y.; Li, H.; Hector, L. G. Defect Thermodynamics and Diffusion Mechanisms in Li_2CO_3 and Implications for the Solid Electrolyte Interphase in Li-Ion Batteries. *J. Phys. Chem. C* **2013**, *117* (17), 8579–8593. DOI: 10.1021/jp310591u.

(11) Deshpande, V.; Raghuwanshi, F.; Singh, K. Electrical Conductivity of the Li_2SO_4-LiOH System. *Solid State Ionics* **1986**, *18-19*, 378–381. DOI: 10.1016/0167-2738(86)90145-1.

(12) Dissanayake, M. Phase Diagram and Electrical Conductivity of the Li_2SO_4-Li_2CO_3 system. *Solid State Ionics* **1986**, *21* (4), 279–285. DOI: 10.1016/0167-2738(86)90190-6.

(13) Alt, C. D.; Müller, N. U.; Riegger, L. M.; Aktekin, B.; Minnmann, P.; Peppler, K.; Janek, J. Quantifying Multiphase SEI Growth in Sulfide Solid Electrolytes. *Joule* **2024**, *8* (10), 2755–2776. DOI: 10.1016/j.joule.2024.07.006.

(14) Wood, K. N.; Teeter, G. XPS on Li-Battery-Related Compounds: Analysis of Inorganic SEI Phases and a Methodology for Charge Correction. *ACS Appl. Energy Mater.* **2018**, *1* (9), 4493–4504. DOI: 10.1021/acsaem.8b00406.

(15) Wenzel, S.; Sedlmaier, S. J.; Dietrich, C.; Zeier, W. G.; Janek, J. Interfacial Reactivity and Interphase Growth of Argyrodite Solid Electrolytes at Lithium Metal Electrodes. *Solid State Ionics* **2018**, *318*, 102. DOI: 10.1016/j.ssi.2017.07.005.

(16) Jamnik, J. Impedance spectroscopy of mixed conductors with semi-blocking boundaries. *Solid State Ionics* **2003**, *157* (1-4), 19–28. DOI: 10.1016/S0167-2738(02)00183-2.

(17) Riegger, L. M.; Mittelsdorf, S.; Fuchs, T.; Rueß, R.; Richter, F. H.; Janek, J. Evolution of the Interphase between Argyrodite-Based Solid Electrolytes and the Lithium Metal Anode ─ The Kinetics of Solid Electrolyte Interphase Growth. *Chem. Mater.* **2023**, *35* (13), 5091–5099. DOI: 10.1021/acs.chemmater.3c00676.

(18) Aktekin, B.; Riegger, L. M.; Otto, S.-K.; Fuchs, T.; Henss, A.; Janek, J. SEI Growth on Lithium Metal Anodes in Solid-State Batteries Quantified with Coulometric Titration Time Analysis. *Nat Commun* **2023**, *14* (1), 6946. DOI: 10.1038/s41467-023-42512-y.

(19) Sivavec, J.; Kravchyk, K. V.; Kovalenko, M. V. Impact of Stack Pressure on Coulometric Titration Time Analysis. *Comm chemistry* **2025**, *8* (1), 96. DOI: 10.1038/s42004-025-01496-0.

(20) Wenzel, S.; Weber, D. A.; Leichtweiss, T.; Busche, M. R.; Sann, J.; Janek, J. Interphase Formation and Degradation of Charge Transfer Kinetics between a Lithium Metal Anode and Highly Crystalline $Li_7P_3S_{11}$ Solid Electrolyte. *Solid State Ionics* **2016**, *286*, 24–33. DOI: 10.1016/j.ssi.2015.11.034.

(21) Wenzel, S.; Randau, S.; Leichtweiß, T.; Weber, D. A.; Sann, J.; Zeier, W. G.; Janek, J. Direct Observation of the Interfacial Instability of the Fast Ionic Conductor $Li_{10}GeP_2S_{12}$ at the Lithium Metal Anode. *Chem. Mater.* **2016**, *28* (7), 2400–2407. DOI: 10.1021/acs.chemmater.6b00610.

(22) Riegger, L. M.; Schlem, R.; Sann, J.; Zeier, W. G.; Janek, J. Lithium-Metal Anode Instability of the Superionic Halide Solid Electrolytes and the Implications for Solid-State Batteries. *Angewandte Chemie (International ed. in English)* **2021**, *60* (12), 6718–6723. DOI: 10.1002/anie.202015238.

(23) Zou, C.; Yang, L.; Luo, K.; Liu, L.; Tao, X.; Yi, L.; Liu, X.; Luo, Z.; Wang, X. Ionic conductivity and interfacial stability of Li6PS5Cl–Li6.5La3Zr1.5Ta0.5O12 composite electrolyte. *J Solid State Electrochem* **2021**, *25* (10-11), 2513–2525. DOI: 10.1007/s10008-021-05004-x.

(24) Riegger, L. M.; Otto, S.-K.; Sadowski, M.; Jovanovic, S.; Kötz, O.; Harm, S.; Balzat, L. G.; Merz, S.; Burkhardt, S.; Richter, F. H.; Sann, J.; Eichel, R.-A.; Lotsch, B. V.; Granwehr, J.; Albe, K.;

Janek, J. Instability of the Li_7SiPS_8 Solid Electrolyte at the Lithium Metal Anode and Interphase Formation. *Chem. Mater.* **2022**, *34* (8), 3659. DOI: 10.1021/acs.chemmater.1c04302.

(25) Burton, M.; Jagger, B.; Liang, Y.; Gibson, J. S.; Aspinall, J.; Long, Z.; Swallow, J. E. N.; Weatherup, R. S.; Pasta, M. The Role of Phosphorus in the Solid Electrolyte Interphase of Argyrodite Solid Electrolytes. *Nature Communications* **2025**, *16* (1), 9304. DOI: 10.1038/s41467-025-64357-3.
